# Attitudes to mandatory COVID-19 vaccination in early life: findings from the multi-country cross-sectional CANDOUR study

**DOI:** 10.1093/heapro/daag077

**Published:** 2026-06-09

**Authors:** Georgia Porter, Laurence S J Roope, Mara Violato, Raymond Duch, Philip M Clarke, Marian Knight, Rema Ramakrishnan

**Affiliations:** Exeter College, University of Oxford, Turl Street, Oxford OX1 3DP, United Kingdom; Economics of Population Health, Oxford Population Health, University of Oxford, Old Road Campus, Richard Doll Building, Oxford OX3 7LF, United Kingdom; National Institute for Health Research Oxford Biomedical Research Centre, John Radcliffe Hospital, Headley Way, Oxford OX3 9DU, United Kingdom; Economics of Population Health, Oxford Population Health, University of Oxford, Old Road Campus, Richard Doll Building, Oxford OX3 7LF, United Kingdom; Nuffield College, University of Oxford, New Road, Oxford OX1 1NF, United Kingdom; Economics of Population Health, Oxford Population Health, University of Oxford, Old Road Campus, Richard Doll Building, Oxford OX3 7LF, United Kingdom; Centre for Health Policy, Melbourne School of Population and Global Health, University of Melbourne, Grattan Street, Parkville, Melbourne, VIC 3010, Australia; National Perinatal Epidemiology Unit, Oxford Population Health, University of Oxford, Old Road Campus, Richard Doll Building, Oxford OX3 7LF, United Kingdom; Economics of Population Health, Oxford Population Health, University of Oxford, Old Road Campus, Richard Doll Building, Oxford OX3 7LF, United Kingdom; National Perinatal Epidemiology Unit, Oxford Population Health, University of Oxford, Old Road Campus, Richard Doll Building, Oxford OX3 7LF, United Kingdom

**Keywords:** early life vaccination, COVID-19 vaccine, vaccine mandate, vaccine attitudes, CANDOUR

## Abstract

We examined variation in attitude toward mandatory COVID-19 vaccination in early life by sociodemographic characteristics, personal COVID-19 experience, health risk attitude, political ideology, and other COVID-19 vaccine mandate attitudes. For this purpose, we used data from 19 928 participants from 16 countries surveyed in March–November 2022 for the second wave of the COVID-19 vaccine preference and opinion survey (CANDOUR). Analyses were adjusted for poststratification weighting. Participants who disagreed with early life mandatory COVID-19 vaccination were more likely to decline vaccination against COVID-19 (14.5%) compared with those who were neutral (1.8%) and those who agreed (0.5%). Disagreement with early life mandatory vaccination was associated with more unwillingness to take risks with their own health, being centre or left-wing on the left-right political spectrum, believing COVID-19 vaccination should be a personal choice, and being opposed to vaccine mandates for schoolchildren and the public. Neutrality or agreement with early life mandatory vaccination was associated with neutrality or agreement with a vaccine mandate for schoolchildren or a governmental COVID-19 vaccine mandate for everybody. Pandemic preparedness governance needs to focus on attitudes toward vaccine mandates. Further research and commitment by governments at various levels are needed to identify social, cultural, and system-level factors that could inform vaccination strategies to be implemented for the next pandemic.

## Graphical Abstract

**Graphical Abstract:**
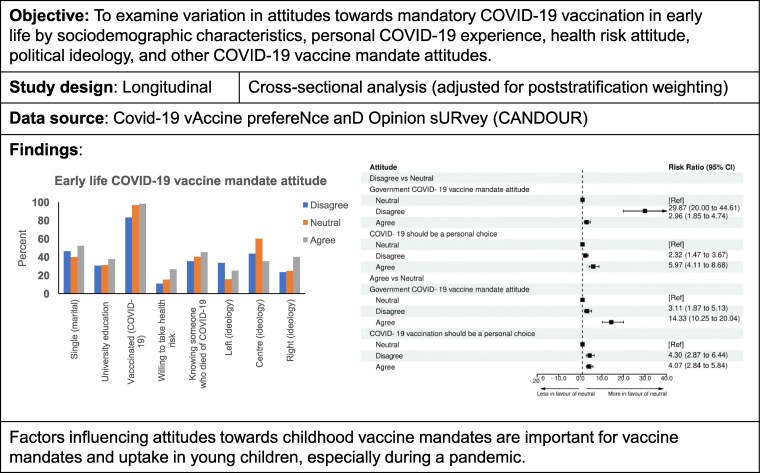

Contribution to Health Promotion

- There is limited information on attitudes about requiring COVID-19 vaccination for young children such as newborns, infants, and preschool children.
- Attitude toward COVID-19 vaccine mandates for young children is influenced by attitudes toward schoolchild and governmental COVID-19 vaccine mandates, personal COVID-19 vaccination status, and knowing someone who died from COVID-19.
- Attitudes toward childhood vaccines and their uptake may be important when considering vaccine requirements for young children, especially during a pandemic.

## Introduction

Though COVID-19 is no longer a public health emergency of worldwide concern, it continues to be a major public health issue (World Health Organization 2023). With over 779 million cases of COVID-19 and more than seven million COVID-19 deaths reported to the World Health Organization (WHO) as of 23 January 2026 (World Health Organization 2026), increasing COVID-19 vaccine uptake to reduce hospitalizations and deaths remains an important global health goal.

A controversial strategy suggested to raise COVID-19 vaccine coverage is mandatory vaccination, which can be defined as a government policy requiring individuals in certain groups—typically demarcated by profession or age—to be vaccinated. While vaccine mandates have historically been successful (Lee and Robinson 2016, Lin *et al*. 2016, Vaz *et al*. 2020, Ukonaho *et al*. 2022), they can cause antagonism. Vaccine mandates targeting children in particular have faced substantial parental opposition (Salmon *et al*. 2015, Smith *et al*. 2021). Various COVID-19 mandates have been proposed for government officials, healthcare workers, children, and the general public (Alahmad 2023, Cameron-Blake *et al*. 2023, Maneze *et al*. 2023), with occupation-based COVID-19 vaccine mandates more common than age-based mandates (Cameron-Blake *et al*. 2023).

A 2021 systematic review exploring attitudes toward mandatory childhood vaccination across seven countries found that parents preferred universal to targeted schemes and perceived vaccine mandate schemes as encroaching on their rights (Smith *et al*. 2021). While studies have reported attitudes toward COVID-19 vaccination in children aged 0–5 years (early life) (Al-Qerem *et al*. 2022, Demir Pervane *et al*. 2025), there is limited evidence about peoples’ attitudes toward early life mandatory COVID-19 vaccination and the characteristics of these individuals. Early life vaccine mandates are thought to be different from schoolchild mandates because they tend to focus on protecting the individual child whereas schoolchild mandates tend to focus on herd immunity. Yet, neonates, infants, and preschool children may be ideal candidates for a vaccine mandate: while young children display less vulnerability to severe and critical COVID-19 infection than adults (Götzinger *et al*. 2021), they are still at risk of multisystem inflammatory syndrome (Raschetti *et al*. 2020), and face a high risk of infection in daycare and preschool education environments (Nesti and Goldbaum 2007). Furthermore, national programs to distribute vaccines to children already exist in most countries, whereas Mast *et al*. (2005) reported that the infrastructure often does not support vaccination of “uninsured and underinsured adults” (Mast *et al*. 2005). Vaccinating children in early life would, therefore, be simpler to implement and could decrease risk of severe disease or death with subsequent infection in later childhood or adulthood, or indeed, risk of transmission to older vulnerable adults (Alahmad 2023, Cameron-Blake *et al*. 2023, Maneze *et al*. 2023).

Therefore, we aimed to assess how attitude toward early life mandatory COVID-19 vaccination varied by sociodemographic characteristics, personal COVID-19 experience, health risk attitude, political ideology, and other COVID-19 vaccine mandate attitudes. It is expected that the findings will translate into better pandemic preparedness by focusing on targeted interventions, improved policy design, and tailored communication strategies across the globe.

## Materials and methods

### Study design and participants

We conducted a cross-sectional analysis of data obtained from wave II of the COVID-19 vAccine prefereNce anD Opinion sURvey (CANDOUR) project—a longitudinal, multi-country, web-based survey designed to provide information on global issues related to health, economics, and politics in the wake of the COVID-19 pandemic (CANDOUR 2024). Data collection for wave II took place from 1 March 2022 until 18 November 2022. Inclusion criteria for the CANDOUR II study were: (i) age 18 or over; (ii) access to an electronic device connected to the internet; and (iii) residence in one of the 16 countries sampled (Australia, Brazil, Canada, Chile, China, Colombia, France, Ghana, India, Italy, Japan, South Africa, Spain, Uganda, UK, and USA). The CANDOUR project was approved by the University of Oxford Medical Sciences Interdivisional University Research Ethics Committee. All study participants provided written informed consent. This paper adheres to the Strengthening the Reporting of Observational Studies in Epidemiology guidelines.

The survey was administered online via an anonymous Qualtrics questionnaire. Participants were sampled by Respondi or Facebook Ad Manager. Quota sampling was used in all countries except Ghana, India, South Africa, and Uganda because it was recognized that it would not be nationally representative in these countries; instead, an internet survey was used. Quota sampling was used to ensure that the demographic spread of the sample reflected national distributions for age, gender, education, and macro-region. Furthermore, poststratification weighting was used in countries where imbalances persisted. The questionnaire was translated from English into six other languages (Chinese, French, Italian, Japanese, Portuguese, and Spanish) by professional translators and approved by native speakers to ensure fidelity. After completion of the survey, all respondents were provided with minimal financial compensation.

### Early life COVID-19 vaccine mandate attitude

Attitude toward mandatory COVID-19 vaccination in early life (0–5 years) was assessed by participant response to the statement “health clinics should be required by law to give a suitable version of a COVID-19 vaccine to all newborns, infants, and preschool children” using a sliding scale from 0 (very much disagree) to 100 (very much agree). Participants could select any integer value or answer “do not know.” For the analysis, this continuous variable was categorized into three groups: disagree (0–33), neutral (34–66), and agree (67–100) for better interpretability, especially for policy makers and public health professionals. This approach was taken for all variables that used a 0–100 sliding scale (0: very much disagree to 100: very much agree). We used this approach (absolute) instead of an approach based on the distribution of the variables such as categorizing into tertiles or quartiles so that the categories provide consistent interpretation, are comparable across countries within this study or future studies that might use these variables, and are not sample-dependent.

### Sociodemographic characteristics

Self-reported sociodemographic information was collected on age, gender, marital status, number of children, education level, employment status, adult household size, and gross annual household income. Age (years) was categorized into six groups (18–24, 25–34, 35–44, 45–54, 55–64, 65, and over). Marital status was categorized into single and not single. Number of children was categorized into four groups (0, 1, 2, 3, and over). Education level was gauged by highest educational or work-related qualification completed and consisted of four categories (less than primary completed, primary completed, secondary completed, and university completed). Two variables were used to measure employment status, which was split into employed and not employed. Total household size was calculated by adding adult household size and number of dependent children. If either variable was missing or had been answered “prefer not to say/don’t know,” total household size was coded as missing. As the phrasing of the adult household size question included the participant, a response of zero was interpreted as participants misreading the question and was changed to 1 before it was added to the number of dependent children. Total household sizes surpassing 20 were coded as 20: this value was chosen as a plausible household size maximum. Total household size was integrated with gross annual household income to establish equivalized gross annual income. A square root equivalence scale was used to reflect the disproportional increase in resource demand with increasing household size (OECD 2012). Dividing this variable by purchasing power parity (PPP) provided an additional income measure, standardized across countries: PPP-adjusted equivalized gross annual income. PPPs control for the varying price levels of goods between countries and are often preferable to market exchange rates due to their greater stability and inclusion of non-traded goods and services (Callen 2018). PPP-adjusted equivalized gross annual income for each country was stratified into country-specific quintiles for easy interpretability.

### Personal COVID-19 experience, health risk attitude, and political ideology

Personal COVID-19 experience was measured by questions that asked if participants were vaccinated against COVID-19, what side effects they experienced, their reasons for getting vaccinated, and proximity to COVID-19-related death. Proximity to COVID-19-related death was measured by asking whether participants had known “anyone who has died from COVID-19” (yes/no). Health risk attitude (willingness to take risks with their personal health) was recorded using a sliding scale from 0 (very unwilling) to 10 (very willing) and was divided into three categories: unwilling (0–3), neutral (4–6), and willing (7–10). Ideology was also measured on a 0–10 sliding scale and divided into three groups: left (0–3), centre (4–6), and right (7–10).

### Other COVID-19 vaccine mandate attitudes

Participant attitudes toward other COVID-19 vaccine mandates were assessed via three questions—whether the participant agreed with the statements: (i) All schoolchildren should be required by law to get a COVID-19 vaccine; (ii) The government should make COVID-19 vaccination mandatory for everybody; and (iii) Whether a person gets a COVID-19 vaccine or not should be a matter of personal choice. These variables were measured on a 0–100 sliding scale (0: very much disagree to 100: very much agree) and split into three categories [disagree (0–33), neutral (34–66), and agree (67–100)].

### Statistical analysis

Analyses were carried out using Stata 18.0 BE (StataCorp. 2023, Stata Statistical Software: Release 18, College Station, TX: StataCorp LLC). Descriptive statistics were calculated: unweighted frequencies and weighted percentages were used to examine early life COVID-19 vaccine mandate attitude by participant sociodemographic characteristics, personal COVID-19 experience, health risk attitude, ideology, and other COVID-19 vaccine mandate attitudes for the overall sample and for each country. Multinomial regression analyses were conducted to calculate adjusted risk ratios and associated 95% confidence intervals for the association between COVID-19 vaccination status and other COVID-19 vaccine mandate attitudes and early life COVID-19 vaccine mandate attitude only for the overall sample. These models were adjusted for country and all sociodemographic characteristics. Observations with missing values were excluded for the regression analyses. Among participants with available data on COVID-19 vaccine mandate attitudes (*n* = 18 069 for COVID-19 vaccination status; *n* = 19 478 for schoolchild vaccine mandates; *n* = 19 706 for governmental vaccine mandates; and *n* = 19 687 for attitudes toward COVID-19 vaccination as a personal choice), the proportions of missing data in the regression models were 9.7%, 9.6%, 10.1%, and 10.2%, respectively. As the CANDOUR II study used poststratification weighting to account for sample imbalances by macro-region, age, gender, and education level, the statistics presented are based on Stata commands for complex surveys.

## Results

### Sample characteristics

The analytical sample included 19 928 participants who answered the early life COVID-19 vaccine mandate attitude question. Of these, 50.1% were men, 49.6% women, and 0.3% in the other/not prefer to say category. The total column in Table 1 summarizes the sociodemographic characteristics (unweighted).

**Table 1:** Sociodemographic characteristics of participants in the CANDOUR II study by early life COVID-19 vaccine mandate attitude.

Early life COVID-19 vaccine mandate attitude	Disagree *n* = 8071	Neutral *n* = 4130	Agree *n* = 7727	Total *N* = 19 928	*P*-value
	Unweighted frequency (weighted %)	Unweighted frequency (weighted %)	Unweighted frequency (weighted %)	Unweighted frequency (unweighted %)	
Age (years)					.22
18–24	810 (15.5)	519 (15.0)	960 (19.3)	2289 (11.5)	
25–34	2216 (28.2)	1168 (27.7)	2076 (25.8)	5460 (27.4)	
35–44	1737 (20.3)	859 (14.2)	1482 (17.8)	4078 (20.5)	
45–54	1262 (14.8)	639 (17.3)	1183 (15.3)	3084 (15.5)	
55–64	1254 (10.1)	576 (15.1)	1285 (14.3)	3115 (9.5)	
65 and over	792 (11.1)	369 (10.8)	741 (7.6)	1902 (9.5)	
Gender					.13
Woman	4317 (45.5)	1944 (43.7)	3727 (36.6)	9988 (50.1)	
Man	3731 (42.1)	2178 (47.7)	3971 (46.9)	9880 (49.6)	
Other/Prefer not to say	23 (12.4)	8 (8.6)	29 (16.5)	60 (0.3)	
Marital status					.02
Single	3284 (46.1)	1630 (39.7)	2940 (52.0)	7854 (40.0)	
Not single	4667 (53.9)	2415 (60.3)	4712 (48.0)	11 794 (60.0)	
*Missing*	*120*	*85*	*75*	*280*	
Number of children					.08
0	4326 (55.0)	2231 (54.3)	4137 (58.7)	10 694 (54.4)	
1	1594 (18.1)	911 (20.2)	1813 (21.2)	4318 (22.0)	
2	1254 (13.2)	611 (14.9)	1162 (13.5)	3027 (15.4)	
3 and over	795 (13.7)	302 (10.6)	539 (6.6)	1636 (8.3)	
*Missing*	*102*	*75*	*76*	*253*	
Education level					.03
Less than primary completed	111 (2.8)	71 (7.5)	116 (2.6)	298 (1.5)	
Primary completed	508 (14.9)	325 (13.2)	684 (11.5)	1517 (7.7)	
Secondary completed	3609 (51.7)	1607 (48.2)	2714 (48.3)	7930 (40.0)	
University completed	3796 (30.5)	2108 (31.1)	4168 (37.6)	10 072 (50.8)	
*Missing*	*47*	*19*	*45*	*111*	
Employment status					.49
Employed	5182 (61.6)	2661 (61.2)	4994 (57.6)	12 837 (64.4)	
Not employed	2889 (38.4)	1469 (38.8)	2733 (42.4)	7091 (35.6)	
Quintiles for PPP-adjusted equivalized gross annual income					.44
Quintile 1 (low)	1463 (31.4)	879 (29.1)	1506 (34.1)	3848 (21.1)	
Quintile 2	1496 (21.1)	756 (22.6)	1440 (16.1)	3692 (20.2)	
Quintile 3	1456 (18.1)	761 (21.2)	1414 (16.8)	3631 (19.9)	
Quintile 4	1543 (16.4)	752 (14.6)	1490 (18.7)	3785 (20.8)	
Quintile 5 (high)	1272 (13.1)	673 (12.5)	1344 (14.3)	3289 (18.1)	
*Missing*	*841*	*309*	*533*	*1683*	

Frequencies are unweighted and percentages are weighted, except for total column, in which both are unweighted.

Percentages do not sum to 100 due to rounding. Italics indicate frequency without the percentage.

### Sociodemographic characteristics and early life COVID-19 vaccine mandate attitude

Overall, there were no significant differences in early life COVID-19 vaccine mandate attitude by sociodemographic characteristics except for marital status and education (Table 1). However, variations were observed between countries for some characteristics such as gender in Chile (Supplementary Table S10), Italy (Supplementary Table S28), and Uganda (Supplementary Table S40), age in Canada (Supplementary Table S7), Colombia (Supplementary Table S16), Italy (Supplementary Table S28), and South Africa (Supplementary Table S34), number of children in Chile (Supplementary Table S10), Colombia (Supplementary Table S16), France (Supplementary Table S19), Japan (Supplementary Table S31), and Spain (Supplementary Table S37), employment status in South Africa (Supplementary Table S34) and USA (Supplementary Table S46) and gross annual income in France (Supplementary Table S19), India (Supplementary Table S25), and South Africa (Supplementary Table S34). Overall, participants were more likely to be single (52.0%) if they agreed with mandatory COVID-19 vaccination in early life, but the finding was reversed for the “neutral” (not single: 60.3%) and “disagree” groups (not single: 53.9%). Those agreeing with an early life COVID-19 vaccine mandate were also proportionally the highest educated, with 85.9% having attained secondary or university-level education (neutral: 79.3%; disagree: 82.2%).

### Personal COVID-19 experience, health risk attitude, political ideology, and early life COVID-19 vaccine mandate attitude

**Figure 1:**
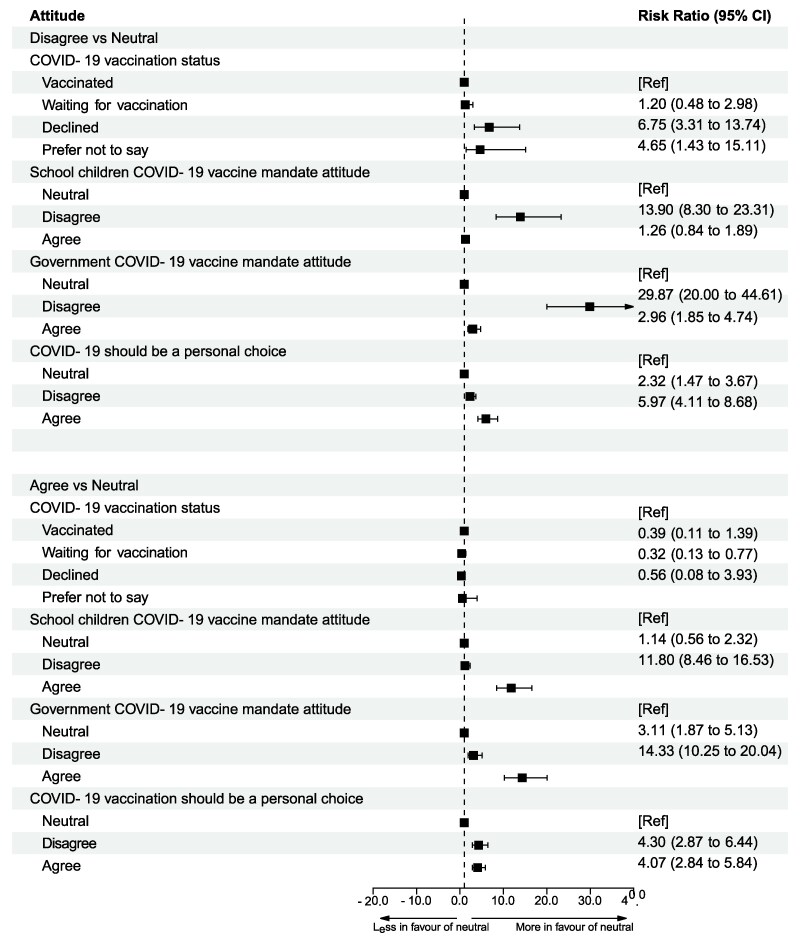
Forest plot of adjusted risk ratios for factors associated with early life COVID-19 vaccine mandate attitude.

The group that disagreed with the vaccine mandate had the largest proportion of participants who declined vaccination (14.5%) with a risk ratio of 6.75 (95% CI 3.31–13.74) for disagree vs neutral (Table 2 and Fig. 1). Furthermore, if already vaccinated, a higher proportion of participants who disagreed with the mandate had experienced side effects (disagree: 81.6%; neutral: 70.1; agree: 71.4%). A higher proportion of participants in the disagree group were motivated by the ability to travel and visit people or places (disagree: 43.4%; neutral: 39.4%; agree: 36.9%) and by work/school vaccination requirements (disagree: 20.8%; neutral: 13.6%; agree: 16.7%) rather than the neutral and agree groups, who were proportionally more motivated by the protection of themselves, their families, and the public. However, there were significant differences observed for some countries. For example, in Australia (Supplementary Table S2), Canada (Supplementary Table S8), India (Supplementary Table S26), and the USA (Supplementary Table S47), a higher proportion of participants who agreed were motivated by recommendations by politicians compared with participants who disagreed. However, in Brazil (Supplementary Table S5), Japan (Supplementary Table S32), and the USA (Supplementary Table S47) a higher proportion of participants who agreed were motivated by recommendations by healthcare professionals compared with participants who disagreed. Overall, in all vaccine mandate attitude groups, the majority were unwilling or neutral to taking risks with their health, though the proportion of unwilling participants was greatest in the group disagreeing with mandatory vaccination in early life (61.2%) and lowest in the “neutral” group (45.0%) (Table 2). The disagree group was also least likely to have known anyone who died of COVID-19 (35.3% versus 40.2% for neutral and 45.0% for agree). Political centrists made up the largest proportion of participants in the disagree (43.5%) and neutral (59.8%) groups, whereas the right was the largest group amongst those agreeing with an early life vaccine mandate (39.9%) (Table 2).

**Table 2:** Personal COVID-19 experience, health risk attitude, and political ideology of participants in the CANDOUR II study by early life COVID-19 vaccine mandate attitude.

Early life COVID-19 vaccine mandate attitude	Disagree *n* = 8071	Neutral *n* = 4130	Agree *n* = 7727	Total *N* = 19 928	*P*-value
Number of participants	Unweighted frequency (weighted %)	Unweighted frequency (weighted %)	Unweighted frequency (weighted %)	Unweighted frequency (unweighted %)	
COVID-19 vaccination status					<.0001
Vaccinated	6175 (83.1)	3440 (96.8)	7050 (98.3)	16 665 (92.2)	
Waiting for vaccination	104 (0.9)	46 (1.0)	50 (1.0)	200 (1.1)	
Declined	936 (14.5)	85 (1.8)	59 (0.5)	1080 (6.0)	
Prefer not to say	92 (1.4)	18 (0.4)	14 (0.2)	124 (0.7)	
*Missing*	*764*	*541*	*554*	*1859*	
Side effects					.002
No side effects	1226 (18.4)	938 (29.9)	2206 (28.6)	4370 (26.2)	
Side effects	4949 (81.6)	2502 (70.1)	4844 (71.4)	12 295 (73.8)	
Not vaccinated	1132	149	123	1404	
*Missing*	*764*	*541*	*554*	*1859*	
Reasons for vaccinating					
To protect myself	4211 (69.1)	2771 (80.2)	6025 (76.9)	13 007 (78.1)	.03
To protect my family	3940 (58.4)	2541 (68.6)	5450 (71.6)	11 931 (71.6)	.003
To protect the public	2301 (35.4)	1424 (36.3)	3442 (45.5)	7167 (43.0)	.02
To travel and visit people/places	2652 (43.4)	1451 (39.4)	3175 (36.9)	7278 (47.7)	.25
Because everyone else will	294 (6.1)	290 (11.0)	757 (11.8)	1341 (8.1)	.08
Recommended by friends/family	310 (6.4)	278 (4.7)	52 (1.0)	1340 (8.0)	.04
Recommended by healthcare officials/professionals	1746 (24.9)	1166 (29.2)	2778 (32.3)	5690 (34.1)	.11
Recommended by politicians	240 (2.8)	217 (10.0)	652 (8.1)	1109 (6.7)	.01
Contact with/symptoms of COVID-19	567 (12.1)	428 (11.7)	1185 (12.6)	2180 (13.1)	.95
Work/school requirement	14 452 (20.8)	548 (13.6)	1231 (16.7)	3231 (19.4)	.09
Other reason(s)	193 (4.1)	41 (0.6)	66 (1.3)	300 (1.8)	.02
Personal health risk attitude					<.0001
Unwilling	5029 (61.2)	1774 (45.0)	3986 (49.2)	10 789 (55.6)	
Neutral	2012 (28.0)	1616 (39.7)	1546 (24.3)	5174 (26.7)	
Willing	795 (10.8)	613 (15.2)	2018 (26.6)	3426 (17.7)	
*Missing*	*235*	*127*	*177*	*539*	
Does participant know anyone who died of COVID-19?					.03
Yes	2996 (35.3)	1649 (40.2)	3634 (45.0)	8279 (42.7)	
No	4839 (64.7)	2325 (59.8)	3936 (55.0)	11 100 (57.3)	
*Missing*	*236*	*156*	*157*	*549*	
Ideology					<.0001
Left	1825 (33.4)	741 (15.5)	1433 (24.9)	3999 (22.9)	
Centre	3495 (43.5)	1986 (59.8)	2693 (35.2)	8174 (46.8)	
Right	1893 (23.2)	885 (24.7)	2515 (39.9)	5293 (30.3)	
*Missing*	*858*	*518*	*1086*	*2462*	

Frequencies are unweighted and percentages are weighted, except for total column, in which both are unweighted. Italics indicate frequency without the percentage.

### Other COVID-19 vaccine mandates and early life COVID-19 vaccine mandate attitude

Most participants who disagreed with early life mandatory vaccination also disagreed with mandatory COVID-19 vaccination for schoolchildren or a governmental COVID-19 vaccine mandate for everyone. Similarly, most people who were neutral toward or agreed with early life mandatory vaccination were neutral toward or agreed with these other mandates, respectively (Table 3). Compared with participants who were neutral, participants who disagreed with mandatory vaccination for schoolchildren were nearly 14 times (95% CI 8.30–23.31) more likely to disagree with an early life vaccine mandate rather than be neutral (Fig. 1). Similarly, compared with participants who were neutral, participants who agreed with mandatory vaccination for schoolchildren were nearly 12 (95% CI 8.46–16.53) times more likely to agree with the early life vaccine mandate rather than be neutral (Fig. 1). Those who agreed or disagreed with an early life vaccine mandate were most likely to agree that COVID-19 vaccination should be a personal choice (agree: 45.9%; disagree: 60.9%). In contrast, most neutral participants remained neutral on COVID-19 vaccination being a personal choice (53.2%) (Table 3). Compared with participants who were neutral, participants who agreed that COVID-19 vaccination should be a personal choice were six times more likely to disagree (95% CI: 4.11–8.68) or four times more likely to agree (95% CI 2.84–5.84) with early life vaccine mandate rather than be neutral (Fig. 1).

**Table 3:** Other COVID-19 vaccine mandate attitudes of participants in the CANDOUR II study by early life COVID-19 vaccine mandate attitude.

Early life COVID-19 vaccine mandate attitude	Disagree *n* = 8071	Neutral *n* = 4130	Agree *n* = 7727	Total *N* = 19 928
	Unweighted frequency (weighted %)	Unweighted frequency (weighted %)	Unweighted frequency (weighted %)	Unweighted frequency (unweighted %)
Schoolchild COVID-19 vaccine mandate attitude				
Disagree	5225 (69.6)	488 (16.7)	362 (2.6)	6075 (31.2)
Neutral	1338 (17.2)	1981 (50.4)	950 (10.3)	4269 (21.9)
Agree	12 607 (13.2)	1572 (32.8)	6295 (87.1)	9134 (46.9)
*Missing*	*241*	*89*	*120*	*450*
Governmental COVID-19 vaccine mandate attitude				
Disagree	5862 (74.4)	580 (11.9)	578 (7.4)	7020 (35.6)
Neutral	1043 (11.8)	2093 (55.1)	916 (13.8)	4052 (20.6)
Agree	1086 (13.8)	1404 (33.1)	6144 (78.8)	8634 (43.8)
*Missing*	*80*	*53*	*89*	*222*
COVID-19 vaccination should be a personal choice				
Disagree	1812 (22.6)	875 (22.2)	2642 (37.1)	5329 (27.1)
Neutral	1330 (16.5)	1978 (53.2)	1329 (17.0)	4637 (23.5)
Agree	4877 (60.9)	1219 (24.6)	3625 (45.9)	9721 (49.4)
*Missing*	*52*	*58*	*131*	*241*

Frequencies are unweighted and percentages are weighted, except for total column, in which both are unweighted.

All *P*-values are <.0001. Italics indicate frequency without the percentage.

## Discussion

### Main findings

This multi-country study from 16 countries showed that early life COVID-19 vaccine mandate attitude varies with marital status, education level, COVID-19 vaccination status, experience of COVID-19 vaccination side effects, reasons for vaccinating, health risk attitude, political ideology, knowing anyone who died of COVID-19, schoolchild and governmental COVID-19 vaccine mandate attitudes, and attitude toward COVID-19 vaccination being a personal choice.

### Comparison with other studies

While few, if any studies in this field have focused specifically on factors associated with early life COVID-19 vaccine mandate attitudes, a systematic review of 16 studies reported that vaccine acceptance and hesitancy varied with marital status and education level (Limbu *et al*. 2022), the same sociodemographic characteristics identified in our study but for attitudes toward mandatory vaccination. Harris *et al*. (2023) showed that within the USA, participants were more likely to support a COVID-19 vaccine mandate if they were liberal, vaccine-accepting, and had positive views toward COVID-19 vaccines (Harris *et al*. 2023). If the label “liberal” is taken to describe those belonging to the centre-left, then our results differed: we found that there was a higher proportion of those on the left within the “disagree” group compared with the “neutral” and “agree” groups. This could be due to the foundational belief in right to bodily autonomy associated with left and liberal stances (Levey 2012).

Limbu *et al*. (2022) also found age, gender, employment status, and income to be associated with vaccine acceptance, hesitancy, and uptake. We did observe differences by age, gender, employment status, and income but only for few countries in our study. This could be explained by the different focus questions of the studies: for example, older age may well be associated with increased vaccine acceptance or uptake as individuals want to protect themselves, whereas a vaccine mandate for the youngest in society is unlikely to affect their personal safety, thus, may not inspire such strong feeling. Gender, employment status, and income could be associated with vaccine acceptance and uptake in different ways: further research may provide clarity.

Most studies about attitudes toward mandatory vaccination sample only healthcare workers or parents. Studies in healthcare workers have shown that this group tends to support a healthcare worker-specific COVID-19 vaccine mandate, but are less decided on a broader mandate encompassing the general public (Politis *et al*. 2023). By contrast, parents tend to be against mandatory vaccination for their children as it “infringes on their rights” (Smith *et al*. 2021). Though our study focused on attitudes toward vaccination—specifically, mandatory vaccination in early life—rather than vaccination behavior, studies have demonstrated an association between positive vaccination attitudes and vaccine uptake (Smith *et al*. 2017, Harris *et al*. 2023) and indeed the opposite: lower vaccine uptake is associated with valuing the parents’ right to choose (i.e. rather than mandating vaccination) (Brown *et al*. 2010). Even within this analysis, a link between attitude and vaccine-related behavior can be seen: 14.5% of respondents who disagreed with early life mandatory vaccination declined to be vaccinated, whereas the unvaccinated group accounted for <3% of the combined neutral and agree groups. Accordingly, factors affecting attitudes are likely to affect behavior.

Our study focused mainly on factors surrounding an individual’s responsibility for health and does not necessarily capture social and cultural factors, or erosion of trust in scientific evidence-based policies (Bardosh *et al*. 2022) that are essential for pandemic preparedness governance. Strategies that do not specifically focus on individuals’ attitudes and behaviors are needed to address vaccine hesitancy, increase uptake, and minimize distrust, such as those proposed in the components of the diffusion of innovation model (Rogers 1995), the social–ecological model (McLeroy *et al*. 1988), and the Vaccine Trust Framework (The Vaccine Trust Project no date). Most of the components of these models/framework are likely to be bypassed when vaccines are mandated. Therefore, pandemic preparedness should involve important and influential individuals in communities who could enable other individuals to make informed decisions. In addition, policies and research need to focus on specific interpersonal, organizational, and political factors as well as social networks that could influence vaccine uptake. It would be useful to identify population-specific drivers of health system promise, health system delivery, vaccine promise, and vaccine delivery (The Vaccine Trust Project no date). This would enable individuals to make informed decisions about vaccines and minimize the need for system-level mandates and apparently sudden decisions during a pandemic.

### Strengths and limitations

This study had several strengths. Participants were sampled from countries representing over half the world’s population and sample sizes were larger than those used in similar studies (Askarian *et al*. 2022, Candio *et al*. 2023). Quota sampling was used in 12 of the countries in an effort to obtain representative samples. Poststratification survey weights were constructed to counter non-response bias that was accounted for in the analyses. Methodological differences across countries are likely to be small because most questions and methods used in our study were standardized.

A limitation was that, being a cross-sectional study, there is likely to be temporality bias. The sample may not be representative of the populations from which they were drawn, though this was somewhat mitigated by the use of quota sampling and poststratification weighting. Furthermore, people were excluded from the survey if they were outside the age range, did not have access to an electronic device, or had inadequate internet-literacy, which may have excluded older groups or those of lower socioeconomic status. Bias was also introduced by the questionnaire languages and recruitment platforms used: Facebook-based recruitment may select individuals with more extremist views around vaccination (Schmidt *et al*. 2018). The validity of the 0–100 feeling thermometer scales has also been called into question. Although web-based feeling thermometer data collection is more reliable than face-to-face and is easier to respond to than open-ended numeric text input (Liu and Wang 2015, Liu and Conrad 2016), it increases break-off rate for respondents with low formal education and lengthens task duration when compared with radio button scales (Funke *et al*. 2011). The use of a single question to assess attitude toward early life COVID-19 vaccine mandate attitude is somewhat reductive. The statement used to explore this attitude contains legal, practical, and ethical aspects, each of which participants may have interpreted or responded to in different ways. Including more questions around the reasons for agreement/disagreement with an early life mandate would have helped with interpretation and thus application, as these reasons may differ from those motivating personal vaccination—for example, concerns about autonomy in early life, the relatively low incidence and severity of COVID-19 infection in this group, and sparsity of safety studies in children. Some countries did not use quota sampling, which might have expected to contribute to heterogeneity between countries. However, the results suggest quota sampling alone does not fully explain those differences. Ethnicity was excluded from the analysis as it was asked inconsistently between countries. The questionnaire did not differentiate between finer categories of education and employment, especially, healthcare-related versus non-healthcare-related and employment, which could have affected health literacy and likely influenced vaccination attitudes (Fukuda *et al*. 2021, Khoury *et al*. 2024). Ideology is also dependent on the culture, history, and current political situation of the country, which may limit the generalizability of comparison between countries. The ideology question was not asked in China; therefore, results for ideology did not include China. Coding answers of “do not know” and “prefer not to say” as missing risks excluding meaningful responses and introducing bias. Though the percentage of missing values in the regression models was around 10% the inclusion of participants with only complete data in the regression models could have biased the results. We controlled for potential confounders in the regression analyses; however, residual confounding cannot be ruled out. Categorization of variables measured on a 0–100 sliding scale could have led to loss of information, statistical power, and bias due to the use of arbitrary cutoffs. Furthermore, the risk ratios associated with wide confidence intervals—possibly because they are based on relatively small numbers in the multivariable models—must be interpreted with caution. Concerns over the safety of the COVID-19 vaccine in young children may also have influenced vaccination attitudes given the suspension of the AstraZeneca COVID-19 vaccine in 2021 across parts of Europe due to emerging knowledge of severe side effects (Wise 2021).

### Implications

Future research should focus on understanding vaccine mandate attitudes within key population subgroups—such as pregnant women, women of reproductive age, and healthcare workers—to support the development of proportionate and acceptable vaccination policies if mandates become necessary. Consistent with WHO guidance that mandates be considered only when voluntary uptake and less intrusive strategies are insufficient (World Health Organization 2022), mandatory vaccination may nonetheless represent an important public health option for achieving population-level protection in the context of future pandemics.

The COVID-19 pandemic is no longer classified as a public health emergency of international concern (World Health Organization 2023). With COVID-19-related hospitalizations and deaths declining, less contentious strategies than mandatory vaccination can be used to effectively increase vaccine uptake (Becchetti *et al*. 2021) and lessen the global disease burden, including emphasizing to unvaccinated and hesitant groups—as have been identified by this study and others—the health risks of remaining unvaccinated and the benefits and safety of the COVID-19 vaccine. Past evidence has shown that interventions should be tailored to the reason for non-vaccination to be most effective—moderate and extreme vaccine hesitancy are driven by different factors thereby requiring different approaches (Betsch *et al*. 2015, Candio *et al*. 2023). Betsch *et al*. (2015) recommend focusing on removing practical barriers to vaccination and motivating the complacent, perhaps through incentives, during which period it is imperative to regularly monitor fluctuations in societal attitudes toward vaccination, remaining informed and ready to act if the situation changes (Betsch *et al*. 2015).

## Conclusion

Attitude toward mandatory COVID-19 vaccination for newborns, infants, and preschool children varies by marital status, education level, personal COVID-19 experience, health risk attitude, political ideology, and other COVID-19 vaccine mandate attitudes. These findings enable identification of vaccine-hesitant individuals around which to focus vaccination campaigns. Research on attitudes toward vaccination and vaccine mandates can strengthen the evidence base for planning the implementation of new vaccination schedules to minimize inequalities in uptake and, consequently, health outcomes if routine childhood vaccine schedules need updating or another pandemic occurs.

## Supplementary Material

daag077_Supplementary_Data

## Data Availability

The datasets used/analyzed during the current study are available from the corresponding author on reasonable request.
