# Supplementary material for: Attitudes to mandatory COVID-19 vaccination in early life: findings from the multi-country cross-sectional CANDOUR study
Source: Health Promot Int. 2026 Jun 9;41(3):daag077. doi: 10.1093/heapro/daag077 (PMC13247998; doi:10.1093/heapro/daag077)
Supplement: daag077_Supplementary_Data [file daag077_supplementary_data.docx]

**SUPPLEMENTARY TABLES**

**AUSTRALIA**

**Table S1** Sociodemographic characteristics of participants in the CANDOUR II study (Australia) by early life COVID-19 vaccine mandate attitude

| **Early life COVID-19 vaccine mandate attitude** | **Disagree**  **n=686** | **Neutral**  **n=342** | **Agree**  **n=647** | **Total**  **N=1,675** | **P-value** |
| --- | --- | --- | --- | --- | --- |
|  | Unweighted frequency **(weighted %)** | Unweighted frequency **(weighted %)** | Unweighted frequency **(weighted %)** | Unweighted frequency **(unweighted %)** |  |
| **Age (years)** |  |  |  |  | 0.16 |
| 18-24 | 41 (7.6) | 25 (31.9) | 34 (12.9) | 100 (6.0) |  |
| 25-34 | 175 (20.5) | 88 (19.4) | 112 (33.4) | 375 (22.4) |  |
| 35-44 | 161 (26.7) | 86 (15.8) | 142 (25.5) | 142 (23.2) |  |
| 45-54 | 107 (12.7) | 40 (9.4) | 122 (10.0) | 122 (18.9) |  |
| 55-64 | 105 (10.6) | 57 (13.6) | 107 (8.5) | 107 (16.5) |  |
| 65 and over | 97 (21.9) | 46 (10.0) | 130 (9.9) | 130 (20.1) |  |
| **Gender** |  |  |  |  | 0.63 |
| Woman | 488 (58.3) | 214 (63.1) | 376 (47.3) | 1,078 (64.4) |  |
| Man | 197 (30.3) | 128 (36.9) | 269 (36.2) | 594 (35.5) |  |
| Other/Prefer not to say | 1 (11.3) | 0 (0.0) | 2 (16.5) | 3 (0.2) |  |
| **Marital status** |  |  |  |  | 0.24 |
| Single | 248 (45.2) | 118 (28.8) | 241 (50.8) | 607 (36.7) |  |
| Not single | 432 (54.8) | 217 (71.2) | 397 (49.2) | 1,046 (63.3) |  |
| *Missing* | *6* | *7* | *9* | *22* |  |
| **Number of children** |  |  |  |  | 0.29 |
| 0 | 406 (71.1) | 209 (72.6) | 438 (61.5) | 1,053 (63.4) |  |
| 1 | 113 (12.2) | 58 (12.7) | 91 (14.4) | 262 (15.8) |  |
| 2 | 105 (10.7) | 52 (10.4) | 84 (22.3) | 241 (14.5) |  |
| 3 and over | 57 (6.0) | 18 (4.3) | 29 (1.7) | 104 (6.3) |  |
| *Missing* | *5* | *5* | *5* | *15* |  |
| **Education level** |  |  |  |  | 0.08 |
| Less than primary completed | 3 (0.5) | 1 (0.3) | 3 (0.5) | 7 (0.4) |  |
| Primary completed | 129 (22.7) | 57 (24.6) | 77 (13.1) | 263 (15.8) |  |
| Secondary completed | 282 (56.0) | 116 (44.1) | 233 (48.8) | 631 (37.8) |  |
| University completed | 271 (20.7) | 167 (31.0) | 330 (37.6) | 768 (46.0) |  |
| *Missing* | *1* | *1* | *4* | *6* |  |
| **Employment status** |  |  |  |  | 0.54 |
| Employed | 414 (55.7) | 200 (44.0) | 368 (59.9) | 982 (58.6) |  |
| Not employed | 272 (44.3) | 142 (56.0) | 279 (40.1) | 693 (41.4) |  |
| **Quintiles for PPP-adjusted equivalised gross annual income** |  |  |  |  | 0.28 |
| Quintile 1 (low) | 118 (28.2) | 73 (20.5) | 117 (33.0) | 308 (20.4) |  |
| Quintile 2 | 124 (17.2) | 62 (15.2) | 111 (22.6) | 297 (19.6) |  |
| Quintile 3 | 128 (15.3) | 56 (12.6) | 116 (22.6) | 300 (19.8) |  |
| Quintile 4 | 132 (13.6) | 54 (12.8) | 119 (11.0) | 305 (20.2) |  |
| Quintile 5 (high) | 113 (25.8) | 65 (38.9) | 124 (10.8) | 302 (20.0) |  |
| *Missing* | *71* | *32* | *60* | *163* |  |

Notes: Frequencies are unweighted and percentages are weighted, except for total column, in which both are unweighted.

Percentages do not sum to 100 due to rounding

| **Table S2.** Personal COVID-19 experience, health risk attitude, and political ideology of participants in the CANDOUR II study (Australia) by early life COVID-19 vaccine mandate attitude | | | | | |
| --- | --- | --- | --- | --- | --- |
| **Early life COVID-19 vaccine mandate attitude** | **Disagree**  **n=686** | **Neutral**  **n=342** | **Agree**  **n=647** | **Total**  **N=1,675** | **P-value** |
| **Number of participants** | Unweighted frequency **(weighted %)** | Unweighted frequency **(weighted %)** | Unweighted frequency **(weighted %)** | Unweighted frequency **(unweighted %)** |  |
| **COVID-19 vaccination status** |  |  |  |  | <0.001 |
| Vaccinated | 549 (85.1) | 312 (94.5) | 634 (99.5) | 1,495 (92.1) |  |
| Waiting for vaccination | 6 (1.3) | 3 (2.3) | 1 (0.1) | 10 (0.6) |  |
| Declined | 103 (13.2) | 8 (3.3) | 1 (0.1) | 112 (6.9) |  |
| Prefer not to say | 6 (0.5) | 0 (0.0) | 1 (0.2) | 7 (0.4) |  |
| *Missing* | *22* | *19* | *10* | *51* |  |
| **Side Effects** |  |  |  |  | 0.92 |
| No side effects | 90 (26.2) | 62 (23.2) | 157 (24.2) | 309 (20.7) |  |
| Side effects | 459 (73.8) | 250 (76.8) | 477 (75.8) | 1,186 (79.3) |  |
| Not vaccinated | 115 | 11 | 3 | 129 |  |
| *Missing* | *22* | *19* | *10* | *51* |  |
| **Reasons for Vaccinating** |  |  |  |  |  |
| To protect myself | 378 (75.5) | 257 (82.3) | 580 (86.0) | 1,215 (81.3) | 0.40 |
| To protect my family | 335 (69.0) | 253 (79.8) | 527 (71.3) | 1,115 (74.6) | 0.60 |
| To protect the public | 245 (44.1) | 166 (54.1) | 387 (58.9) | 798 (53.4) | 0.37 |
| To travel and visit people/places | 286 (63.6) | 184 (57.5) | 387 (41.9) | 857 (57.3) | 0.08 |
| Because everyone else will | 16 (1.7) | 29 (10.1) | 83 (16.8) | 128 (8.6) | 0.004 |
| Recommended by friends/family | 29 (3.1) | 29 (9.3) | 72 (15.9) | 130 (8.7) | 0.03 |
| Recommended by healthcare officials/professionals | 223 (56.0) | 144 (46.2) | 348 (38.3) | 715 (47.8) | 0.21 |
| Recommended by politicians | 25 (3.5) | 23 (8.9) | 87 (17.8) | 135 (9.0) | 0.01 |
| Contact with/symptoms of COVID-19 | 41 (18.7) | 42 (12.8) | 109 (18.8) | 192 (12.8) | 0.81 |
| Work/school requirement | 168 (36.5) | 59 (17.4) | 112 (17.5) | 339 (22.7) | 0.39 |
| Other reason(s) | 26 (3.6) | 8 (2.6) | 13 (0.8) | 47 (3.1) | 0.003 |
| **Personal health risk attitude** |  |  |  |  | 0.01 |
| Unwilling | 402 (55.1) | 148 (45.0) | 355 (43.3) | 905 (54.7) |  |
| Neutral | 220 (37.1) | 146 (41.7) | 179 (22.9) | 545 (32.9) |  |
| Willing | 57 (7.7) | 39 (13.3) | 109 (33.9) | 205 (12.4) |  |
| *Missing* | *7* | *9* | *4* | *20* |  |
| **Does participant know anyone who died of COVID-19?** |  |  |  |  | 0.08 |
| Yes | 64 (6.6) | 60 (34.2) | 122 (18.1) | 246 (14.9) |  |
| No | 611 (93.4) | 275 (65.8) | 513 (81.9) | 1,399 (85.1) |  |
| *Missing* | *11* | *7* | *12* | *30* |  |
| **Ideology** |  |  |  |  | 0.23 |
| Left | 129 (13.8) | 71 (15.5) | 118 (25.0) | 318 (19.0) |  |
| Centre | 438 (61.7) | 200 (69.3) | 343 (43.3) | 981 (58.6) |  |
| Right | 119 (24.6) | 71 (15.2) | 186 (31.7) | 376 (22.5) |  |
| *Missing* | *0* | *0* | *0* | *0* |  |

Note: Frequencies are unweighted and percentages are weighted, except for total column, in which both are unweighted. Percentages do not sum to 100 due to rounding

**Table S3.** Other COVID-19 vaccine mandate attitudes of participants in the CANDOUR II study (Australia) by early life

| **Early life COVID-19 vaccine mandate attitude** | **Disagree**  **n=8,071** | **Neutral**  **n=4,130** | **Agree**  **n=7,727** | **Total**  **N=19,928** | **P-value** |
| --- | --- | --- | --- | --- | --- |
|  | Unweighted frequency **(weighted %)** | Unweighted frequency **(weighted %)** | Unweighted frequency **(weighted %)** | Unweighted frequency **(unweighted %)** |  |
| **Schoolchild COVID-19 vaccine mandate attitude** |  |  |  |  |  |
| Disagree | 454 (51.7) | 37 (7.8) | 15 (1.3) | 506 (31.0) |  |
| Neutral | 108 (25.2) | 152 (59.2) | 75 (5.9) | 335 (20.5) | <0.001 |
| Agree | 107 (23.1) | 144 (33.1) | 542 (92.9) | 793 (48.5) |  |
| *Missing* | *17* | *9* | *15* | *41* |  |
| **Governmental COVID-19 vaccine mandate attitude** |  |  |  |  |  |
| Disagree | 512 (58.2) | 50 (11.8) | 29 (11.6) | 591 (35.7) |  |
| Neutral | 91 (10.8) | 163 (37.7) | 87 (7.0) | 341 (20.6) | <0.001 |
| Agree | 75 (31.0) | 127 (50.5) | 524 (81.4) | 726 (43.8) |  |
| *Missing* | *8* | *2* | *7* | *17* |  |
| **COVID-19 vaccination should be a personal choice** |  |  |  |  |  |
| Disagree | 125 (25.2) | 88 (21.1) | 265 (40.4) | 478 (29.0) | 0.05 |
| Neutral | 134 (26.3) | 155 (58.2) | 123 (19.6) | 412 (25.0) |  |
| Agree | 421 (48.6) | 91 (20.7) | 245 (40.0) | 757 (46.0) |  |
| *Missing* | *6* | *8* | *14* | *28* |  |

Notes: Frequencies are unweighted and percentages are weighted, except for total column, in which both are unweighted.

Percentages do not sum to 100 due to rounding

**BRAZIL**

**Table S4.** Sociodemographic characteristics of participants in the CANDOUR II study (Brazil) by early life COVID-19 vaccine mandate attitude

| **Early life COVID-19 vaccine mandate attitude** | **Disagree**  **n=353** | **Neutral**  **n=229** | **Agree**  **n=739** | **Total**  **N=1,321** | **P-value** |
| --- | --- | --- | --- | --- | --- |
|  | Unweighted frequency **(weighted %)** | Unweighted frequency **(weighted %)** | Unweighted frequency **(weighted %)** | Unweighted frequency **(unweighted %)** |  |
| **Age (years)** |  |  |  |  | 0.60 |
| 18-24 | 42 (26.6) | 38 (14.2) | 85 (32.2) | 165 (12.5) |  |
| 25-34 | 90 (30.4) | 76 (37.4) | 167 (19.3) | 333 (25.2) |  |
| 35-44 | 82 (7.9) | 40 (13.2) | 158 (13.5) | 280 (21.2) |  |
| 45-54 | 60 (17.1) | 34 (3.0) | 131 (12.7) | 225 (17.0) |  |
| 55-64 | 52 (4.7) | 30 (13.1) | 142 (13.7) | 224 (17.0) |  |
| 65 and over | 27 (13.3) | 11 (19.2) | 56 (8.6) | 94 (7.1) |  |
| **Gender** |  |  |  |  | 0.19 |
| Woman | 192 (53.7) | 129 (61.6) | 382 (36.9) | 703 (53.2) |  |
| Man | 160 (36.2) | 99 (29.3) | 357 (63.1) | 616 (46.6) |  |
| Other/Prefer not to say | 1 (10.1) | 1 (9.2) | 0 (0.0) | 2 (0.2) |  |
| **Marital status** |  |  |  |  | 0.27 |
| Single | 140 (46.6) | 89 (43.3) | 310 (65.7) | 539 (41.9) |  |
| Not single | 210 (53.4) | 116 (56.7) | 423 (34.3) | 749 (58.2) |  |
| *Missing* | *3* | *24* | *6* | *33* |  |
| **Number of children** |  |  |  |  | 0.19 |
| 0 | 194 (62.4) | 115 (73.4) | 435 (64.2) | 744 (57.5) |  |
| 1 | 83 (19.4) | 55 (7.9) | 182 (22.6) | 320 (24.7) |  |
| 2 | 60 (6.4) | 30 (16.3) | 85 (11.5) | 175 (13.5) |  |
| 3 and over | 14 (11.9) | 13 (2.2) | 29 (1.7) | 56 (4.3) |  |
| *Missing* | *2* | *16* | *8* | *26* |  |
| **Education level** |  |  |  |  | 0.21 |
| Less than primary completed | 34 (34.4) | 32 (21.0) | 65 (20.1) | 131 (10.1) |  |
| Primary completed | 41 (22.7) | 28 (20.2) | 52 (16.9) | 121 (9.3) |  |
| Secondary completed | 143 (29.8) | 96 (50.8) | 317 (43.8) | 556 (42.8) |  |
| University completed | 130 (13.1) | 66 (8.1) | 294 (19.3) | 490 (37.8) |  |
| *Missing* | *5* | *7* | *11* | *23* |  |
| **Employment status** |  |  |  |  | 0.77 |
| Employed | 203 (29.6) | 124 (40.4) | 444 (37.9) | 771 (58.4) |  |
| Not employed | 150 (70.4) | 105 (59.6) | 295 (62.1) | 550 (41.6) |  |
| **Quintiles for PPP-adjusted equivalised gross annual income** |  |  |  |  | 0.21 |
| Quintile 1 (low) | 61 (39.2) | 58 (34.0) | 119 (54.3) | 238 (20.0) |  |
| Quintile 2 | 62 (25.3) | 61 (22.6) | 147 (21.6) | 270 (22.7) |  |
| Quintile 3 | 66 (23.7) | 33 (27.1) | 135 (6.9) | 234 (19.6) |  |
| Quintile 4 | 65 (6.4) | 29 (2.9) | 155 (13.2) | 249 (20.9) |  |
| Quintile 5 (high) | 60 (5.3) | 22 (13.4) | 119 (4.0) | 201 (16.9) |  |
| *Missing* | *39* | *26* | *64* | *129* |  |

Notes: Frequencies are unweighted and percentages are weighted, except for total column, in which both are unweighted.

Percentages do not sum to 100 due to rounding

| **Table S5.** Personal COVID-19 experience, health risk attitude, and political ideology of participants in the CANDOUR II study (Brazil) by early life COVID-19 vaccine mandate attitude | | | | | |
| --- | --- | --- | --- | --- | --- |
| **Early life COVID-19 vaccine mandate attitude** | **Disagree**  **n=353** | **Neutral**  **n=229** | **Agree**  **n=739** | **Total**  **N=1,321** | **P-value** |
| **Number of participants** | Unweighted frequency **(weighted %)** | Unweighted frequency **(weighted %)** | Unweighted frequency **(weighted %)** | Unweighted frequency **(unweighted %)** |  |
| **COVID-19 vaccination status** |  |  |  |  | <0.001 |
| Vaccinated | 290 (95.6) | 179 (99.2) | 710 (99.8) | 1,179 (97.8) |  |
| Waiting for vaccination | 5 (1.2 | 1 (0.3) | 2 (0.001) | 8 (0.7) |  |
| Declined | 14 (3.0) | 0 (0.0) | 1 (0.0001) | 15 (1.2) |  |
| Prefer not to say | 2 (0.2) | 2 (0.1) | 0 (0.0) | 4 (0.3) |  |
| *Missing* | *42* | *47* | *26* | *115* |  |
| **Side Effects** |  |  |  |  | 0.001 |
| No side effects | 217 (87.7) | 130 (51.2) | 538 (89.2) | 885 (75.1) |  |
| Side effects | 73 (12.3) | 49 (48.9) | 172 (10.8) | 294 (24.9) |  |
| Not vaccinated | 21 | 3 | 3 | 27 |  |
| *Missing* | *42* | *47* | *26* | *115* |  |
| **Reasons for Vaccinating** |  |  |  |  |  |
| To protect myself | 189 (81.6) | 153 (95.9) | 609 (69.5) | 951 (80.7) | 0.005 |
| To protect my family | 177 (62.6) | 133 (50.2) | 561 (2.7) | 871 (73.9) | 0.16 |
| To protect the public | 85 (27.7) | 68 (40.5) | 327 (41.8) | 480 (40.7) | 0.73 |
| To travel and visit people/places | 68 (27.8) | 55 (24.2) | 212 (29.4) | 335 (28.4) | 0.94 |
| Because everyone else will | NA | NA | NA | NA |  |
| Recommended by friends/family | 14 (2.4) | 7 (1.0) | 56 (10.2) | 77 (6.5) | 0.002 |
| Recommended by healthcare officials/professionals | 47 (6.5) | 45 (8.7) | 225 (24.1) | 317 (26.9) | 0.006 |
| Recommended by politicians | 4 (0.4) | 5 (0.8) | 26 (2.2) | 35 (3.0) | 0.03 |
| Contact with/symptoms of COVID-19 | 20 (3.5) | 21 (18.9) | 110 (13.7) | 151 (12.8) | 0.33 |
| Work/school requirement | 48 (7.0) | 14 (2.5) | 62 (4.1) | 124 (10.5) | 0.15 |
| Other reason(s) | 14 (2.5) | 3 (0.8) | 9 (0.5) | 26 (2.2) | 0.01 |
| **Personal health risk attitude** |  |  |  |  | 0.49 |
| Unwilling | 227 (57.4) | 101 (53.0) | 471 (44.4) | 799 (63.9) |  |
| Neutral | 73 (24.9) | 88 (43.9) | 127 (42.5) | 288 (23.0) |  |
| Willing | 30 (17.7) | 27 (3.2) | 106 (13.1) | 163 (13.0) |  |
| *Missing* | *23* | *13* | *35* | *71* |  |
| **Does participant know anyone who died of COVID-19?** |  |  |  |  | 0.30 |
| Yes | 246 (51.5) | 159 (65.4) | 597 (76.4) | 1,002 (78.8) |  |
| No | 92 (48.5) | 51 (34.6) | 127 (23.6) | 270 (21.2) |  |
| *Missing* | *15* | *19* | *15* | *49* |  |
| **Ideology** |  |  |  |  | 0.08 |
| Left | 38 (28.8) | 35 (5.1) | 254 (27.3) | 327 (28.5) |  |
| Centre | 80 (24.4) | 87 (71.4) | 209 (48.9) | 376 (32.7) |  |
| Right | 193 (46.8) | 65 (23.5) | 188 (23.8) | 446 (38.8) |  |
| *Missing* | *42* | *42* | *88* | *172* |  |

Note: Frequencies are unweighted and percentages are weighted, except for total column, in which both are unweighted. Percentages do not sum to 100 due to rounding. NA=missing

**Table S6.** Other COVID-19 vaccine mandate attitudes of participants in the CANDOUR II study (Brazil) by early life COVID-19 vaccine mandate attitude

| **Early life COVID-19 vaccine mandate attitude** | **Disagree**  **n=353** | **Neutral**  **n=229** | **Agree**  **n=739** | **Total**  **N=1,321** | **P-value** |
| --- | --- | --- | --- | --- | --- |
|  | Unweighted frequency **(weighted %)** | Unweighted frequency **(weighted %)** | Unweighted frequency **(weighted %)** | Unweighted frequency **(unweighted %)** |  |
| **Schoolchild COVID-19 vaccine mandate attitude** |  |  |  |  |  |
| Disagree | 234 (65.6) | 36 (13.6) | 34 (1.4) | 304 (23.6) |  |
| Neutral | 44 (27.1) | 107 (48.8) | 52 (14.9) | 203 (15.7) | <0.001 |
| Agree | 66 (7.3) | 81 (37.6) | 636 (83.6) | 783 (60.7) |  |
| *Missing* | *9* | *5* | *17* | *31* |  |
| **Governmental COVID-19 vaccine mandate attitude** |  |  |  |  |  |
| Disagree | 254 (67.9) | 25 (2.8) | 51 (7.9) | 330 (25.5) |  |
| Neutral | 41 (16.2) | 108 (57.3) | 47 (7.9) | 196 (15.1) | <0.001 |
| Agree | 57 (16.0) | 92 (39.9) | 620 (84.2) | 769 (59.4) |  |
| *Missing* | *1* | *4* | *21* | *26* |  |
| **COVID-19 vaccination should be a personal choice** |  |  |  |  |  |
| Disagree | 123 (43.4) | 53 (7.2) | 273 (19.8) | 449 (34.7) |  |
| Neutral | 61 (28.8) | 116 (68.9) | 76 (9.6) | 253 (19.5) | <0.001 |
| Agree | 164 (27.8) | 57 (23.9) | 372 (70.6) | 593 (45.8) |  |
| *Missing* | *5* | *3* | *18* | *26* |  |

Notes: Frequencies are unweighted and percentages are weighted, except for total column, in which both are unweighted.

Percentages do not sum to 100 due to rounding

**CANADA**

**Table S7.** Sociodemographic characteristics of participants in the CANDOUR II study (Canada) by early life COVID-19 vaccine mandate attitude

| **Early life COVID-19 vaccine mandate attitude** | **Disagree**  **n=415** | **Neutral**  **n=264** | **Agree**  **n=455** | **Total**  **N=1,134** | **P-value** |
| --- | --- | --- | --- | --- | --- |
|  | Unweighted frequency **(weighted %)** | Unweighted frequency **(weighted %)** | Unweighted frequency **(weighted %)** | Unweighted frequency **(unweighted %)** |  |
| **Age (years)** |  |  |  |  | 0.03 |
| 18-24 | 35 (24.0) | 35 (28.8) | 41 (29.2) | 111 (9.8) |  |
| 25-34 | 99 (48.8) | 54 (11.5) | 84 (15.8) | 237 (20.9) |  |
| 35-44 | 70 (5.0) | 55 (11.6) | 80 (15.3) | 205 (18.1) |  |
| 45-54 | 52 (10.9) | 46 (9.9) | 69 (14.7) | 167 (14.7) |  |
| 55-64 | 84 (5.8) | 33 (7.1) | 97 (17.4) | 214 (18.9) |  |
| 65 and over | 75 (5.6) | 41 (31.0) | 84 (7.6) | 200 (17.6) |  |
| **Gender** |  |  |  |  | 0.67 |
| Woman | 223 (57.6) | 127 (48.5) | 220 (35.8) | 570 (50.3) |  |
| Man | 190 (28.4) | 136 (30.1) | 233 (46.9) | 559 (49.3) |  |
| Other/Prefer not to say | 2 (14.0) | 1 (21.4) | 2 (17.3) | 5 (0.4) |  |
| **Marital status** |  |  |  |  | 0.11 |
| Single | 187 (52.9) | 119 (46.9) | 184 (76.3) | 490 (43.6) |  |
| Not single | 221 (47.1) | 143 (53.1) | 270 (23.7) | 634 (56.4) |  |
| *Missing* | *7* | *2* | *1* | *10* |  |
| **Number of children** |  |  |  |  | 0.07 |
| 0 | 292 (73.9) | 194 (85.5) | 316 (88.0) | 802 (71.4) |  |
| 1 | 54 (13.0) | 41 (8.8) | 72 (6.1) | 167 (14.9) |  |
| 2 | 42 (3.3) | 22 (4.8) | 49 (4.3) | 113 (10.1) |  |
| 3 and over | 21 (9.8) | 3 (0.9) | 17 (1.6) | 41 (3.7) |  |
| *Missing* | *6* | *4* | *1* | *11* |  |
| **Education level** |  |  |  |  | 0.61 |
| Less than primary completed | 25 (6.3) | 8 (4.7) | 11 (3.1) | 44 (3.9) |  |
| Primary completed | 0 (0.0) | 0 (0.0) | 0 (0.0) | 0 (0.0) |  |
| Secondary completed | 170 (38.1) | 94 (24.0) | 157 (33.2) | 421 (37.5) |  |
| University completed | 215 (55.6) | 161 (71.3) | 283 (63.7) | 659 (58.6) |  |
| *Missing* | *5* | *1* | *4* | *10* |  |
| **Employment status** |  |  |  |  | 0.07 |
| Employed | 258 (74.4) | 176 (57.9) | 289 (41.9) | 723 (63.8) |  |
| Not employed | 157 (25.6) | 88 (42.1) | 166 (58.1) | 411 (36.2) |  |
| **Quintiles for PPP-adjusted equivalised gross annual income** |  |  |  |  | 0.07 |
| Quintile 1 (low) | 92 (41.6) | 53 (12.8) | 92 (38.2) | 237 (22.3) |  |
| Quintile 2 | 75 (23.3) | 49 (11.1) | 93 (9.3) | 217 (20.4) |  |
| Quintile 3 | 76 (14.7) | 42 (53.5) | 73 (26.3) | 191 (18.0) |  |
| Quintile 4 | 70 (5.5) | 45 (10.6) | 81 (17.3) | 196 (18.5) |  |
| Quintile 5 (high) | 74 (14.9) | 54 (12.0) | 93 (8.9) | 221 (20.8) |  |
| *Missing* | *28* | *21* | *23* | *72* |  |

Notes: Frequencies are unweighted and percentages are weighted, except for total column, in which both are unweighted.

Percentages do not sum to 100 due to rounding

| **Table S8.** Personal COVID-19 experience, health risk attitude, and political ideology of participants in the CANDOUR II study (Canada) by early life COVID-19 vaccine mandate attitude | | | | | |
| --- | --- | --- | --- | --- | --- |
| **Early life COVID-19 vaccine mandate attitude** | **Disagree**  **n=415** | **Neutral**  **n=264** | **Agree**  **n=455** | **Total**  **N=1,134** | **P-value** |
| **Number of participants** | Unweighted frequency **(weighted %)** | Unweighted frequency **(weighted %)** | Unweighted frequency **(weighted %)** | Unweighted frequency **(unweighted %)** |  |
| **COVID-19 vaccination status** |  |  |  |  | <0.001 |
| Vaccinated | 311 (81.8) | 234 (98.3) | 427 (99.8) | 972 (92.2) |  |
| Waiting for vaccination | 3 (0.3) | 1 (0.2) | 0 (0.0) | 4 (0.4) |  |
| Declined | 63 (17.4) | 6 (1.3) | 2 (1.9) | 71 (6.7) |  |
| Prefer not to say | 6 (0.5) | 1 (0.2) | 0 (0.0) | 7 (0.7) |  |
| *Missing* | *32* | *22* | *26* | *80* |  |
| **Side Effects** |  |  |  |  | 0.08 |
| No side effects | 234 (66.2) | 172 (85.0) | 311 (87.4) | 717 (73.8) |  |
| Side effects | 77 (33.8) | 62 (15.0) | 116 (12.6) | 255 (26.2) |  |
| Not vaccinated | 72 | 8 | 2 | 82 |  |
| *Missing* | *32* | *22* | *26* | *80* |  |
| **Reasons for Vaccinating** |  |  |  |  |  |
| To protect myself | 227 (89.3) | 209 (94.0) | 401 (75.8) | 837 (86.1) | 0.10 |
| To protect my family | 213 (87.9) | 192 (67.2) | 372 (93.9) | 777 (79.9) | 0.03 |
| To protect the public | 157 (68.4) | 138 (54.7) | 289 (53.2) | 584 (60.1) | 0.63 |
| To travel and visit people/places | 168 (69.8) | 134 (76.1) | 250 (37.9) | 552 (56.8) | 0.03 |
| Because everyone else will | 9 (5.0) | 15 (0.7) | 43 (6.5) | 67 (6.9) | 0.52 |
| Recommended by friends/family | 21 (26.9) | 25 (6.1) | 70 (7.6) | 116 (11.9) | 0.03 |
| Recommended by healthcare officials/professionals | 136 (65.4) | 118 (72.7) | 270 (61.8) | 524 (53.9) | 0.79 |
| Recommended by politicians | 15 (1.8) | 16 (26.5) | 66 (7.5) | 97 (10.0) | 0.02 |
| Contact with/symptoms of COVID-19 | 34 (20.6) | 20 (5.0) | 63 (7.0) | 117 (12.0) | 0.21 |
| Work/school requirement | 64 (19.8) | 36 (8.1) | 77 (7.7) | 177 (18.2) | 0.17 |
| Other reason(s) | 19 (2.2) | 3 (1.0) | 4 (0.4) | 26 (2.7) | 0.02 |
| **Personal health risk attitude** |  |  |  |  | 0.43 |
| Unwilling | 247 (56.9) | 124 (48.7) | 260 (53.7) | 631 (56.7) |  |
| Neutral | 130 (25.2) | 107 (44.9) | 112 (39.5) | 349 (31.4) |  |
| Willing | 33 (17.9) | 28 (6.4) | 72 (6.8) | 133 (11.9) |  |
| *Missing* | *5* | *5* | *11* | *21* |  |
| **Does participant know anyone who died of COVID-19?** |  |  |  |  | 0.06 |
| Yes | 84 (15.5) | 60 (56.9) | 109 (29.6) | 253 (23.2) |  |
| No | 315 (84.5) | 194 (43.1) | 330 (70.4) | 839 (76.8) |  |
| *Missing* |  |  |  |  |  |
| **Ideology** |  |  |  |  | 0.05 |
| Left | 75 (5.3) | 48 (10.6) | 122 (28.1) | 245 (21.6) |  |
| Centre | 228 (59.0) | 178 (60.3) | 221 (28.2) | 627 (55.3) |  |
| Right | 112 (35.6) | 38 (29.1) | 112 (43.8) | 262 (23.1) |  |
| *Missing* |  |  |  |  |  |

Note: Frequencies are unweighted and percentages are weighted, except for total column, in which both are unweighted. Percentages do not sum to 100 due to rounding

**Table S9.** Other COVID-19 vaccine mandate attitudes of participants in the CANDOUR II study (Canada) by early life COVID-19 vaccine mandate attitude

| **Early life COVID-19 vaccine mandate attitude** | **Disagree**  **n=415** | **Neutral**  **n=264** | **Agree**  **n=455** | **Total**  **N=1,134** | **P-value** |
| --- | --- | --- | --- | --- | --- |
|  | Unweighted frequency **(weighted %)** | Unweighted frequency **(weighted %)** | Unweighted frequency **(weighted %)** | Unweighted frequency **(unweighted %)** |  |
| **Schoolchild COVID-19 vaccine mandate attitude** |  |  |  |  |  |
| Disagree | 255 (75.2) | 22 (4.9) | 1 (0.1) | 278 (25.1) |  |
| Neutral | 62 (11.9) | 113 (46.4) | 44 (12.4) | 219 (19.8) | <0.001 |
| Agree | 83 (12.9) | 124 (48.7) | 403 (87.5) | 610 (55.1) |  |
| *Missing* | *15* | *5* | *7* | *27* |  |
| **Governmental COVID-19 vaccine mandate attitude** |  |  |  |  |  |
| Disagree | 324 (93.6) | 39 (8.2) | 20 (1.8) | 383 (34.2) |  |
| Neutral | 39 (2.9) | 130 (49.9) | 59 (30.9) | 228 (20.4) | <0.001 |
| Agree | 46 (3.4) | 93 (41.9) | 370 (67.3) | 509 (45.4) |  |
| *Missing* | *6* | *2* | *6* | *14* |  |
| **COVID-19 vaccination should be a personal choice** |  |  |  |  |  |
| Disagree | 101 (7.2) | 70 (15.0) | 199 (48.3) | 370 (33.1) | <0.001 |
| Neutral | 67 (11.7) | 122 (48.7) | 75 (7.4) | 264 (23.6) |  |
| Agree | 246 (81.2) | 70 (36.2) | 167 (44.3) | 483 (43.2) |  |
| *Missing* | *1* | *2* | *14* | *17* |  |

Notes: Frequencies are unweighted and percentages are weighted, except for total column, in which both are unweighted.

Percentages do not sum to 100 due to rounding

**CHILE**

**Table S10.** Sociodemographic characteristics of participants in the CANDOUR II study (Chile) by early life COVID-19 vaccine mandate attitude

| **Early life COVID-19 vaccine mandate attitude** | **Disagree**  **n=538** | **Neutral**  **n=216** | **Agree**  **n=484** | **Total**  **N=1,238** | **P-value** |
| --- | --- | --- | --- | --- | --- |
|  | Unweighted frequency **(weighted %)** | Unweighted frequency **(weighted %)** | Unweighted frequency **(weighted %)** | Unweighted frequency **(unweighted %)** |  |
| **Age (years)** |  |  |  |  | 0.15 |
| 18-24 | 76 (15.0) | 40 (12.3) | 91 (31.8) | 207 (16.7) |  |
| 25-34 | 164 (49.9) | 61 (20.6) | 112 (33.1) | 337 (27.2) |  |
| 35-44 | 120 (19.6) | 40 (24.8) | 87 (10.4) | 247 (20.0) |  |
| 45-54 | 91 (8.4) | 35 (11.5) | 88 (14.9) | 214 (17.3) |  |
| 55-64 | 64 (5.5) | 27 (19.8) | 78 (7.0) | 169 (13.7) |  |
| 65 and over | 23 (1.7) | 13 (11.0) | 28 (2.8) | 64 (5.2) |  |
| **Gender** |  |  |  |  | 0.01 |
| Woman | 321 (36.2) | 125 (45.1) | 240 (23.3) | 686 (55.4) |  |
| Man | 212 (17.7) | 91 (54.9) | 235 (15.3) | 538 (43.5) |  |
| Other/Prefer not to say | 5 (46.1) | 0 (0.0) | 9 (61.4) | 14 (1.1) |  |
| **Marital status** |  |  |  |  | 0.13 |
| Single | 277 (66.6) | 115 (44.6) | 259 (76.9) | 651 (53.2) |  |
| Not single | 252 (33.4) | 99 (55.4) | 221 (23.1) | 572 (46.8) |  |
| *Missing* | *9* | *2* | *4* | *15* |  |
| **Number of children** |  |  |  |  | 0.002 |
| 0 | 285 (57.7) | 122 (54.2) | 315 (87.5) | 722 (58.8) |  |
| 1 | 125 (21.5) | 51 (31.6) | 86 (5.1) | 262 (21.4) |  |
| 2 | 87 (16.3) | 27 (8.3) | 58 (6.0) | 172 (14.0) |  |
| 3 and over | 34 (4.5) | 15 (5.9) | 22 (1.4) | 71 (5.8) |  |
| *Missing* | *7* | *1* | *3* | *11* |  |
| **Education level** |  |  |  |  | 0.16 |
| Less than primary completed | 0 (0.0) | 0 (0.0) | 1 (1.1) | 1 (0.1) |  |
| Primary completed | 3 (4.6) | 5 (30.5) | 6 (7.1) | 14 (1.1) |  |
| Secondary completed | 227 (57.9) | 91 (42.6) | 169 (47.2) | 487 (39.4) |  |
| University completed | 307 (37.5) | 120 (26.9) | 306 (44.7) | 733 (59.4) |  |
| *Missing* | *1* | *0* | *2* | *3* |  |
| **Employment status** |  |  |  |  | 0.50 |
| Employed | 351 (71.4) | 131 (56.5) | 310 (60.9) | 792 (64.0) |  |
| Not employed | 187 (28.6) | 85 (43.5) | 174 (39.1) | 446 (36.0) |  |
| **Quintiles for PPP-adjusted equivalised gross annual income** |  |  |  |  | 0.47 |
| Quintile 1 (low) | 123 (27.3) | 52 (28.9) | 81 (17.1) | 256 (22.0) |  |
| Quintile 2 | 91 (21.8) | 46 (35.7) | 99 (17.1) | 236 (20.3) |  |
| Quintile 3 | 92 (22.4) | 50 (17.3) | 90 (13.5) | 232 (19.9) |  |
| Quintile 4 | 114 (22.2) | 28 (9.2) | 94 (39.0) | 236 (20.3) |  |
| Quintile 5 (high) | 81 (6.3) | 32 (8.9) | 91 (13.4) | 204 (17.5) |  |
| *Missing* | *37* | *8* | *29* | *74* |  |

Notes: Frequencies are unweighted and percentages are weighted, except for total column, in which both are unweighted.

Percentages do not sum to 100 due to rounding

| **Table S11.** Personal COVID-19 experience, health risk attitude, and political ideology of participants in the CANDOUR II study (Chile) by early life COVID-19 vaccine mandate attitude | | | | | |
| --- | --- | --- | --- | --- | --- |
| **Early life COVID-19 vaccine mandate attitude** | **Disagree**  **n=538** | **Neutral**  **n=216** | **Agree**  **n=484** | **Total**  **N=1,238** | **P-value** |
| **Number of participants** | Unweighted frequency **(weighted %)** | Unweighted frequency **(weighted %)** | Unweighted frequency **(weighted %)** | Unweighted frequency **(unweighted %)** |  |
| **COVID-19 vaccination status** |  |  |  |  | 0.001 |
| Vaccinated | 486 (97.6) | 208 (99.4) | 478 (99.9) | 1,172 (97.5) |  |
| Waiting for vaccination | 1 (0.1) | 1 (0.4) | 0 (0.0) | 2 (0.2) |  |
| Declined | 19 (1.8) | 1 (0.2) | 0 (0.0) | 20 (1.7) |  |
| Prefer not to say | 7 (0.6) | 0 (0.0) | 1 (0.001) | 8 (0.7) |  |
| *Missing* | *25* | *6* | *5* | *36* |  |
| **Side Effects** |  |  |  |  | 0.51 |
| No side effects | 426 (79.1) | 184 (86.9) | 385 (88.0) | 995 (84.9) |  |
| Side effects | 60 (20.9) | 24 (13.1) | 93 (12.0) | 177 (15.1) |  |
| Not vaccinated | 27 | 2 | 1 | 30 |  |
| *Missing* | *25* | *6* | *5* | *36* |  |
| **Reasons for Vaccinating** |  |  |  |  |  |
| To protect myself | 282 (66.3) | 157 (73.2) | 390 (86.0) | 829 (70.7) | 0.13 |
| To protect my family | 323 (61.6) | 165 (86.3) | 382 (72.0) | 870 (74.2) | 0.34 |
| To protect the public | 175 (46.0) | 89 (36.5) | 245 (60.6) | 509 (43.4) | 0.31 |
| To travel and visit people/places | 316 (82.9) | 116 (56.1) | 260 (55.1) | 692 (59.0) | 0.01 |
| Because everyone else will | 10 (4.1) | 1 (0.0002) | 17 (0.5) | 28 (2.4) | <0.001 |
| Recommended by friends/family | 18 (22.5) | 11 (10.2) | 30 (1.7) | 59 (5.0) | 0.001 |
| Recommended by healthcare officials/professionals | 122 (21.2) | 66 (36.5) | 188 (23.2) | 376 (32.1) | 0.63 |
| Recommended by politicians | 3 (0.3) | 2 (1.0) | 12 (1.0) | 17 (1.5) | 0.49 |
| Contact with/symptoms of COVID-19 | 70 (39.3) | 30 (9.5) | 103 (5.4) | 203 (17.3) | <0.001 |
| Work/school requirement | 155 (45.5) | 52 (26.6) | 121 (13.0) | 328 (28.0) | 0.02 |
| Other reason(s) | 12 (1.0) | 1 (0.2) | 5 (8.1) | 18 (1.5) | 0.002 |
| **Personal health risk attitude** |  |  |  |  | 0.88 |
| Unwilling | 348 (54.2) | 115 (46.8) | 300 (55.7) | 763 (62.5) |  |
| Neutral | 128 (29.5) | 71 (39.5) | 119 (34.1) | 318 (26.0) |  |
| Willing | 53 (16.3) | 26 (13.7) | 61 (10.2) | 140 (11.5) |  |
| *Missing* | *9* | *4* | *4* | *17* |  |
| **Does participant know anyone who died of COVID-19?** |  |  |  |  | 0.14 |
| Yes | 239 (33.6) | 122 (67.7) | 245 (52.6) | 606 (49.9) |  |
| No | 283 (66.4) | 89 (32.3) | 236 (47.4) | 608 (50.1) |  |
| *Missing* | *16* | *5* | *3* | *24* |  |
| **Ideology** |  |  |  |  | 0.14 |
| Left | 174 (36.4) | 75 (32.4) | 168 (59.3) | 417 (35.5) |  |
| Centre | 256 (57.1) | 105 (52.4) | 205 (35.3) | 566 (48.3) |  |
| Right | 76 (6.4) | 30 (15.2) | 84 (5.4) | 190 (16.2) |  |
| *Missing* | *32* | *6* | *27* | *65* |  |

Note: Frequencies are unweighted and percentages are weighted, except for total column, in which both are unweighted. Percentages do not sum to 100 due to rounding

**Table S12.** Other COVID-19 vaccine mandate attitudes of participants in the CANDOUR II study (Chile) by early life COVID-19 vaccine mandate attitude

| **Early life COVID-19 vaccine mandate attitude** | **Disagree**  **n=538** | **Neutral**  **n=216** | **Agree**  **n=484** | **Total**  **N=1,238** | **P-value** |
| --- | --- | --- | --- | --- | --- |
|  | Unweighted frequency **(weighted %)** | Unweighted frequency **(weighted %)** | Unweighted frequency **(weighted %)** | Unweighted frequency **(unweighted %)** |  |
| **Schoolchild COVID-19 vaccine mandate attitude** |  |  |  |  |  |
| Disagree | 277 (51.7) | 13 (4.7) | 13 (0.7) | 303 (24.9) |  |
| Neutral | 97 (27.3) | 76 (37.2) | 31 (1.9) | 204 (16.8) | <0.001 |
| Agree | 151 (21.0) | 124 (58.2) | 435 (97.4) | 710 (58.3) |  |
| *Missing* | *13* | *3* | *5* | *21* |  |
| **Governmental COVID-19 vaccine mandate attitude** |  |  |  |  |  |
| Disagree | 354 (60.2) | 33 (11.3) | 46 (9.2) | 433 (35.6) |  |
| Neutral | 73 (23.3) | 80 (32.5) | 52 (17.5) | 205 (16.8) | 0.001 |
| Agree | 103 (16.5) | 100 (56.2) | 377 (73.2) | 580 (47.6) |  |
| *Missing* | *8* | *3* | *9* | *20* |  |
| **COVID-19 vaccination should be a personal choice** |  |  |  |  |  |
| Disagree | 150 (30.5) | 67 (31.4) | 223 (55.1) | 440 (36.0) |  |
| Neutral | 76 (16.6) | 77 (35.7) | 80 (19.9) | 233 (19.1) | 0.15 |
| Agree | 306 (52.9) | 71 (32.9) | 172 (25.0) | 549 (44.9) |  |
| *Missing* | *6* | *1* | *9* | *16* |  |

Notes: Frequencies are unweighted and percentages are weighted, except for total column, in which both are unweighted.

Percentages do not sum to 100 due to rounding

**CHINA**

**Table S13.** Sociodemographic characteristics of participants in the CANDOUR II study (China) by early life COVID-19 vaccine mandate attitude

| **Early life COVID-19 vaccine mandate attitude** | **Disagree**  **n=208** | **Neutral**  **n=303** | **Agree**  **n=779** | **Total**  **N=1,290** | **P-value** |
| --- | --- | --- | --- | --- | --- |
|  | Unweighted frequency **(weighted %)** | Unweighted frequency **(weighted %)** | Unweighted frequency **(weighted %)** | Unweighted frequency **(unweighted %)** |  |
| **Age (years)** |  |  |  |  | 0.33 |
| 18-24 | 6 (3.2) | 21 (17.7) | 76 (22.6) | 103 (8.0) |  |
| 25-34 | 48 (19.5) | 67 (18.4) | 118 (15.2) | 233 (18.1) |  |
| 35-44 | 61 (25.9) | 68 (18.4) | 115 (15.7) | 244 (18.9) |  |
| 45-54 | 35 (22.3) | 64 (28.2) | 146 (25.4) | 245 (19.0) |  |
| 55-64 | 42 (22.5) | 68 (15.1) | 249 (13.60) | 359 (27.8) |  |
| 65 and over | 16 (6.6) | 15 (2.3) | 75 (7.4) | 106 (8.2) |  |
| **Gender** |  |  |  |  | 0.07 |
| Woman | 100 (27.3) | 147 (54.9) | 381 (50.0) | 628 (48.7) |  |
| Man | 108 (72.7) | 155 (43.2) | 397 (49.2) | 660 (51.2) |  |
| Other/Prefer not to say | 0 (0.0) | 1 (1.9) | 1 (0.1) | 2 (0.2) |  |
| **Marital status** |  |  |  |  | 0.15 |
| Single | 27 (12.7) | 52 (26.4) | 131 (28.8) | 210 (16.4) |  |
| Not single | 180 (87.3) | 249 (73.6) | 642 (71.2) | 1,071 (83.6) |  |
| *Missing* | *1* | *2* | *6* | *9* |  |
| **Number of children** |  |  |  |  | 0.09 |
| 0 | 72 (26.4) | 118 (51.5) | 300 (49.1) | 490 (38.3) |  |
| 1 | 120 (64.0) | 152 (42.0) | 368 (40.9) | 640 (50.0) |  |
| 2 | 16 (9.5) | 30 (6.5) | 87 (7.6) | 133 (10.4) |  |
| 3 and over | 0 (0.0) | 1 (0.0003) | 17 (2.4) | 18 (1.4) |  |
| *Missing* | *0* | *2* | *7* | *9* |  |
| **Education level** |  |  |  |  | 0.87 |
| Less than primary completed | 2 (6.3) | 3 (5.9) | 10 (8.1) | 15 (1.2) |  |
| Primary completed | 66 (3.2) | 98 (8.8) | 375 (8.4) | 539 (42.1) |  |
| Secondary completed | 43 (85.5) | 65 (79.7) | 143 (77.5) | 251 (19.6) |  |
| University completed | 96 (5.0) | 136 (5.6) | 243 (6.0) | 475 (37.1) |  |
| *Missing* | *1* | *1* | *8* | *10* |  |
| **Employment status** |  |  |  |  | 0.26 |
| Employed | 160 (62.2) | 230 (77.2) | 565 (67.2) | 955 (74.0) |  |
| Not employed | 48 (37.8) | 73 (22.8) | 214 (32.8) | 335 (26.0) |  |
| **Quintiles for PPP-adjusted equivalised gross annual income** |  |  |  |  | 0.83 |
| Quintile 1 (low) | 37 (31.4) | 59 (28.7) | 194 (33.3) | 290 (22.7) |  |
| Quintile 2 | 33 (13.3) | 51 (28.1) | 131 (20.4) | 215 (16.8) |  |
| Quintile 3 | 45 (28.5) | 58 (22.0) | 154 (21.9) | 257 (20.1) |  |
| Quintile 4 | 47 (19.5) | 60 (15.8) | 152 (15.0) | 259 (20.3) |  |
| Quintile 5 (high) | 46 (7.4) | 71 (5.4) | 141 (9.5) | 258 (20.2) |  |
| *Missing* | *0* | *4* | *7* | *11* |  |

Notes: Frequencies are unweighted and percentages are weighted, except for total column, in which both are unweighted.

Percentages do not sum to 100 due to rounding

| **Table S14.** Personal COVID-19 experience, health risk attitude, and political ideology of participants in the CANDOUR II study (China) by early life COVID-19 vaccine mandate attitude | | | | | |
| --- | --- | --- | --- | --- | --- |
| **Early life COVID-19 vaccine mandate attitude** | **Disagree**  **n=208** | **Neutral**  **n=303** | **Agree**  **n=779** | **Total**  **N=1,290** | **P-value** |
| **Number of participants** | Unweighted frequency **(weighted %)** | Unweighted frequency **(weighted %)** | Unweighted frequency **(weighted %)** | Unweighted frequency **(unweighted %)** |  |
| **COVID-19 vaccination status** |  |  |  |  | 0.002 |
| Vaccinated | 178 (83.0) | 248 (96.9) | 647 (99.1) | 1,073 (97.8) |  |
| Waiting for vaccination | 2 (3.4) | 0 (0.0) | 0 (0.0) | 2 (0.2) |  |
| Declined | 10 (13.7) | 5 (2.3) | 4 (0.9) | 19 (1.7) |  |
| Prefer not to say | 0 (0.0) | 3 (0.1) | 0 (0.0) | 3 (0.3) |  |
| *Missing* | *18* | *47* | *128* | *193* |  |
| **Side Effects** |  |  |  |  | 0.69 |
| No side effects | 76 (31.3) | 64 (30.4) | 125 (25.0) | 265 (24.7) |  |
| Side effects | 102 (68.7) | 184 (69.6) | 522 (75.0) | 808 (75.3) |  |
| Not vaccinated | 12 | 8 | 4 | 24 |  |
| *Missing* | *18* | *47* | *128* | *193* |  |
| **Reasons for Vaccinating** |  |  |  |  |  |
| To protect myself | 134 (86.2) | 212 (85.6) | 567 (91.1) | 913 (85.1) | 0.51 |
| To protect my family | 121 (81.6) | 187 (65.0) | 524 (82.7) | 832 (77.5) | 0.04 |
| To protect the public | 56 (38.7) | 81 (20.1) | 284 (45.1) | 421 (39.2) | 0.01 |
| To travel and visit people/places | 34 (13.6) | 74 (24.3) | 226 (35.8) | 334 (31.1) | 0.05 |
| Because everyone else will | 47 (30.4) | 74 (86.4) | 247 (27.8) | 368 (34.3) | 0.08 |
| Recommended by friends/family | 17 (8.9) | 30 (9.7) | 91 (20.3) | 138 (12.9) | 0.13 |
| Recommended by healthcare officials/professionals | 55 (26.5) | 69 (12.9) | 205 (34.6) | 329 (30.7) | 0.2 |
| Recommended by politicians | 37 (13.7) | 42 (14.7) | 128 (25.9) | 207 (19.3) | 0.16 |
| Contact with/symptoms of COVID-19 | 2 (0.0005) | 8 (1.7) | 26 (3.8) | 36 (3.4) | <0.001 |
| Work/school requirement | 58 (18.5) | 54 (30.4) | 167 (36.1) | 279 (26.0) | 0.20 |
| Other reason(s) | 2 (0.1) | 0 (0.0) | 2 (0.1) | 4 (0.4) | 0.48 |
| **Personal health risk attitude** |  |  |  |  | 0.004 |
| Unwilling | 162 (71.3) | 138 (47.8) | 373 (60.8) | 673 (53.2) |  |
| Neutral | 32 (22.4) | 110 (27.7) | 94 (10.7) | 236 (18.7) |  |
| Willing | 10 (6.4) | 50 (24.5) | 296 (28.5) | 356 (28.1) |  |
| *Missing* | *4* | *5* | *16* | *25* |  |
| **Does participant know anyone who died of COVID-19?** |  |  |  |  | 0.82 |
| Yes | 6 (3.2) | 15 (6.3) | 53 (5.3) | 74 (5.8) |  |
| No | 200 (97.8) | 282 (93.7) | 718 (94.7) | 1,200 (94.2) |  |
| *Missing* | *2* | *6* | *8* | *16* |  |
| **Ideology** |  |  |  |  | NA |
| Left | NA | NA | NA | NA |  |
| Centre |  |  |  |  |  |
| Right |  |  |  |  |  |
| *Missing* |  |  |  |  |  |

Note: Frequencies are unweighted and percentages are weighted, except for total column, in which both are xunweighted. Percentages do not sum to 100 due to rounding

NA: This question was not included for participants from China

**Table S15.** Other COVID-19 vaccine mandate attitudes of participants in the CANDOUR II study (China) by early life COVID-19 vaccine mandate attitude

| **Early life COVID-19 vaccine mandate attitude** | **Disagree**  **n=208** | **Neutral**  **n=303** | **Agree**  **n=779** | **Total**  **N=1,290** | **P-value** |
| --- | --- | --- | --- | --- | --- |
|  | Unweighted frequency **(weighted %)** | Unweighted frequency **(weighted %)** | Unweighted frequency **(weighted %)** | Unweighted frequency **(unweighted %)** |  |
| **Schoolchild COVID-19 vaccine mandate attitude** |  |  |  |  |  |
| Disagree | 108 (65.5) | 28 (12.2) | 14 (0.9) | 150 (11.8) |  |
| Neutral | 57 (30.0) | 133 (39.9) | 99 (12.3) | 289 (22.8) | <0.001 |
| Agree | 41 (4.5) | 136 (47.9) | 654 (86.8) | 831 (65.4) |  |
| *Missing* | *2* | *6* | *12* | *20* |  |
| **Governmental COVID-19 vaccine mandate attitude** |  |  |  |  |  |
| Disagree | 129 (76.3) | 39 (16.3) | 43 (5.8) | 211 (16.4) |  |
| Neutral | 41 (13.3) | 149 (49.9) | 90 (12.2) | 280 (21.8) | <0.001 |
| Agree | 38 (10.4) | 113 (33.8) | 641 (82.1) | 792 (61.7) |  |
| *Missing* | *0* | *2* | *5* | *7* |  |
| **COVID-19 vaccination should be a personal choice** |  |  |  |  |  |
| Disagree | 50 (25.8) | 52 (20.7) | 185 (29.3) | 287 (22.4) |  |
| Neutral | 32 (10.2) | 138 (49.8) | 107 (15.1) | 277 (21.6) | <0.001 |
| Agree | 126 (64.0) | 109 (29.5) | 483 (55.6) | 718 (56.0) |  |
| *Missing* | *0* | *4* | *4* | *8* |  |

Notes: Frequencies are unweighted and percentages are weighted, except for total column, in which both are unweighted.

Percentages do not sum to 100 due to rounding

**COLOMBIA**

**Table S16.** Sociodemographic characteristics of participants in the CANDOUR II study (Colombia) by early life COVID-19 vaccine mandate attitude

| **Early life COVID-19 vaccine mandate attitude** | **Disagree**  **n=428** | **Neutral**  **n=270** | **Agree**  **n=507** | **Total**  **N=1,205** | **P-value** |
| --- | --- | --- | --- | --- | --- |
|  | Unweighted frequency **(weighted %)** | Unweighted frequency **(weighted %)** | Unweighted frequency **(weighted %)** | Unweighted frequency **(unweighted %)** |  |
| **Age (years)** |  |  |  |  | 0.001 |
| 18-24 | 53 (29.6) | 42 (6.3) | 42 (3.0) | 137 (11.4) |  |
| 25-34 | 142 (23.7) | 74 (11.0) | 134 (17.7) | 350 (29.0) |  |
| 35-44 | 93 (13.4) | 52 (6.6) | 105 (16.2) | 250 (20.7) |  |
| 45-54 | 66 (10.5) | 43 (19.6) | 92 (23.5) | 201 (16.7) |  |
| 55-64 | 51 (7.2) | 40 (51.8) | 86 (32.5) | 177 (14.7) |  |
| 65 and over | 23 (15.6) | 19 (4.7) | 48 (7.1) | 90 (7.5) |  |
| **Gender** |  |  |  |  | 0.29 |
| Woman | 236 (53.7) | 125 (51.2) | 261 (37.2) | 622 (51.6) |  |
| Man | 192 (46.3) | 145 (48.8) | 244 (45.4) | 581 (48.2) |  |
| Other/Prefer not to say | 0 (0.0) | 0 (0.0) | 2 (17.5) | 2 (0.2) |  |
| **Marital status** |  |  |  |  | 0.46 |
| Single | 186 (54.5) | 123 (32.0) | 207 (50.0) | 516 (43.6) |  |
| Not single | 236 (45.4) | 140 (68.0) | 291 (58.4) | 667 (56.4) |  |
| *Missing* | *6* | *7* | *9* | *22* |  |
| **Number of children** |  |  |  |  | 0.03 |
| 0 | 222 (77.1) | 148 (81.4) | 278 (53.7) | 648 (54.4) |  |
| 1 | 112 (13.6) | 68 (10.0) | 125 (19.1) | 305 (25.6) |  |
| 2 | 69 (7.1) | 36 (6.6) | 84 (25.2) | 189 (15.9) |  |
| 3 and over | 18 (2.3) | 16 (2.0) | 15 (2.0) | 49 (4.1) |  |
| *Missing* | *7* | *2* | *5* | *14* |  |
| **Education level** |  |  |  |  | 0.08 |
| Less than primary completed | 12 (14.8) | 6 (23.4) | 7 (6.9) | 25 (2.1) |  |
| Primary completed | 23 (29.5) | 10 (11.0) | 14 (12.8) | 47 (3.9) |  |
| Secondary completed | 195 (34.8) | 143 (55.6) | 226 (49.5) | 564 (47.2) |  |
| University completed | 194 (20.9) | 110 (10.0) | 256 (30.8) | 560 (46.8) |  |
| *Missing* | *4* | *1* | *4* | *9* |  |
| **Employment status** |  |  |  |  | 0.45 |
| Employed | 290 (65.8) | 176 (67.2) | 324 (48.8) | 790 (65.6) |  |
| Not employed | 138 (34.2) | 94 (32.8) | 183 (51.2) | 415 (34.4) |  |
| **Quintiles for PPP-adjusted equivalised gross annual income** |  |  |  |  | 0.87 |
| Quintile 1 (low) | 71 (13.6) | 54 (21.1) | 96 (25.9) | 221 (20.4) |  |
| Quintile 2 | 89 (14.1) | 42 (10.9) | 83 (9.8) | 214 (19.8) |  |
| Quintile 3 | 81 (28.7) | 49 (30.5) | 87 (19.4) | 217 (20.1) |  |
| Quintile 4 | 69 (9.5) | 55 (7.8) | 96 (16.9) | 220 (20.4) |  |
| Quintile 5 (high) | 78 (34.1) | 43 (29.7) | 88 (28.1) | 209 (19.3) |  |
| *Missing* | *40* | *27* | *57* | *124* |  |

Notes: Frequencies are unweighted and percentages are weighted, except for total column, in which both are unweighted.

Percentages do not sum to 100 due to rounding

| **Table S17.** Personal COVID-19 experience, health risk attitude, and political ideology of participants in the CANDOUR II study (Colombia) by early life COVID-19 vaccine mandate attitude | | | | | |
| --- | --- | --- | --- | --- | --- |
| **Early life COVID-19 vaccine mandate attitude** | **Disagree**  **n=428** | **Neutral**  **n=270** | **Agree**  **n=507** | **Total**  **N=1,205** | **P-value** |
| **Number of participants** | Unweighted frequency **(weighted)** | Unweighted frequency **(weighted** | Unweighted frequency **(weighted)** | Unweighted frequency **(unweighted)** |  |
| **COVID-19 vaccination status** |  |  |  |  | <0.001 |
| Vaccinated | 365 (81.5) | 250 (99.2) | 487 (99.9) | 1,102 (96.7) |  |
| Waiting for vaccination | 1 (0.004) | 2 (0.4) | 0 (0.0) | 3 (0.3) |  |
| Declined | 26 (18.2) | 3 (0.3) | 2 (0.1) | 31 (2.7) |  |
| Prefer not to say | 3 (0.2) | 1 (0.004) | 0 (0.0) | 4 (0.4) |  |
| *Missing* | *33* | *14* | *18* | *65* |  |
| **Side Effects** |  |  |  |  | 0.16 |
| No side effects | 299 (88.6) | 195 (87.6) | 376 (73.7) | 870 (78.9) |  |
| Side effects | 66 (11.4) | 55 (12.4) | 111 (26.3) | 232 (21.1) |  |
| Not vaccinated | 30 | 6 | 2 | 38 |  |
| *Missing* | *33* | *14* | *18* | *65* |  |
| **Reasons for Vaccinating** |  |  |  |  |  |
| To protect myself | 225 (52.0) | 193 (72.1) | 397 (83.0) | 815 (74.0) | 0.17 |
| To protect my family | 228 (67.9) | 190 (88.0) | 375 (72.4) | 793 (72.0) | 0.35 |
| To protect the public | 106 (29.0) | 89 (51.1) | 184 (22.6) | 379 (34.4) | 0.26 |
| To travel and visit people/places | 186 (54.6) | 108 (55.0) | 188 (20.9) | 482 (43.7) | 0.06 |
| Because everyone else will | 4 (0.1) | 10 (0.4) | 16 (0.5) | 30 (2.7) | 0.19 |
| Recommended by friends/family | 13 (3.1) | 15 (2.2) | 29 (2.3) | 57 (5.2) | 0.87 |
| Recommended by healthcare officials/professionals | 91 (14.8) | 67 (28.9) | 152 (12.9) | 310 (28.1) | 0.28 |
| Recommended by politicians | 2 (0.3) | 4 (2.2) | 5 (0.3) | 11 (1.0) | 0.004 |
| Contact with/symptoms of COVID-19 | 53 (10.0) | 50 (28.0) | 113 (17.1) | 216 (19.6) | 0.40 |
| Work/school requirement | 98 (13.5) | 37 (22.8) | 71 (3.9) | 206 (18.7) | 0.09 |
| Other reason(s) | 10 (1.4) | 3 (0.2) | 1 (0.004) | 14 (1.3) | <0.001 |
| **Personal health risk attitude** |  |  |  |  | 0.70 |
| Unwilling | 287 (59.6) | 134 (47.8) | 306 (52.7) | 727 (62.4) |  |
| Neutral | 85 (34.6) | 90 (31.2) | 102 (36.6) | 277 (23.8) |  |
| Willing | 43 (5.8) | 37 (21.0) | 81 (10.7) | 161 (13.8) |  |
| *Missing* | *13* | *9* | *18* | *40* |  |
| **Does participant know anyone who died of COVID-19?** |  |  |  |  | 0.62 |
| Yes | 281 (59.9) | 166 (72.4) | 383 (75.1) | 830 (70.6) |  |
| No | 138 (40.1) | 95 (27.6) | 113 (24.9) | 346 (29.4) |  |
| *Missing* | *9* | *9* | *11* | *29* |  |
| **Ideology** |  |  |  |  | 0.02 |
| Left | 152 (53.8) | 68 (8.4) | 116 (28.7) | 336 (29.6) |  |
| Centre | 171 (34.9) | 129 (49.6) | 212 (25.9) | 512 (45.1) |  |
| Right | 78 (11.3) | 59 (42.0) | 151 (45.5) | 288 (25.4) |  |
| *Missing* | *27* | *14* | *28* | *69* |  |

Note: Frequencies are unweighted and percentages are weighted, except for total column, in which both are unweighted. Percentages do not sum to 100 due to rounding

**Table S18.** Other COVID-19 vaccine mandate attitudes of participants in the CANDOUR II study (Colombia) by early life COVID-19 vaccine mandate attitude

| **Early life COVID-19 vaccine mandate attitude** | **Disagree**  **n=428** | **Neutral**  **n=270** | **Agree**  **n=507** | **Total**  **N=1,205** | **P-value** |
| --- | --- | --- | --- | --- | --- |
|  | Unweighted frequency **(weighted %)** | Unweighted frequency **(weighted %)** | Unweighted frequency **(weighted %)** | Unweighted frequency **(unweighted %)** |  |
| **Schoolchild COVID-19 vaccine mandate attitude** |  |  |  |  |  |
| Disagree | 233 (66.5) | 23 (21.7) | 13 (1.5) | 269 (22.8) |  |
| Neutral | 69 (7.9) | 117 (41.6) | 41 (3.3) | 227 (19.3) | <0.001 |
| Agree | 111 (25.6) | 124 (36.7) | 448 (95.2) | 683 (57.9) |  |
| *Missing* | *15* | *6* | *5* | *26* |  |
| **Governmental COVID-19 vaccine mandate attitude** |  |  |  |  |  |
| Disagree | 303 (74.3) | 47 (25.3) | 33 (2.9) | 383 (32.1) |  |
| Neutral | 54 (7.4) | 119 (38.6) | 55 (12.5) | 228 (19.1) | <0.001 |
| Agree | 69 (18.4) | 100 (36.1) | 413 (84.6) | 582 (48.8) |  |
| *Missing* | *2* | *4* | *6* | *12* |  |
| **COVID-19 vaccination should be a personal choice** |  |  |  |  |  |
| Disagree | 114 (26.7) | 46 (42.7) | 165 (27.9) | 325 (27.4) |  |
| Neutral | 56 (6.9) | 115 (36.5) | 104 (26.9) | 275 (23.2) | 0.09 |
| Agree | 253 (66.4) | 104 (20.8) | 230 (45.1) | 587 (49.5) |  |
| *Missing* | *5* | *5* | *8* | *18* |  |

Notes: Frequencies are unweighted and percentages are weighted, except for total column, in which both are unweighted.

Percentages do not sum to 100 due to rounding

**FRANCE**

**Table S19.** Sociodemographic characteristics of participants in the CANDOUR II study (France) by early life COVID-19 vaccine mandate attitude

| **Early life COVID-19 vaccine mandate attitude** | **Disagree**  **n=625** | **Neutral**  **n=207** | **Agree**  **n=278** | **Total**  **N=1,110** | **P-value** |
| --- | --- | --- | --- | --- | --- |
|  | Unweighted frequency **(weighted %)** | Unweighted frequency **(weighted %)** | Unweighted frequency **(weighted %)** | Unweighted frequency **(unweighted %)** |  |
| **Age (years)** |  |  |  |  | 0.09 |
| 18-24 | 49 (18.2) | 17 (4.0) | 20 (4.0) | 86 (7.7) |  |
| 25-34 | 121 (17.8) | 34 (7.9) | 56 (22.8) | 211 (19.0) |  |
| 35-44 | 127 (24.2) | 36 (34.6) | 29 (18.8) | 192 (17.3) |  |
| 45-54 | 104 (23.3) | 33 (7.3) | 41 (35.7) | 178 (16.0) |  |
| 55-64 | 137 (9.9) | 47 (9.6) | 80 (11.5) | 264 (23.8) |  |
| 65 and over | 87 (6.7) | 40 (36.7) | 52 (7.3) | 179 (16.1) |  |
| **Gender** |  |  |  |  | 0.61 |
| Woman | 376 (59.8) | 85 (43.4) | 128 (47.6) | 589 (53.1) |  |
| Man | 248 (32.7) | 122 (56.6) | 150 (52.4) | 520 (46.8) |  |
| Other/Prefer not to say | 1 (7.6) | 0 (0.0) | 0 (0.0) | 1 (0.1) |  |
| **Marital status** |  |  |  |  | 0.73 |
| Single | 197 (32.0) | 76 (46.0) | 97 (29.2) | 370 (33.7) |  |
| Not single | 421 (68.0) | 128 (54.0) | 178 (70.8) | 727 (66.3) |  |
| *Missing* | *7* | *3* | *3* | *13* |  |
| **Number of children** |  |  |  |  | 0.002 |
| 0 | 358 (44.0) | 142 (59.1) | 188 (43.1) | 688 (62.6) |  |
| 1 | 110 (14.9) | 34 (8.2) | 58 (53.5) | 202 (18.4) |  |
| 2 | 101 (15.2) | 26 (31.7) | 22 (2.7) | 149 (13.6) |  |
| 3 and over | 48 (25.9) | 5 (0.1) | 7 (0.8) | 60 (5.5) |  |
| *Missing* | *8* | *0* | *3* | *11* |  |
| **Education level** |  |  |  |  | 0.42 |
| Less than primary completed | 2 (0.9) | 0 (0.0) | 0 (0.0) | 2 (0.2) |  |
| Primary completed | 42 (13.7) | 8 (9.3) | 17 (26.5) | 67 (6.1) |  |
| Secondary completed | 287 (39.2) | 79 (58.6) | 95 (51.0) | 461 (41.8) |  |
| University completed | 290 (46.3) | 119 (32.2) | 165 (22.6) | 574 (52.0) |  |
| *Missing* | *4* | *1* | *1* | *6* |  |
| **Employment status** |  |  |  |  | 0.22 |
| Employed | 286 (50.3) | 93 (20.2) | 136 (49.1) | 515 (46.4) |  |
| Not employed | 339 (49.7) | 114 (79.2) | 142 (50.9) | 595 (53.6) |  |
| **Quintiles for PPP-adjusted equivalised gross annual income** |  |  |  |  | 0.01 |
| Quintile 1 (low) | 122 (30.4) | 40 (63.6) | 47 (38.8) | 209 (20.4) |  |
| Quintile 2 | 129 (14.2) | 32 (6.8) | 46 (7.1) | 207 (20.2) |  |
| Quintile 3 | 123 (35.9) | 37 (10.5) | 54 (7.8) | 214 (20.9) |  |
| Quintile 4 | 162 (16.7) | 67 (14.4) | 77 (41.7) | 306 (29.9) |  |
| Quintile 5 (high) | 30 (2.9) | 22 (4.7) | 37 (4.7) | 89 (8.7) |  |
| *Missing* | *59* | *9* | *17* | *85* |  |

Notes: Frequencies are unweighted and percentages are weighted, except for total column, in which both are unweighted.

Percentages do not sum to 100 due to rounding

| **Table S20.** Personal COVID-19 experience, health risk attitude, and political ideology of participants in the CANDOUR II study (France) by early life COVID-19 vaccine mandate attitude | | | | | |
| --- | --- | --- | --- | --- | --- |
| **Early life COVID-19 vaccine mandate attitude** | **Disagree**  **n=625** | **Neutral**  **n=207** | **Agree**  **n=278** | **Total**  **N=1,110** | **P-value** |
| **Number of participants** | Unweighted frequency **(weighted %)** | Unweighted frequency **(weighted %)** | Unweighted frequency **(weighted %)** | Unweighted frequency **(unweighted %)** |  |
| **COVID-19 vaccination status** |  |  |  |  | <0.001 |
| Vaccinated | 457 (78.6) | 164 (98.7) | 246 (83.5) | 867 (89.7) |  |
| Waiting for vaccination | 1 (0.1) | 1 (0.3) | 1 (15.6) | 3 (0.3) |  |
| Declined | 82 (20.4) | 3 (1.0) | 5 (0.7) | 90 (9.3) |  |
| Prefer not to say | 5 (0.9) | 0 (0.0) | 2 (0.3) | 7 (0.7) |  |
| *Missing* | *80* | *39* | *24* | *143* |  |
| **Side Effects** |  |  |  |  | 0.23 |
| No side effects | 348 (72.9) | 109 (77.8) | 159 (48.5) | 616 (71.0) |  |
| Side effects | 109 (27.1) | 55 (22.2) | 87 (51.5) | 251 (29.0) |  |
| Not vaccinated | 88 | 4 | 8 | 100 |  |
| *Missing* | *80* | *39* | *24* | *143* |  |
| **Reasons for Vaccinating** |  |  |  |  |  |
| To protect myself | 238 (43.6) | 118 (81.8) | 193 (34.0) | 549 (63.3) | 0.04 |
| To protect my family | 261 (45.6) | 108 (78.5) | 185 (51.1) | 554 (63.9) | 0.29 |
| To protect the public | 124 (28.9) | 50 (18.9) | 110 (17.7) | 284 (32.8) | 0.50 |
| To travel and visit people/places | 200 (25.9) | 71 (64.1) | 124 (41.5) | 395 (45.6) | 0.15 |
| Because everyone else will | 10 (0.8) | 9 (0.5) | 15 (0.8) | 34 (3.9) | 0.64 |
| Recommended by friends/family | 8 (1.6) | 4 (1.3) | 15 (21.0) | 27 (3.1) | <0.001 |
| Recommended by healthcare officials/professionals | 124 (29.2) | 51 (18.4) | 109 (19.4) | 284 (32.8) | 0.55 |
| Recommended by politicians | 12 (1.7) | 10 (3.5) | 21 (3.2) | 43 (5.0) | 0.44 |
| Contact with/symptoms of COVID-19 | 48 (19.6) | 13 (4.3) | 45 (6.7) | 106 (12.2) | 0.09 |
| Work/school requirement | 92 (25.6) | 18 (7.0) | 30 (23.3) | 140 (16.1) | 0.50 |
| Other reason(s) | 27 (3.2) | 4 (3.4) | 5 (0.7) | 36 (4.2) | 0.18 |
| **Personal health risk attitude** |  |  |  |  | 0.01 |
| Unwilling | 343 (58.4) | 82 (26.9) | 138 (35.3) | 563 (52.7) |  |
| Neutral | 183 (35.3) | 94 (63.7) | 76 (25.6) | 353 (33.0) |  |
| Willing | 71 (6.4) | 26 (9.4) | 56 (39.1) | 153 (14.3) |  |
| *Missing* | *28* | *5* | *8* | *41* |  |
| **Does participant know anyone who died of COVID-19?** |  |  |  |  | 0.30 |
| Yes | 121 (31.8) | 59 (19.1) | 75 (48.9) | 255 (23.7) |  |
| No | 484 (68.2) | 141 (80.9) | 194 (51.1) | 819 (76.3) |  |
| *Missing* | *20* | *7* | *9* | *36* |  |
| **Ideology** |  |  |  |  | 0.22 |
| Left | 129 (20.4) | 34 (46.9) | 47 (8.0) | 210 (23.0) |  |
| Centre | 182 (53.0) | 101 (36.3) | 100 (54.7) | 383 (41.9) |  |
| Right | 179 (26.7) | 48 (16.8) | 93 (37.3) | 320 (35.0) |  |
| *Missing* | *135* | *24* | *38* | *197* |  |

Note: Frequencies are unweighted and percentages are weighted, except for total column, in which both are unweighted. Percentages do not sum to 100 due to rounding

**Table S21.** Other COVID-19 vaccine mandate attitudes of participants in the CANDOUR II study (France) by early life COVID-19 vaccine mandate attitude

| **Early life COVID-19 vaccine mandate attitude** | **Disagree**  **n=625** | **Neutral**  **n=207** | **Agree**  **n=278** | **Total**  **N=1,110** | **P-value** |
| --- | --- | --- | --- | --- | --- |
|  | Unweighted frequency **(weighted %)** | Unweighted frequency **(weighted %)** | Unweighted frequency **(weighted %)** | Unweighted frequency **(unweighted %)** |  |
| **Schoolchild COVID-19 vaccine mandate attitude** |  |  |  |  |  |
| Disagree | 462 (81.4) | 43 (10.6) | 45 (21.7) | 550 (51.1) |  |
| Neutral | 80 (12.9) | 93 (74.7) | 61 (25.0) | 234 (21.7) | <0.001 |
| Agree | 68 (5.7) | 63 (14.8) | 162 (53.3) | 293 (27.2) |  |
| *Missing* | *15* | *8* | *10* | *33* |  |
| **Governmental COVID-19 vaccine mandate attitude** |  |  |  |  |  |
| Disagree | 453 (64.9) | 44 (14.9) | 56 (23.3) | 553 (50.7) |  |
| Neutral | 70 (12.8) | 97 (64.2) | 39 (22.3) | 206 (18.9) | 0.01 |
| Agree | 93 (22.3) | 60 (20.9) | 178 (54.4) | 331 (30.4) |  |
| *Missing* | *9* | *6* | *5* | *20* |  |
| **COVID-19 vaccination should be a personal choice** |  |  |  |  |  |
| Disagree | 116 (26.1) | 47 (11.7) | 102 (28.0) | 265 (24.1) |  |
| Neutral | 101 (25.1) | 96 (73.5) | 52 (23.1) | 249 (22.7) | 0.07 |
| Agree | 402 (48.8) | 61 (14.8) | 121 (48.9) | 584 (53.2) |  |
| *Missing* |  |  |  |  |  |

Notes: Frequencies are unweighted and percentages are weighted, except for total column, in which both are unweighted.

Percentages do not sum to 100 due to rounding

**GHANA**

**Table S22.** Sociodemographic characteristics of participants in the CANDOUR II study (Ghana) by early life COVID-19 vaccine mandate attitude

| **Early life COVID-19 vaccine mandate attitude** | **Disagree**  **n=528** | **Neutral**  **n=245** | **Agree**  **n=452** | **Total**  **N=1,225** | **P-value** |
| --- | --- | --- | --- | --- | --- |
|  | Unweighted frequency **(weighted %)** | Unweighted frequency **(weighted %)** | Unweighted frequency **(weighted %)** | Unweighted frequency **(unweighted %)** |  |
| **Age (years)** |  |  |  |  | 0.21 |
| 18-24 | 95 (13.5) | 48 (23.9) | 97 (24.9) | 240 (19.6) |  |
| 25-34 | 259 (53.3) | 136 (58.9) | 227 (52.7) | 622 (50.8) |  |
| 35-44 | 130 (21.9) | 43 (13.1) | 94 (16.4) | 267 (21.8) |  |
| 45-54 | 41 (11.0) | 15 (1.1) | 29 (5.4) | 85 (6.9) |  |
| 55-64 | 3 (0.3) | 1 (0.2) | 4 (0.3) | 8 (0.7) |  |
| 65 and over | 0 (0.0) | 2 (2.9) | 1 (0.3) | 3 (0.2) |  |
| **Gender** |  |  |  |  | 0.41 |
| Woman | 160 (23.2) | 49 (17.1) | 124 (16.8) | 333 (27.2) |  |
| Man | 366 (72.4) | 196 (82.9) | 324 (74.9) | 886 (72.3) |  |
| Other/Prefer not to say | 2 (4.4) | 0 (0.0) | 4 (8.3) | 6 (0.5) |  |
| **Marital status** |  |  |  |  | 0.04 |
| Single | 279 (40.9) | 139 (39.8) | 258 (63.1) | 676 (56.1) |  |
| Not single | 242 (59.1) | 102 (60.2) | 184 (36.9) | 528 (43.9) |  |
| *Missing* | *7* | *4* | *10* | *21* |  |
| **Number of children** |  |  |  |  | 0.32 |
| 0 | 218 (31.3) | 106 (25.7) | 201 (38.7) | 525 (43.4) |  |
| 1 | 58 (11.7) | 27 (7.9) | 55 (5.1) | 140 (11.6) |  |
| 2 | 104 (10.1) | 51 (26.6) | 76 (22.0) | 231 (19.1) |  |
| 3 and over | 144 (46.9) | 55 (39.9) | 116 (34.2) | 315 (26.0) |  |
| *Missing* | *4* | *6* | *4* | *14* |  |
| **Education level** |  |  |  |  | 0.89 |
| Less than primary completed | 0 (0.0) | 1 (1.1) | 0 (0.0) | 1 (0.1) |  |
| Primary completed | 3 (2.9) | 2 (4.0) | 5 (5.5) | 10 (0.8) |  |
| Secondary completed | 137 (29.9) | 71 (26.1) | 142 (29.1) | 350 (28.6) |  |
| University completed | 388 (67.2) | 171 (68.8) | 305 (65.4) | 864 (70.5) |  |
| *Missing* |  |  |  |  |  |
| **Employment status** |  |  |  |  | 0.58 |
| Employed | 336 (54.3) | 152 (65.6) | 260 (58.1) | 748 (61.1) |  |
| Not employed | 192 (45.7) | 93 (34.4) | 192 (41.9) | 477 (38.9) |  |
| **Quintiles for PPP-adjusted equivalised gross annual income** |  |  |  |  | 0.17 |
| Quintile 1 (low) | 74 (26.8) | 48 (22.9) | 107 (29.7) | 229 (20.7) |  |
| Quintile 2 | 105 (18.5) | 44 (21.8) | 100 (25.5) | 249 (22.5) |  |
| Quintile 3 | 79 (6.1) | 46 (22.9) | 59 (3.9) | 184 (16.6) |  |
| Quintile 4 | 101 (24.5) | 48 (24.9) | 80 (22.1) | 229 (20.7) |  |
| Quintile 5 (high) | 110 (24.1) | 37 (7.5) | 69 (18.8) | 216 (19.5) |  |
| *Missing* | *59* | *22* | *37* | *118* |  |

Notes: Frequencies are unweighted and percentages are weighted, except for total column, in which both are unweighted.

Percentages do not sum to 100 due to rounding

| **Table S23.** Personal COVID-19 experience, health risk attitude, and political ideology of participants in the CANDOUR II study (Ghana) by early life COVID-19 vaccine mandate attitude | | | | | |
| --- | --- | --- | --- | --- | --- |
| **Early life COVID-19 vaccine mandate attitude** | **Disagree**  **n=528** | **Neutral**  **n=245** | **Agree**  **n=452** | **Total**  **N=1,225** | **P-value** |
| **Number of participants** | Unweighted frequency **(weighted %)** | Unweighted frequency **(weighted %)** | Unweighted frequency **(weighted %)** | Unweighted frequency **(unweighted %)** |  |
| **COVID-19 vaccination status** |  |  |  |  | 0.05 |
| Vaccinated | 336 (76.8) | 185 (88.3) | 379 (94.9) | 900 (83.5) |  |
| Waiting for vaccination | 30 (3.1) | 9 (3.9) | 23 (2.7) | 62 (5.8) |  |
| Declined | 69 (12.0) | 18 (7.8) | 7 (0.1) | 94 (8.7) |  |
| Prefer not to say | 15 (8.2) | 3 (0.0004) | 4 (2.4) | 22 (2.0) |  |
| *Missing* | *78* | *30* | *39* | *147* |  |
| **Side Effects** |  |  |  |  | 0.56 |
| No side effects | 266 (79.4) | 137 (68.9) | 280 (69.1) | 683 (75.9) |  |
| Side effects | 70 (20.6) | 48 (31.1) | 99 (30.9) | 217 (24.1) |  |
| Not vaccinated | 114 | 30 | 34 | 178 |  |
| *Missing* | *78* | *30* | *39* | *147* |  |
| **Reasons for Vaccinating** |  |  |  |  |  |
| To protect myself | 238 (67.5) | 158 (86.6) | 307 (80.4) | 703 (78.1) | 0.20 |
| To protect my family | 189 (39.8) | 112 (66.7) | 216 (56.5) | 517 (57.4) | 0.11 |
| To protect the public | 135 (28.5) | 73 (48.7) | 172 (40.6) | 380 (42.2) | 0.28 |
| To travel and visit people/places | 113 (29.7) | 69 (40.1) | 140 (29.5) | 322 (35.8) | 0.62 |
| Because everyone else will | 9 (1.1) | 8 (1.1) | 13 (1.2) | 30 (3.3) | 0.93 |
| Recommended by friends/family | 17 (0.4) | 9 (4.5) | 26 (5.3) | 52 (5.8) | 0.27 |
| Recommended by healthcare officials/professionals | 70 (14.9) | 49 (14.4) | 89 (23.8) | 208 (23.1) | 0.50 |
| Recommended by politicians | 15 (3.6) | 2 (0.0001) | 13 (0.5) | 30 (3.3) | 0.004 |
| Contact with/symptoms of COVID-19 | 31 (10.8) | 25 (17.5) | 52 (10.7) | 108 (12.0) | 0.67 |
| Work/school requirement | 82 (25.1) | 42 (13.6) | 76 (16.2) | 200 (22.2) | 0.48 |
| Other reason(s) | 4 (0.2) | 0 (0.0) | 3 (0.5) | 7 (0.8) | 0.48 |
| **Personal health risk attitude** |  |  |  |  | 0.10 |
| Unwilling | 392 (76.7) | 157 (65.3) | 294 (53.8) | 843 (69.3) |  |
| Neutral | 79 (9.9) | 52 (17.5) | 66 (13.4) | 197 (16.2) |  |
| Willing | 53 (13.4) | 33 (17.2) | 91 (32.8) | 177 (14.5) |  |
| *Missing* | *4* | *3* | *1* | *8* |  |
| **Does participant know anyone who died of COVID-19?** |  |  |  |  | 0.91 |
| Yes | 131 (18.7) | 73 (22.2) | 118 (21.5) | 322 (26.8) |  |
| No | 386 (81.3) | 166 (77.8) | 326 (78.5) | 878 (73.2) |  |
| *Missing* | *11* | *6* | *8* | *25* |  |
| **Ideology** |  |  |  |  | 0.06 |
| Left | 125 (22.4) | 43 (19.5) | 76 (14.0) | 244 (19.9) |  |
| Centre | 274 (54.9) | 137 (54.7) | 216 (37.2) | 627 (51.2) |  |
| Right | 129 (22.7) | 65 (25.8) | 160 (48.8) | 354 (28.9) |  |
| *Missing* | *0* | *0* | *0* | *0* |  |

Note: Frequencies are unweighted and percentages are weighted, except for total column, in which both are unweighted. Percentages do not sum to 100 due to rounding

**Table S24.** Other COVID-19 vaccine mandate attitudes of participants in the CANDOUR II study (Ghana) by early life COVID-19 vaccine mandate attitude

| **Early life COVID-19 vaccine mandate attitude** | **Disagree**  **n=528** | **Neutral**  **n=245** | **Agree**  **n=452** | **Total**  **N=1,225** | **P-value** |
| --- | --- | --- | --- | --- | --- |
|  | Unweighted frequency **(weighted %)** | Unweighted frequency **(weighted %)** | Unweighted frequency **(weighted %)** | Unweighted frequency **(unweighted %)** |  |
| **Schoolchild COVID-19 vaccine mandate attitude** |  |  |  |  |  |
| Disagree | 327 (59.9) | 51 (16.4) | 34 (2.5) | 412 (34.0) |  |
| Neutral | 120 (19.4) | 102 (47.6) | 91 (19.8) | 313 (25.9) | <0.001 |
| Agree | 75 (20.8) | 89 (36.0) | 321 (77.8) | 485 (40.1) |  |
| *Missing* | *6* | *3* | *6* | *15* |  |
| **Governmental COVID-19 vaccine mandate attitude** |  |  |  |  |  |
| Disagree | 348 (70.2) | 51 (19.5) | 58 (13.2) | 457 (37.6) |  |
| Neutral | 99 (11.2) | 119 (56.9) | 84 (11.6) | 302 (24.9) | <0.001 |
| Agree | 78 (18.6) | 73 (23.6) | 304 (75.2) | 455 (37.5) |  |
| *Missing* | *3* | *2* | *6* | *11* |  |
| **COVID-19 vaccination should be a personal choice** |  |  |  |  |  |
| Disagree | 146 (29.8) | 75 (38.7) | 202 (48.7) | 423 (34.9) |  |
| Neutral | 108 (25.4) | 93 (29.3) | 103 (27.6) | 304 (25.1) | 0.21 |
| Agree | 271 (44.8) | 73 (32.0) | 142 (23.7) | 486 (40.1) |  |
| *Missing* | *3* | *4* | *5* | *12* |  |

Notes: Frequencies are unweighted and percentages are weighted, except for total column, in which both are unweighted.

Percentages do not sum to 100 due to rounding

**INDIA**

**Table S25.** Sociodemographic characteristics of participants in the CANDOUR II study (India) by early life COVID-19 vaccine mandate attitude

| **Early life COVID-19 vaccine mandate attitude** | **Disagree**  **n=115** | **Neutral**  **n=310** | **Agree**  **n=981** | **Total**  **N=1,406** | **P-value** |
| --- | --- | --- | --- | --- | --- |
|  | Unweighted frequency **(weighted %)** | Unweighted frequency **(weighted %)** | Unweighted frequency **(weighted %)** | Unweighted frequency **(unweighted %)** |  |
| **Age (years)** |  |  |  |  | 0.09 |
| 18-24 | 26 (43.7) | 62 (21.4) | 220 (25.8) | 308 (21.9) |  |
| 25-34 | 46 (26.7) | 145 (43.0) | 509 (36.7) | 700 (49.8) |  |
| 35-44 | 20 (11.0) | 78 (10.6) | 175 (25.0) | 273 (19.4) |  |
| 45-54 | 14 (9.3) | 18 (23.4) | 51 (6.2) | 83 (5.9) |  |
| 55-64 | 7 (7.4) | 6 (1.3) | 20 (4.8) | 33 (2.3) |  |
| 65 and over | 2 (2.0) | 1 (0.3) | 6 (1.7) | 9 (0.6) |  |
| **Gender** |  |  |  |  | 0.38 |
| Woman | 43 (39.4) | 145 (28.3) | 528 (37.2) | 716 (50.9) |  |
| Man | 72 (60.6) | 163 (36.2) | 451 (45.7) | 686 (48.8) |  |
| Other/Prefer not to say | 0 (0.0) | 2 (35.5) | 2 (17.1) | 4 (0.3) |  |
| **Marital status** |  |  |  |  | 0.83 |
| Single | 44 (53.2) | 123 (54.5) | 362 (49.2) | 529 (37.7) |  |
| Not single | 71 (46.8) | 187 (45.5) | 618 (50.8) | 876 (62.3) |  |
| *Missing* | *0* | *0* | *1* |  |  |
| **Number of children** |  |  |  |  | 0.05 |
| 0 | 43 (32.8) | 110 (23.1) | 383 (31.8) | 536 (38.9) |  |
| 1 | 44 (38.6) | 97 (15.0) | 344 (36.1) | 485 (35.2) |  |
| 2 | 22 (10.3) | 82 (37.0) | 196 (28.1) | 300 (21.8) |  |
| 3 and over | 5 (18.4) | 13 (24.9) | 39 (3.9) | 57 (4.1) |  |
| *Missing* | *1* | *8* | *19* | *28* |  |
| **Education level** |  |  |  |  | 0.07 |
| Less than primary completed | 1 (17.0) | 3 (12.2) | 0 (0.0) | 4 (0.3) |  |
| Primary completed | 2 (8.3) | 12 (35.5) | 11 (21.4) | 25 (1.8) |  |
| Secondary completed | 11 (31.3) | 15 (10.0) | 59 (22.4) | 85 (6.0) |  |
| University completed | 101 (43.5) | 280 (42.3) | 911 (56.2) | 1,292 (91.9) |  |
| *Missing* | *0* | *0* | *0* |  |  |
| **Employment status** |  |  |  |  | 0.17 |
| Employed | 92 (77.6) | 231 (84.0) | 765 (74.9) | 1,088 (77.4) |  |
| Not employed | 23 (22.4) | 79 (16.0) | 216 (25.1) | 318 (22.6) |  |
| **Quintiles for PPP-adjusted equivalised gross annual income** |  |  |  |  | 0.002 |
| Quintile 1 (low) | 22 (34.5) | 81 (38.5) | 199 (40.1) | 302 (21.8) |  |
| Quintile 2 | 13 (6.1) | 65 (44.1) | 176 (11.2) | 254 (18.4) |  |
| Quintile 3 | 33 (23.0) | 60 (7.3) | 209 (19.3) | 302 (21.8) |  |
| Quintile 4 | 27 (19.5) | 62 (6.4) | 221 (12.0) | 310 (22.4) |  |
| Quintile 5 (high) | 19 (14.0) | 39 (3.7) | 158 (17.4) | 216 (15.6) |  |
| *Missing* |  |  |  |  |  |

Notes: Frequencies are unweighted and percentages are weighted, except for total column, in which both are unweighted.

Percentages do not sum to 100 due to rounding

| **Table S26.** Personal COVID-19 experience, health risk attitude, and political ideology of participants in the CANDOUR II study (India) by early life COVID-19 vaccine mandate attitude | | | | | |
| --- | --- | --- | --- | --- | --- |
| **Early life COVID-19 vaccine mandate attitude** | **Disagree**  **n=115** | **Neutral**  **n=310** | **Agree**  **n=981** | **Total**  **N=1,406** | **P-value** |
| **Number of participants** | Unweighted frequency **(weighted %)** | Unweighted frequency **(weighted %)** | Unweighted frequency **(weighted %)** | Unweighted frequency **(unweighted %)** |  |
| **COVID-19 vaccination status** |  |  |  |  | 0.002 |
| Vaccinated | 92 (90.7) | 251 (98.5) | 889 (99.9) | 1,232 (98.6) |  |
| Waiting for vaccination | 6 (7.8) | 4 (0.5) | 1 (0.1) | 11 (0.9) |  |
| Declined | 2 (0.7) | 1 (0.1) | 0 (0.0) | 3 (0.2) |  |
| Prefer not to say | 2 (1.0) | 1 (0.9) | 0 (0.0) | 3 (0.2) |  |
| *Missing* | *13* | *53* | *91* | *157* |  |
| **Side Effects** |  |  |  |  | 0.76 |
| No side effects | 69 (73.2) | 191 (67.7) | 654 (74.1) | 914 (74.2) |  |
| Side effects | 23 (26.8) | 60 (32.3) | 235 (25.9) | 318 (25.8) |  |
| Not vaccinated | 10 | 6 | 1 | 17 |  |
| *Missing* | *13* | *53* | *91* | *157* |  |
| **Reasons for Vaccinating** |  |  |  |  |  |
| To protect myself | 74 (85.6) | 197 (59.0) | 752 (67.5) | 1,023 (83.0) | 0.48 |
| To protect my family | 66 (51.4) | 186 (37.1) | 715 (74.4) | 967 (78.5) | 0.02 |
| To protect the public | 40 (38.6) | 98 (22.3) | 427 (44.1) | 565 (45.9) | 0.11 |
| To travel and visit people/places | 46 (30.5) | 130 (43.1) | 464 (43.6) | 640 (51.9) | 0.76 |
| Because everyone else will | 14 (0.6) | 52 (9.3) | 173 (21.0) | 239 (19.4) | 0.51 |
| Recommended by friends/family | 23 (15.1) | 43 (6.2) | 180 (23.4) | 246 (20.0) | 0.01 |
| Recommended by healthcare officials/professionals | 35 (28.2) | 95 (43.5) | 360 (48.2) | 490 (39.8) | 0.61 |
| Recommended by politicians | 10 (4.4) | 27 (49.6) | 117 (18.1) | 154 (12.5) | 0.04 |
| Contact with/symptoms of COVID-19 | 17 (7.9) | 69 (21.6) | 239 (32.7) | 325 (26.4) | 0.20 |
| Work/school requirement | 29 (24.5) | 50 (11.0) | 194 (22.3) | 273 (22.2) | 0.33 |
| Other reason(s) | 0 (0.0) | 1 (0.0005) | 6 (0.1) | 7 (0.6) | 0.38 |
| **Personal health risk attitude** |  |  |  |  | 0.003 |
| Unwilling | 57 (52.4) | 61 (30.6) | 251 (25.6) | 369 (26.6) |  |
| Neutral | 36 (35.5) | 138 (53.5) | 163 (12.2) | 337 (24.3) |  |
| Willing | 20 (12.1) | 106 (15.9) | 556 (62.2) | 682 (49.1) |  |
| *Missing* | *2* | *5* | *11* | *18* |  |
| **Does participant know anyone who died of COVID-19?** |  |  |  |  | 0.67 |
| Yes | 70 (77.7) | 186 (63.2) | 671 (68.9) | 927 (68.2) |  |
| No | 39 (22.3) | 108 (36.8) | 286 (31.1) | 433 (31.8) |  |
| *Missing* | *6* | *16* | *24* | *46* |  |
| **Ideology** |  |  |  |  | 0.002 |
| Left | 28 (37.0) | 24 (21.3) | 42 (2.5) | 94 (6.7) |  |
| Centre | 51 (40.0) | 137 (45.7) | 227 (26.4) | 415 (29.5) |  |
| Right | 36 (23.1) | 149 (33.1) | 712 (71.1) | 897 (63.8) |  |
| *Missing* | *0* | *0* | *0* | *0* |  |

Note: Frequencies are unweighted and percentages are weighted, except for total column, in which both are unweighted. Percentages do not sum to 100 due to rounding

**Table S27.** Other COVID-19 vaccine mandate attitudes of participants in the CANDOUR II study (India) by early life COVID-19 vaccine mandate attitude

| **Early life COVID-19 vaccine mandate attitude** | **Disagree**  **n=115** | **Neutral**  **n=310** | **Agree**  **n=981** | **Total**  **N=1,406** | **P-value** |
| --- | --- | --- | --- | --- | --- |
|  | Unweighted frequency **(weighted %)** | Unweighted frequency **(weighted %)** | Unweighted frequency **(weighted %)** | Unweighted frequency **(unweighted %)** |  |
| **Schoolchild COVID-19 vaccine mandate attitude** |  |  |  |  |  |
| Disagree | 49 (46.0) | 30 (25.4) | 14 (0.7) | 93 (6.6) |  |
| Neutral | 27 (27.0) | 169 (57.3) | 111 (9.8) | 307 (21.9) | <0.001 |
| Agree | 38 (27.0) | 108 (17.3) | 854 (89.5) | 1,000 (71.4) |  |
| *Missing* | *1* | *3* | *2* | *6* |  |
| **Governmental COVID-19 vaccine mandate attitude** |  |  |  |  |  |
| Disagree | 49 (35.6) | 24 (10.5) | 18 (3.0) | 91 (6.5) |  |
| Neutral | 20 (35.8) | 166 (70.4) | 99 (15.0) | 285 (20.3) | <0.001 |
| Agree | 45 (28.6) | 119 (19.1) | 862 (82.0) | 1,026 (73.2) |  |
| *Missing* | *1* | *1* | *2* | *4* |  |
| **COVID-19 vaccination should be a personal choice** |  |  |  |  |  |
| Disagree | 41 (38.0) | 39 (25.4) | 191 (23.7) | 271 (19.5) |  |
| Neutral | 27 (22.2) | 177 (63.4) | 151 (9.0) | 355 (25.5) | <0.001 |
| Agree | 46 (39.8) | 91 (11.2) | 630 (67.4) | 767 (55.1) |  |
| *Missing* | *1* | *3* | *9* | *13* |  |

Notes: Frequencies are unweighted and percentages are weighted, except for total column, in which both are unweighted.

Percentages do not sum to 100 due to rounding

**ITALY**

**Table S28.** Sociodemographic characteristics of participants in the CANDOUR II study (Italy) by early life COVID-19 vaccine mandate attitude

| **Early life COVID-19 vaccine mandate attitude** | **Disagree**  **n=481** | **Neutral**  **n=307** | **Agree**  **n=459** | **Total**  **N=1,247** | **P-value** |
| --- | --- | --- | --- | --- | --- |
|  | Unweighted frequency **(weighted %)** | Unweighted frequency **(weighted %)** | Unweighted frequency **(weighted %)** | Unweighted frequency **(unweighted %)** |  |
| **Age (years)** |  |  |  |  | 0.01 |
| 18-24 | 37 (7.4) | 36 (5.5) | 20 (0.3) | 93 (7.5) |  |
| 25-34 | 84 (14.5) | 65 (25.2) | 58 (5.9) | 207 (16.6) |  |
| 35-44 | 85 (10.6) | 58 (13.2) | 81 (18.0) | 224 (18.0) |  |
| 45-54 | 92 (24.1) | 58 (25.2) | 114 (22.0) | 264 (21.2) |  |
| 55-64 | 111 (35.2) | 47 (15.5) | 112 (30.5) | 270 (21.7) |  |
| 65 and over | 72 (8.3) | 43 (15.4) | 74 (23.4) | 189 (15.2) |  |
| **Gender** |  |  |  |  | 0.01 |
| Woman | 293 (68.6) | 153 (51.1) | 242 (44.3) | 688 (55.2) |  |
| Man | 188 (31.4) | 154 (48.9) | 217 (55.7) | 559 (44.8) |  |
| Other/Prefer not to say |  |  |  |  |  |
| **Marital status** |  |  |  |  | 0.61 |
| Single | 183 (38.4) | 114 (38.5) | 149 (31.0) | 446 (36.5) |  |
| Not single | 289 (61.6) | 182 (61.5) | 306 (69.0) | 777 (63.5) |  |
| *Missing* | *9* | *11* | *4* | *24* |  |
| **Number of children** |  |  |  |  | 0.22 |
| 0 | 287 (53.9) | 193 (68.2) | 271 (68.6) | 751 (61.0) |  |
| 1 | 102 (25.9) | 55 (15.9) | 89 (19.9) | 246 (20.0) |  |
| 2 | 72 (14.6) | 41 (15.7) | 73 (9.5) | 186 (15.1) |  |
| 3 and over | 16 (5.7) | 11 (0.3) | 22 (2.0) | 49 (4.0) |  |
| *Missing* | *4* | *7* | *4* | *15* |  |
| **Education level** |  |  |  |  | 0.79 |
| Less than primary completed | 1 (0.0002) | 1 (0.0003) | 0 (0.0) | 2 (0.2) |  |
| Primary completed | 3 (0.1) | 4 (0.2) | 4 (0.1) | 11 (0.9) |  |
| Secondary completed | 318 (87.7) | 186 (83.5) | 285 (84.2) | 789 (63.5) |  |
| University completed | 158 (12.2) | 115 (16.3) | 167 (15.6) | 440 (35.4) |  |
| *Missing* | *1* | *1* | *3* | *5* |  |
| **Employment status** |  |  |  |  | 0.13 |
| Employed | 265 (49.3) | 181 (65.8) | 262 (47.5) | 708 (56.8) |  |
| Not employed | 216 (50.7) | 126 (34.2) | 197 (52.5) | 539 (43.2) |  |
| **Quintiles for PPP-adjusted equivalised gross annual income** |  |  |  |  | 0.35 |
| Quintile 1 (low) | 93 (26.4) | 72 (19.6) | 74 (16.1) | 239 (21.7) |  |
| Quintile 2 | 90 (21.1) | 58 (11.8) | 93 (18.2) | 241 (21.8) |  |
| Quintile 3 | 82 (21.3) | 59 (21.5) | 79 (28.6) | 220 (19.9) |  |
| Quintile 4 | 81 (14.3) | 59 (33.9) | 90 (19.4) | 230 (20.9) |  |
| Quintile 5 (high) | 58 (17.0) | 35 (13.3) | 80 (17.7) | 173 (15.7) |  |
| *Missing* | *77* | *24* | *43* | *144* |  |

Notes: Frequencies are unweighted and percentages are weighted, except for total column, in which both are unweighted.

Percentages do not sum to 100 due to rounding

| **Table S29.** Personal COVID-19 experience, health risk attitude, and political ideology of participants in the CANDOUR II study (Italy) by early life COVID-19 vaccine mandate attitude | | | | | |
| --- | --- | --- | --- | --- | --- |
| **Early life COVID-19 vaccine mandate attitude** | **Disagree**  **n=481** | **Neutral**  **n=307** | **Agree**  **n=459** | **Total**  **N=1,247** | **P-value** |
| **Number of participants** | Unweighted frequency **(weighted %)** | Unweighted frequency **(weighted %)** | Unweighted frequency **(weighted %)** | Unweighted frequency **(unweighted %)** |  |
| **COVID-19 vaccination status** |  |  |  |  | 0.02 |
| Vaccinated | 361 (88.7) | 239 (99.96) | 415 (98.2) | 1,015 (95.6) |  |
| Waiting for vaccination | 0 (0.0) | 1 (0.0003) | 1 (0.0003) | 2 (0.2) |  |
| Declined | 36 (11.2) | 1 (0.0001) | 3 (1.8) | 40 (3.8) |  |
| Prefer not to say | 5 (0.0006) | 0 (0.0) | 0 (0.0) | 5 (0.5) |  |
| *Missing* | *79* | *66* | *40* | *185* |  |
| **Side Effects** |  |  |  |  | 0.001 |
| No side effects | 288 (86.4) | 186 (70.3) | 293 (54.2) | 767 (75.6) |  |
| Side effects | 73 (13.7) | 53 (29.7) | 122 (45.8) | 248 (24.4) |  |
| Not vaccinated | 41 | 2 | 4 | 47 |  |
| *Missing* | *79* | *66* | *40* | *185* |  |
| **Reasons for Vaccinating** |  |  |  |  |  |
| To protect myself | 218 (52.4) | 185 (73.6) | 364 (83.3) | 767 (75.6) | 0.001 |
| To protect my family | 207 (52.1) | 167 (72.8) | 322 (72.2) | 696 (68.6) | 0.03 |
| To protect the public | 83 (24.0) | 68 (32.8) | 158 (36.1) | 309 (30.4) | 0.33 |
| To travel and visit people/places | 100 (26.0) | 64 (20.3) | 134 (28.7) | 298 (29.4) | 0.67 |
| Because everyone else will | 5 (0.7) | 11 (0.7) | 15 (0.8) | 31 (3.1) | 0.88 |
| Recommended by friends/family | 7 (0.1) | 7 (0.3) | 22 (2.2) | 36 (3.5) | <0.001 |
| Recommended by healthcare officials/professionals | 91 (24.2) | 72 (29.9) | 172 (32.8) | 335 (33.0) | 0.56 |
| Recommended by politicians | 12 (3.7) | 11 (0.4) | 21 (3.9) | 44 (4.3) | 0.32 |
| Contact with/symptoms of COVID-19 | 8 (3.7) | 14 (6.6) | 14 (3.7) | 36 (3.5) | 0.76 |
| Work/school requirement | 106 (24.6) | 44 (32.0) | 59 (16.6) | 209 (20.6) | 0.22 |
| Other reason(s) | 16 (7.3) | 5 (0.4) | 3 (0.0004) | 24 (2.4) | <0.001 |
| **Personal health risk attitude** |  |  |  |  | 0.002 |
| Unwilling | 304 (70.2) | 127 (34.8) | 244 (64.6) | 675 (56.0) |  |
| Neutral | 109 (17.9) | 123 (44.5) | 100 (18.4) | 332 (27.5) |  |
| Willing | 50 (11.9) | 45 (20.7) | 104 (17.0) | 199 (16.5) |  |
| *Missing* | *18* | *12* | *11* | *41* |  |
| **Does participant know anyone who died of COVID-19?** |  |  |  |  | 0.18 |
| Yes | 187 (34.4) | 148 (46.2) | 232 (49.1) | 567 (46.8) |  |
| No | 280 (65.6) | 145 (53.8) | 219 (50.9) | 644 (53.2) |  |
| *Missing* | *14* | *14* | *8* | *36* |  |
| **Ideology** |  |  |  |  | 0.53 |
| Left | 115 (24.6) | 71 (17.0) | 118 (30.3) | 304 (28.1) |  |
| Centre | 131 (43.2) | 140 (50.3) | 153 (35.2) | 424 (39.2) |  |
| Right | 142 (32.2) | 69 (32.7) | 142 (34.5) | 353 (32.7) |  |
| *Missing* | *93* | *27* | *46* | *166* |  |

Note: Frequencies are unweighted and percentages are weighted, except for total column, in which both are unweighted. Percentages do not sum to 100 due to rounding

**Table S30.** Other COVID-19 vaccine mandate attitudes of participants in the CANDOUR II study (Italy) by early life COVID-19 vaccine mandate attitude

| **Early life COVID-19 vaccine mandate attitude** | **Disagree**  **n=481** | **Neutral**  **n=307** | **Agree**  **n=459** | **Total**  **N=1,247** | **P-value** |
| --- | --- | --- | --- | --- | --- |
|  | Unweighted frequency **(weighted %)** | Unweighted frequency **(weighted %)** | Unweighted frequency **(weighted %)** | Unweighted frequency **(unweighted %)** |  |
| **Schoolchild COVID-19 vaccine mandate attitude** |  |  |  |  |  |
| Disagree | 341 (76.5) | 30 (13.4) | 23 (3.8) | 394 (32.8) |  |
| Neutral | 63 (10.6) | 161 (54.1) | 65 (9.6) | 289 (24.0) |  |
| Agree | 57 (12.9) | 104 (32.6) | 358 (86.6) | 519 (43.2) | <0.001 |
| *Missing* | *20* | *12* | *13* | *45* |  |
| **Governmental COVID-19 vaccine mandate attitude** |  |  |  |  |  |
| Disagree | 303 (68.8) | 20 (12.4) | 21 (2.1) | 344 (27.8) |  |
| Neutral | 57 (6.3) | 151 (37.0) | 46 (12.5) | 254 (20.5) | <0.001 |
| Agree | 117 (25.0) | 133 (50.6) | 391 (85.5) | 641 (51.7) |  |
| *Missing* | *4* | *3* | *1* | *8* |  |
| **COVID-19 vaccination should be a personal choice** |  |  |  |  |  |
| Disagree | 119 (27.4) | 82 (37.5) | 214 (45.3) | 415 (33.7) |  |
| Neutral | 81 (14.7) | 153 (44.4) | 97 (26.9) | 331 (26.9) | <0.001 |
| Agree | 276 (58.0) | 68 (18.2) | 142 (27.8) | 486 (39.4) |  |
| *Missing* |  |  |  |  |  |

Notes: Frequencies are unweighted and percentages are weighted, except for total column, in which both are unweighted.

Percentages do not sum to 100 due to rounding

**JAPAN**

**Table S31.** Sociodemographic characteristics of participants in the CANDOUR II study (Japan) by early life COVID-19 vaccine mandate attitude

| **Early life COVID-19 vaccine mandate attitude** | **Disagree**  **n=725** | **Neutral**  **n=252** | **Agree**  **n=127** | **Total**  **N=1,104** | **P-value** |
| --- | --- | --- | --- | --- | --- |
|  | Unweighted frequency **(weighted %)** | Unweighted frequency **(weighted %)** | Unweighted frequency **(weighted %)** | Unweighted frequency **(unweighted %)** |  |
| **Age (years)** |  |  |  |  | 0.37 |
| 18-24 | 49 (7.5) | 25 (2.3) | 5 (0.9) | 79 (7.2) |  |
| 25-34 | 100 (17.3) | 28 (18.0) | 21 (10.1) | 149 (13.5) |  |
| 35-44 | 137 (7.1) | 41 (6.8) | 17 (10.0) | 195 (17.7) |  |
| 45-54 | 126 (19.1) | 41 (19.0) | 15 (4.8) | 182 (16.5) |  |
| 55-64 | 223 (14.7) | 70 (28.1) | 46 (56.1) | 339 (30.7) |  |
| 65 and over | 90 (34.3) | 47 (25.9) | 23 (18.3) | 160 (14.5) |  |
| **Gender** |  |  |  |  | 0.57 |
| Woman | 373 (47.4) | 106 (43.1) | 52 (59.1) | 531 (48.1) |  |
| Man | 350 (39.8) | 144 (28.7) | 75 (40.9) | 569 (51.5) |  |
| Other/Prefer not to say | 2 (12.8) | 2 (28.2) | 0 (0.0) | 4 (0.4) |  |
| **Marital status** |  |  |  |  | 0.16 |
| Single | 281 (41.2) | 103 (23.5) | 44 (61.2) | 428 (39.7) |  |
| Not single | 431 (58.8) | 138 (76.5) | 80 (38.8) | 649 (60.3) |  |
| *Missing* | *13* | *11* | *3* | *27* |  |
| **Number of children** |  |  |  |  | 0.01 |
| 0 | 503 (80.0) | 184 (55.3) | 96 (44.3) | 783 (72.3) |  |
| 1 | 121 (8.1) | 34 (40.6) | 20 (50.1) | 175 (16.2) |  |
| 2 | 71 (10.7) | 21 (3.7) | 6 (4.6) | 98 (9.0) |  |
| 3 and over | 18 (1.2) | 5 (0.5) | 4 (1.1) | 27 (2.5) |  |
| *Missing* | *12* | *8* | *1* | *21* |  |
| **Education level** |  |  |  |  | 0.54 |
| Less than primary completed | 0 (0.0) | 0 (0.0) | 0 (0.0) | 0 (0.0) |  |
| Primary completed | 32 (3.5) | 16 (2.1) | 3 (7.7) | 51 (4.7) |  |
| Secondary completed | 392 (68.4) | 136 (60.0) | 80 (67.5) | 608 (55.6) |  |
| University completed | 294 (28.1) | 98 (38.3) | 43 (24.9) | 435 (39.8) |  |
| *Missing* | *7* | *2* | *1* | *10* |  |
| **Employment status** |  |  |  |  | 0.13 |
| Employed | 531 (80.0) | 184 (59.5) | 92 (45.7) | 807 (73.1) |  |
| Not employed | 194 (20.0) | 68 (40.5) | 35 (54.3) | 297 (26.9) |  |
| **Quintiles for PPP-adjusted equivalised gross annual income** |  |  |  |  | 0.50 |
| Quintile 1 (low) | 153 (30.1) | 52 (36.7) | 25 (52.3) | 230 (24.1) |  |
| Quintile 2 | 88 (9.6) | 38 (9.5) | 17 (15.7) | 143 (15.0) |  |
| Quintile 3 | 144 (26.8) | 58 (35.8) | 23 (12.0) | 225 (23.6) |  |
| Quintile 4 | 125 (24.7) | 40 (8.4) | 25 (14.4) | 190 (19.9) |  |
| Quintile 5 (high) | 111 (8.9) | 35 (9.6) | 21 (5.6) | 167 (17.5) |  |
| *Missing* | *104* | *29* | *16* | *149* |  |

Notes: Frequencies are unweighted and percentages are weighted, except for total column, in which both are unweighted.

Percentages do not sum to 100 due to rounding

| **Table S32.** Personal COVID-19 experience, health risk attitude, and political ideology of participants in the CANDOUR II study (Japan) by early life COVID-19 vaccine mandate attitude | | | | | |
| --- | --- | --- | --- | --- | --- |
| **Early life COVID-19 vaccine mandate attitude** | **Disagree**  **n=725** | **Neutral**  **n=252** | **Agree**  **n=127** | **Total**  **N=1,104** | **P-value** |
| **Number of participants** | Unweighted frequency **(weighted %)** | Unweighted frequency **(weighted %)** | Unweighted frequency **(weighted %)** | Unweighted frequency **(unweighted %)** |  |
| **COVID-19 vaccination status** |  |  |  |  | 0.09 |
| Vaccinated | 587 (94.1) | 198 (99.7) | 111 (99.7) | 896 (90.7) |  |
| Waiting for vaccination | 2 (0.2) | 0 (0.0) | 0 (0.0) | 2 (0.2) |  |
| Declined | 80 (5.5) | 4 (0.3) | 1 (0.3) | 85 (8.6) |  |
| Prefer not to say | 5 (0.2) | 0 (0.0) | 0 (0.0) | 5 (0.5) |  |
| *Missing* | *51* | *50* | *15* | *116* |  |
| **Side Effects** |  |  |  |  | 0.26 |
| No side effects | 542 (95.0) | 163 (93.5) | 96 (86.9) | 801 (89.4) |  |
| Side effects | 45 (5.0) | 35 (6.5) | 15 (13.1) | 95 (10.6) |  |
| Not vaccinated | 87 | 4 | 1 | 92 |  |
| *Missing* | *51* | *50* | *15* | *116* |  |
| **Reasons for Vaccinating** |  |  |  |  |  |
| To protect myself | 487 (84.4) | 170 (95.7) | 103 (95.6) | 760 (84.8) | 0.04 |
| To protect my family | 411 (70.4) | 143 (75.0) | 80 (72.3) | 634 (70.8) | 0.84 |
| To protect the public | 177 (22.1) | 66 (30.8) | 33 (18.7) | 276 (30.8) | 0.58 |
| To travel and visit people/places | 240 (40.9) | 68 (31.7) | 40 (40.8) | 348 (38.8) | 0.68 |
| Because everyone else will | 96 (13.3) | 32 (1.8) | 16 (1.0) | 144 (16.1) | 0.04 |
| Recommended by friends/family | 28 (9.9) | 13 (2.1) | 8 (3.8) | 49 (5.5) | 0.09 |
| Recommended by healthcare officials/professionals | 25 (2.7) | 13 (1.5) | 7 (11.2) | 45 (5.0) | 0.02 |
| Recommended by politicians | 11 (1.2) | 1 (0.2) | 4 (2.4) | 16 (1.8) | 0.04 |
| Contact with/symptoms of COVID-19 | 7 (0.3) | 1 (0.1) | 2 (1.1) | 10 (1.1) | 0.13 |
| Work/school requirement | 103 (16.0) | 24 (5.7) | 11 (10.8) | 138 (15.4) | 0.17 |
| Other reason(s) | 16 (2.2) | 1 (0.001) | 3 (1.7) | 20 (2.2) | 0.002 |
| **Personal health risk attitude** |  |  |  |  | 0.60 |
| Unwilling | 334 (44.3) | 68 (43.9) | 28 (26.5) | 430 (43.7) |  |
| Neutral | 242 (44.2) | 126 (46.3) | 46 (41.3) | 414 (42.1) |  |
| Willing | 75 (11.6) | 28 (9.8) | 36 (32.3) | 139 (14.1) |  |
| *Missing* | *74* | *30* | *17* | *121* |  |
| **Does participant know anyone who died of COVID-19?** |  |  |  |  | 0.84 |
| Yes | 118 (6.9) | 31 (6.5) | 20 (9.6) | 169 (15.8) |  |
| No | 585 (93.1) | 212 (93.5) | 102 (90.4) | 899 (84.2) |  |
| *Missing* | *22* | *9* | *5* | *36* |  |
| **Ideology** |  |  |  |  | <0.001 |
| Left | 91 (29.5) | 19 (2.3) | 10 (11.2) | 120 (14.6) |  |
| Centre | 319 (49.5) | 141 (90.2) | 60 (48.6) | 520 (63.3) |  |
| Right | 114 (21.0) | 35 (7.5) | 33 (40.3) | 182 (22.1) |  |
| *Missing* | *201* | *57* | *24* | *282* |  |

Note: Frequencies are unweighted and percentages are weighted, except for total column, in which both are unweighted. Percentages do not sum to 100 due to rounding

**Table S33.** Other COVID-19 vaccine mandate attitudes of participants in the CANDOUR II study (Japan) by early life COVID-19 vaccine mandate attitude

| **Early life COVID-19 vaccine mandate attitude** | **Disagree**  **n=725** | **Neutral**  **n=252** | **Agree**  **n=127** | **Total**  **N=1,104** | **P-value** |
| --- | --- | --- | --- | --- | --- |
|  | Unweighted frequency **(weighted %)** | Unweighted frequency **(weighted %)** | Unweighted frequency **(weighted %)** | Unweighted frequency **(unweighted %)** |  |
| **Schoolchild COVID-19 vaccine mandate attitude** |  |  |  |  |  |
| Disagree | 519 (74.4) | 25 (19.9) | 6 (5.4) | 550 (52.8) |  |
| Neutral | 114 (21.5) | 163 (57.0) | 24 (25.5) | 301 (28.9) | <0.001 |
| Agree | 44 (4.1) | 55 (23.2) | 91 (69.2) | 190 (18.3) |  |
| *Missing* | *48* | *9* | *6* | *63* |  |
| **Governmental COVID-19 vaccine mandate attitude** |  |  |  |  |  |
| Disagree | 565 (90.5) | 26 (3.4) | 4 (5.5) | 595 (54.8) |  |
| Neutral | 98 (6.1) | 179 (73.8) | 21 (25.9) | 298 (27.4) | <0.001 |
| Agree | 49 (3.4) | 45 (22.8) | 99 (68.6) | 193 (17.8) |  |
| *Missing* | *13* | *2* | *3* | *18* |  |
| **COVID-19 vaccination should be a personal choice** |  |  |  |  |  |
| Disagree | 92 (13.6) | 36 (5.1) | 48 (57.3) | 176 (16.1) |  |
| Neutral | 141 (15.8) | 149 (67.4) | 30 (19.5) | 320 (29.3) | <0.001 |
| Agree | 488 (70.6) | 62 (27.5) | 45 (23.2) | 595 (54.5) |  |
| *Missing* | *4* | *5* | *4* | *13* |  |

Notes: Frequencies are unweighted and percentages are weighted, except for total column, in which both are unweighted.

Percentages do not sum to 100 due to rounding

**SOUTH AFRICA**

**Table S34.** Sociodemographic characteristics of participants in the CANDOUR II study (South Africa) by early life COVID-19 vaccine mandate attitude

| **Early life COVID-19 vaccine mandate attitude** | **Disagree**  **n=683** | **Neutral**  **n=237** | **Agree**  **n=369** | **Total**  **N=1,289** | **P-value** |
| --- | --- | --- | --- | --- | --- |
|  | Unweighted frequency **(weighted %)** | Unweighted frequency **(weighted %)** | Unweighted frequency **(weighted %)** | Unweighted frequency **(unweighted %)** |  |
| **Age (years)** |  |  |  |  | 0.002 |
| 18-24 | 108 (13.5) | 38 (22.9) | 55 (14.8) | 201 (15.6) |  |
| 25-34 | 174 (29.7) | 59 (14.9) | 96 (35.1) | 329 (25.5) |  |
| 35-44 | 187 (40.7) | 67 (15.3) | 91 (9.8) | 345 (26.8) |  |
| 45-54 | 141 (6.6) | 51 (35.9) | 72 (5.9) | 264 (20.5) |  |
| 55-64 | 54 (6.6) | 20 (8.7) | 44 (29.8) | 118 (9.2) |  |
| 65 and over | 19 (3.0) | 2 (2.3) | 11 (4.7) | 32 (2.5) |  |
| **Gender** |  |  |  |  | 0.53 |
| Woman | 353 (38.6) | 119 (63.2) | 186 (32.4) | 658 (51.0) |  |
| Man | 329 (50.7) | 118 (36.8) | 182 (44.3) | 629 (48.8) |  |
| Other/Prefer not to say | 1 (10.7) | 0 (0.0) | 1 (23.3) | 2 (0.2) |  |
| **Marital status** |  |  |  |  | 0.53 |
| Single | 299 (37.0) | 96 (39.7) | 144 (52.2) | 539 (42.0) |  |
| Not single | 382 (63.0) | 140 (60.3) | 223 (47.8) | 745 (58.0) |  |
| *Missing* | *2* | *1* | *2* | *5* |  |
| **Number of children** |  |  |  |  | 0.21 |
| 0 | 246 (46.4) | 60 (28.0) | 117 (57.9) | 423 (33.1) |  |
| 1 | 180 (13.3) | 70 (23.4) | 87 (26.4) | 337 (26.4) |  |
| 2 | 160 (16.4) | 72 (15.7) | 105 (10.1) | 337 (26.4) |  |
| 3 and over | 90 (23.9) | 34 (32.9) | 57 (5.6) | 181 (14.2) |  |
| *Missing* | *7* | *1* | *3* | *11* |  |
| **Education level** |  |  |  |  | 0.08 |
| Less than primary completed | 0 (0.0) | 0 (0.0) | 0 (0.0) | 0 (0.0) |  |
| Primary completed | 35 (49.4) | 18 (31.7) | 23 (23.8) | 76 (5.9) |  |
| Secondary completed | 403 (45.5) | 132 (62.1) | 206 (47.8) | 741 (57.5) |  |
| University completed | 244 (5.1) | 87 (6.1) | 140 (28.4) | 471 (36.6) |  |
| *Missing* | *1* | *0* | *0* | *1* |  |
| **Employment status** |  |  |  |  | 0.03 |
| Employed | 535 (84.5) | 183 (50.9) | 277 (71.0) | 995 (77.2) |  |
| Not employed | 148 (15.5) | 54 (49.1) | 92 (29.0) | 294 (22.8) |  |
| **Quintiles for PPP-adjusted equivalised gross annual income** |  |  |  |  | 0.02 |
| Quintile 1 (low) | 117 (38.3) | 48 (25.3) | 79 (35.8) | 244 (19.9) |  |
| Quintile 2 | 137 (36,6) | 44 (28.4) | 62 (12.1) | 243 (19.8) |  |
| Quintile 3 | 135 (12.2) | 59 (20.7) | 77 (11.5) | 271 (22.1) |  |
| Quintile 4 | 114 (7.2) | 36 (13.9) | 73 (9.3) | 223 (18.2) |  |
| Quintile 5 (high) | 142 (5.8) | 38 (11.7) | 65 (31.3) | 245 (20.0) |  |
| *Missing* | *38* | *12* | *13* | *63* |  |

Notes: Frequencies are unweighted and percentages are weighted, except for total column, in which both are unweighted.

Percentages do not sum to 100 due to rounding

| **Table S35.** Personal COVID-19 experience, health risk attitude, and political ideology of participants in the CANDOUR II study (South Africa) by early life COVID-19 vaccine mandate attitude | | | | | |
| --- | --- | --- | --- | --- | --- |
| **Early life COVID-19 vaccine mandate attitude** | **Disagree**  **n=683** | **Neutral**  **n=237** | **Agree**  **n=369** | **Total**  **N=1,289** | **P-value** |
| **Number of participants** | Unweighted frequency **(weighted %)** | Unweighted frequency **(weighted %)** | Unweighted frequency **(weighted %)** | Unweighted frequency **(unweighted %)** |  |
| **COVID-19 vaccination status** |  |  |  |  | <0.001 |
| Vaccinated | 401 (51.9) | 181 (87.0) | 319 (96.6) | 901 (80.2) |  |
| Waiting for vaccination | 19 (2.0) | 9 (3.8) | 6 (1.1) | 34 (3.0) |  |
| Declined | 155 (45.6) | 14 (6.1) | 11 (2.3) | 180 (16.0) |  |
| Prefer not to say | 5 (0.5) | 3 (3.1) | 0 (0.0) | 8 (0.7) |  |
| *Missing* | *103* | *30* | *33* | *166* |  |
| **Side Effects** |  |  |  |  | 0.002 |
| No side effects | 349 (91.0) | 146 (51.5) | 246 (88.6) | 741 (82.2) |  |
| Side effects | 52 (9.0) | 35 (48.5) | 73 (11.4) | 160 (17.8) |  |
| Not vaccinated | 179 | 26 | 17 | 222 |  |
| *Missing* | *103* | *30* | *33* | *166* |  |
| **Reasons for Vaccinating** |  |  |  |  |  |
| To protect myself | 244 (35.2) | 140 (82.9) | 280 (82.0) | 664 (73.7) | 0.002 |
| To protect my family | 234 (33.2) | 142 (80.6) | 265 (90.5) | 641 (71.1) | <0.001 |
| To protect the public | 129 (18.5) | 86 (35.2) | 184 (42.2) | 399 (44.3) | 0.17 |
| To travel and visit people/places | 152 (37.7) | 76 (26.5) | 165 (23.9) | 393 (43.6) | 0.51 |
| Because everyone else will | 8 (0.1) | 6 (0.4) | 25 (1.8) | 39 (4.3) | 0.01 |
| Recommended by friends/family | 20 (2.8) | 15 (5.7) | 30 (5.4) | 65 (7.2) | 0.46 |
| Recommended by healthcare officials/professionals | 89 (12.5) | 49 (13.8) | 112 (15.7) | 250 (27.7) | 0.85 |
| Recommended by politicians | 21 (2.6) | 15 (5.4) | 36 (6.5) | 72 (8.0) | 0.27 |
| Contact with/symptoms of COVID-19 | 45 (7.4) | 35 (13.8) | 84 (13.0) | 164 (18.2) | 0.42 |
| Work/school requirement | 137 (20.8) | 30 (10.2) | 67 (7.3) | 234 (26.0) | 0.04 |
| Other reason(s) | 7 (25.4) | 1 (0.7) | 2 (0.8) | 10 (1.1) | <0.001 |
| **Personal health risk attitude** |  |  |  |  | 0.03 |
| Unwilling | 479 (77.2) | 108 (31.6) | 202 (50.9) | 789 (61.6) |  |
| Neutral | 137 (19.0) | 99 (58.1) | 72 (35.1) | 308 (24.1) |  |
| Willing | 61 (3.8) | 29 (10.3) | 93 (14.1) | 183 (14.3) |  |
| *Missing* | *6* | *1* | *2* | *9* |  |
| **Does participant know anyone who died of COVID-19?** |  |  |  |  | 0.35 |
| Yes | 417 (61.0) | 161 (44.3) | 270 (42.9) | 848 (67.1) |  |
| No | 251 (39.0) | 72 (55.7) | 93 (57.1) | 416 (32.9) |  |
| *Missing* | *15* | *4* | *6* | *25* |  |
| **Ideology** |  |  |  |  | 0.01 |
| Left | 154 (46.7) | 40 (9.5) | 52 (30.6) | 246 (19.1) |  |
| Centre | 382 (44.1) | 153 (79.0) | 166 (29.1) | 701 (54.4) |  |
| Right | 147 (9.2) | 44 (11.6) | 151 (40.3) | 342 (26.5) |  |
| *Missing* | *0* | *0* | *0* | *0* |  |

Note: Frequencies are unweighted and percentages are weighted, except for total column, in which both are unweighted. Percentages do not sum to 100 due to rounding

**Table S36.** Other COVID-19 vaccine mandate attitudes of participants in the CANDOUR II study (South Africa) by early life COVID-19 vaccine mandate attitude

| **Early life COVID-19 vaccine mandate attitude** | **Disagree**  **n=683** | **Neutral**  **n=237** | **Agree**  **n=369** | **Total**  **N=1,289** | **P-value** |
| --- | --- | --- | --- | --- | --- |
|  | Unweighted frequency **(weighted %)** | Unweighted frequency **(weighted %)** | Unweighted frequency **(weighted %)** | Unweighted frequency **(unweighted %)** |  |
| **Schoolchild COVID-19 vaccine mandate attitude** |  |  |  |  |  |
| Disagree | 512 (77.6) | 55 (43.4) | 42 (6.3) | 609 (47.7) |  |
| Neutral | 102 (17.9) | 122 (37.6) | 64 (8.9) | 288 (22.5) | <0.001 |
| Agree | 61 (4.6) | 59 (18.9) | 261 (84.8) | 381 (29.8) |  |
| *Missing* | *8* | *1* | *2* | *11* |  |
| **Governmental COVID-19 vaccine mandate attitude** |  |  |  |  |  |
| Disagree | 572 (86.5) | 54 (17.3) | 58 (6.1) | 684 (53.5) |  |
| Neutral | 56 (3.7) | 125 (40.0) | 57 (9.2) | 238 (18.6) |  |
| Agree | 48 (9.8) | 57 (42.7) | 252 (84.8) | 357 (27.9) | <0.001 |
| *Missing* | *7* | *1* | *2* | *10* |  |
| **COVID-19 vaccination should be a personal choice** |  |  |  |  |  |
| Disagree | 89 (16.9) | 29 (8.6) | 70 (53.8) | 188 (14.7) |  |
| Neutral | 74 (4.4) | 104 (54.7) | 69 (11.7) | 247 (19.3) | <0.001 |
| Agree | 519 (78.8) | 103 (36.8) | 226 (34.5) | 848 (66.1) |  |
| *Missing* | *1* | *1* | *4* | *6* |  |

Notes: Frequencies are unweighted and percentages are weighted, except for total column, in which both are unweighted.

Percentages do not sum to 100 due to rounding

**SPAIN**

**Table S37.** Sociodemographic characteristics of participants in the CANDOUR II study (Spain) by early life COVID-19 vaccine mandate attitude

| **Early life COVID-19 vaccine mandate attitude** | **Disagree**  **n=567** | **Neutral**  **n=231** | **Agree**  **n=300** | **Total**  **N=1,098** | **P-value** |
| --- | --- | --- | --- | --- | --- |
|  | Unweighted frequency **(weighted %)** | Unweighted frequency **(weighted %)** | Unweighted frequency **(weighted %)** | Unweighted frequency **(unweighted %)** |  |
| **Age (years)** |  |  |  |  | 0.35 |
| 18-24 | 23 (2.6) | 8 (3.7) | 11 (2.1) | 42 (3.8) |  |
| 25-34 | 131 (23.5) | 56 (23.4) | 42 (23.1) | 229 (20.9) |  |
| 35-44 | 129 (33.9) | 28 (10.6) | 50 (24.5) | 207 (18.9) |  |
| 45-54 | 117 (11.6) | 57 (24.9) | 67 (10.7) | 241 (21.9) |  |
| 55-64 | 101 (21.2) | 50 (22.5) | 81 (14.3) | 232 (21.1) |  |
| 65 and over | 66 (7.3) | 32 (14.9) | 49 (25.3) | 147 (13.4) |  |
| **Gender** |  |  |  |  | 0.38 |
| Woman | 315 (53.0) | 118 (51.5) | 137 (23.3) | 570 (51.9) |  |
| Man | 251 (36.6) | 113 (48.5) | 162 (59.9) | 526 (47.9) |  |
| Other/Prefer not to say | 1 (10.4) | 0 (0.0) | 1 (16.7) | 2 (0.2) |  |
| **Marital status** |  |  |  |  | 0.36 |
| Single | 198 (23.3) | 77 (32.6) | 86 (37.7) | 361 (33.6) |  |
| Not single | 358 (76.7) | 149 (67.4) | 208 (62.3) | 715 (66.4) |  |
| *Missing* | *11* | *5* | *6* | *22* |  |
| **Number of children** |  |  |  |  | 0.004 |
| 0 | 355 (53.5) | 149 (65.0) | 206 (82.4) | 710 (65.7) |  |
| 1 | 114 (36.3) | 45 (20.2) | 45 (9.1) | 204 (18.9) |  |
| 2 | 75 (8.7) | 28 (12.2) | 32 (6.3) | 135 (12.5) |  |
| 3 and over | 13 (1.5) | 6 (2.7) | 12 (2.2) | 31 (2.9) |  |
| *Missing* | *10* | *3* | *5* | *18* |  |
| **Education level** |  |  |  |  | 0.26 |
| Less than primary completed | 10 (1.1) | 7 (2.1) | 8 (1.5) | 25 (2.3) |  |
| Primary completed | 50 (5.5) | 25 (7.5) | 34 (6.2) | 109 (10.0) |  |
| Secondary completed | 177 (34.5) | 89 (48.4) | 91 (30.2) | 357 (32.7) |  |
| University completed | 327 (58.9) | 110 (41.9) | 165 (62.0) | 602 (55.1) |  |
| *Missing* | *3* | *0* | *2* | *5* |  |
| **Employment status** |  |  |  |  | 0.69 |
| Employed | 360 (46.6) | 134 (56.6) | 165 (43.5) | 659 (60.0) |  |
| Not employed | 207 (53.4) | 97 (43.4) | 135 (56.5) | 439 (40.0) |  |
| **Quintiles for PPP-adjusted equivalised gross annual income** |  |  |  |  | 0.22 |
| Quintile 1 (low) | 83 (15.2) | 37 (20.7) | 52 (21.9) | 172 (20.2) |  |
| Quintile 2 | 105 (19.7) | 44 (25.8) | 54 (23.3) | 203 (23.8) |  |
| Quintile 3 | 76 (14.1) | 26 (13.5) | 38 (16.6) | 140 (16.4) |  |
| Quintile 4 | 97 (17.9) | 41 (23.2) | 44 (19.9) | 182 (21.3) |  |
| Quintile 5 (high) | 80 (33.1) | 30 (16.8) | 46 (18.4) | 156 (18.3) |  |
| *Missing* | *126* | *53* | *66* | *245* |  |

Notes: Frequencies are unweighted and percentages are weighted, except for total column, in which both are unweighted.

Percentages do not sum to 100 due to rounding

| **Table S38.** Personal COVID-19 experience, health risk attitude, and political ideology of participants in the CANDOUR II study (Spain) by early life COVID-19 vaccine mandate attitude | | | | | |
| --- | --- | --- | --- | --- | --- |
| **Early life COVID-19 vaccine mandate attitude** | **Disagree**  **n=567** | **Neutral**  **n=231** | **Agree**  **n=300** | **Total**  **N=1,098** | **P-value** |
| **Number of participants** | Unweighted frequency **(weighted %)** | Unweighted frequency **(weighted %)** | Unweighted frequency **(weighted %)** | Unweighted frequency **(unweighted %)** |  |
| **COVID-19 vaccination status** |  |  |  |  | 0.43 |
| Vaccinated | 504 (95.4) | 208 (99.6) | 286 (100.0) | 998 (96.1) |  |
| Waiting for vaccination | 0 (0.0) | 1 (0.4) | 0 (0.0) | 1 (0.1) |  |
| Declined | 29 (3.5) | 0 (0.0) | 0 (0.0) | 29 (2.8) |  |
| Prefer not to say | 10 (1.2) | 0 (0.0) | 0 (0.0) | 10 (1.0) |  |
| *Missing* | *24* | *22* | *14* | *60* |  |
| **Side Effects** |  |  |  |  | 0.12 |
| No side effects | 417 (88.7) | 166 (81.3) | 213 (70.2) | 796 (79.8) |  |
| Side effects | 87 (11.3) | 42 (18.7) | 73 (29.8) | 202 (20.2) |  |
| Not vaccinated | 39 | 1 | 0 | 40 |  |
| *Missing* | *24* | *22* | *14* | *60* |  |
| **Reasons for Vaccinating** |  |  |  |  |  |
| To protect myself | 357 (68.8) | 158 (75.8) | 245 (59.1) | 760 (76.2) | 0.56 |
| To protect my family | 358 (68.9) | 145 (69.6) | 195 (49.9) | 698 (69.9) | 0.31 |
| To protect the public | 227 (52.7) | 75 (35.3) | 123 (71.8) | 425 (42.6) | 0.07 |
| To travel and visit people/places | 210 (38.3) | 66 (30.6) | 83 (31.7) | 359 (36.0) | 0.75 |
| Because everyone else will | 12 (0.7) | 6 (0.4) | 5 (0.4) | 23 (2.3) | 0.29 |
| Recommended by friends/family | 6 (0.9) | 7 (3.1) | 5 (0.8) | 18 (1.8) | 0.04 |
| Recommended by healthcare officials/professionals | 173 (34.0) | 87 (41.7) | 110 (36.1) | 370 (37.1) | 0.84 |
| Recommended by politicians | 7 (0.8) | 6 (2.8) | 8 (1.3) | 21 (2.1) | 0.13 |
| Contact with/symptoms of COVID-19 | 50 (18.6) | 30 (14.4) | 32 (5.3) | 112 (11.2) | 0.13 |
| Work/school requirement | 46 (5.8) | 10 (4.4) | 22 (3.7) | 78 (7.8) | 0.43 |
| Other reason(s) | 11 (1.5) | 2 (1.0) | 3 (0.5) | 16 (1.6) | 0.31 |
| **Personal health risk attitude** |  |  |  |  | 0.01 |
| Unwilling | 392 (81.1) | 99 (44.0) | 168 (46.6) | 659 (62.3) |  |
| Neutral | 131 (15.6) | 98 (43.8) | 61 (27.6) | 290 (27.4) |  |
| Willing | 29 (3.3) | 27 (12.2) | 53 (25.8) | 109 (10.3) |  |
| *Missing* | *15* | *7* | *18* | *40* |  |
| **Does participant know anyone who died of COVID-19?** |  |  |  |  | 0.59 |
| Yes | 265 (40.9) | 109 (51.3) | 147 (50.1) | 521 (49.9) |  |
| No | 276 (59.1) | 104 (48.7) | 144 (49.9) | 524 (50.1) |  |
| *Missing* | *26* | *18* | *9* | *53* |  |
| **Ideology** |  |  |  |  | 0.03 |
| Left | 230 (68.1) | 86 (37.9) | 108 (43.4) | 424 (41.0) |  |
| Centre | 200 (20.8) | 89 (42.2) | 99 (20.5) | 388 (37.5) |  |
| Right | 102 (11.1) | 43 (19.9) | 77 (36.1) | 222 (21.5) |  |
| *Missing* | *35* | *13* | *16* | *64* |  |

Note: Frequencies are unweighted and percentages are weighted, except for total column, in which both are unweighted. Percentages do not sum to 100 due to rounding

**Table S39.** Other COVID-19 vaccine mandate attitudes of participants in the CANDOUR II study (Spain) by early life COVID-19 vaccine mandate attitude

| **Early life COVID-19 vaccine mandate attitude** | **Disagree**  **n=567** | **Neutral**  **n=231** | **Agree**  **n=300** | **Total**  **N=1,098** | **P-value** |
| --- | --- | --- | --- | --- | --- |
|  | Unweighted frequency **(weighted %)** | Unweighted frequency **(weighted %)** | Unweighted frequency **(weighted %)** | Unweighted frequency **(unweighted %)** |  |
| **Schoolchild COVID-19 vaccine mandate attitude** |  |  |  |  |  |
| Disagree | 332 (67.0) | 13 (5.6) | 11 (1.7) | 356 (33.2) |  |
| Neutral | 93 (20.0) | 109 (48.6) | 32 (5.4) | 234 (21.8) | <0.001 |
| Agree | 123 (13.0) | 106 (45.7) | 252 (92.9) | 481 (44.9) |  |
| *Missing* | *19* | *3* | *5* | *27* |  |
| **Governmental COVID-19 vaccine mandate attitude** |  |  |  |  |  |
| Disagree | 393 (72.1) | 17 (7.8) | 16 (2.9) | 426 (39.5) |  |
| Neutral | 75 (18.1) | 126 (57.5) | 45 (24.3) | 246 (22.8) | <0.001 |
| Agree | 92 (9.8) | 80 (34.7) | 235 (72.8) | 407 (37.7) |  |
| *Missing* | *7* | *8* | *4* | *19* |  |
| **COVID-19 vaccination should be a personal choice** |  |  |  |  |  |
| Disagree | 160 (30.1) | 45 (20.5) | 121 (20.4) | 326 (30.2) |  |
| Neutral | 90 (10.4) | 117 (52.2) | 61 (27.4) | 268 (24.8) | 0.04 |
| Agree | 314 (59.5) | 64 (27.3) | 108 (52.2) | 486 (45.0) |  |
| *Missing* | *3* | *5* | *10* | *18* |  |

Notes: Frequencies are unweighted and percentages are weighted, except for total column, in which both are unweighted.

Percentages do not sum to 100 due to rounding

**UGANDA**

**Table S40.** Sociodemographic characteristics of participants in the CANDOUR II study (Uganda) by early life COVID-19 vaccine mandate attitude

| **Early life COVID-19 vaccine mandate attitude** | **Disagree**  **n=690** | **Neutral**  **n=201** | **Agree**  **n=358** | **Total**  **N=1,249** | **P-value** |
| --- | --- | --- | --- | --- | --- |
|  | Unweighted frequency **(weighted %)** | Unweighted frequency **(weighted %)** | Unweighted frequency **(weighted %)** | Unweighted frequency **(unweighted %)** |  |
| **Age (years)** |  |  |  |  | 0.56 |
| 18-24 | 110 (24.5) | 47 (31.5) | 90 (21.9) | 247 (19.8) |  |
| 25-34 | 389 (46.0) | 107 (63.8) | 196 (39.4) | 692 (55.4) |  |
| 35-44 | 148 (18.8) | 39 (3.3) | 56 (19.6) | 243 (19.5) |  |
| 45-54 | 32 (4.7) | 7 (1.4) | 13 (17.7) | 52 (4.2) |  |
| 55-64 | 8 (0.4) | 1 (0.003) | 2 (1.4) | 11 (0.9) |  |
| 65 and over | 3 (5.7) | 0 (0.0) | 1 (0.0003) | 4 (0.3) |  |
| **Gender** |  |  |  |  | 0.03 |
| Woman | 239 (16.3) | 52 (2.5) | 108 (21.9) | 399 (31.9) |  |
| Man | 448 (68.8) | 149 (97.5) | 247 (31.8) | 844 (67.6) |  |
| Other/Prefer not to say | 3 (15.0) | 0 (0.0) | 3 (46.3) | 6 (0.5) |  |
| **Marital status** |  |  |  |  | 0.39 |
| Single | 343 (63.0) | 102 (54.0) | 196 (81.2) | 641 (52.9) |  |
| Not single | 322 (37.0) | 94 (46.0) | 155 (18.8) | 571 (47.1) |  |
| *Missing* | *25* | *5* | *7* | *37* |  |
| **Number of children** |  |  |  |  | 0.29 |
| 0 | 184 (22.6) | 59 (32.1) | 111 (42.3) | 354 (29.0) |  |
| 1 | 75 (15.2) | 30 (32.9) | 39 (3.9) | 144 (11.8) |  |
| 2 | 152 (28.1) | 31 (6.4) | 88 (9.0) | 271 (22.2) |  |
| 3 and over | 260 (34.1) | 75 (28.5) | 116 (44.8) | 451 (37.0) |  |
| *Missing* | *19* | *6* | *4* | *29* |  |
| **Education level** |  |  |  |  | <0.001 |
| Less than primary completed | 1 (1.8) | 2 (62.6) | 0 (0.0) | 3 (0.2) |  |
| Primary completed | 14 (9.1) | 9 (7.0) | 20 (23.9) | 43 (3.5) |  |
| Secondary completed | 213 (67.5) | 67 (28.1) | 122 (54.8) | 402 (32.4) |  |
| University completed | 458 (21.6) | 121 (2.3) | 215 (21.3) | 794 (63.9) |  |
| *Missing* | *4* | *2* | *1* | *7* |  |
| **Employment status** |  |  |  |  | 0.43 |
| Employed | 499 (75.2) | 136 (65.2) | 225 (53.4) | 860 (68.9) |  |
| Not employed | 191 (24.8) | 65 (34.8) | 133 (47.6) | 389 (31.1) |  |
| **Quintiles for PPP-adjusted equivalised gross annual income** |  |  |  |  | 0.47 |
| Quintile 1 (low) | 107 (30.5) | 52 (28.0) | 73 (52.7) | 232 (20.6) |  |
| Quintile 2 | 146 (33.8) | 42 (38.2) | 80 (10.3) | 268 (23.8) |  |
| Quintile 3 | 107 (11.4) | 31 (4.8) | 58 (25.2) | 196 (17.4) |  |
| Quintile 4 | 127 (15.9) | 37 (25.8) | 52 (5.6) | 216 (19.2) |  |
| Quintile 5 (high) | 127 (8.3) | 27 (3.1) | 58 (6.2) | 212 (18.9) |  |
| *Missing* | *76* | *12* | *37* | *125* |  |

Notes: Frequencies are unweighted and percentages are weighted, except for total column, in which both are unweighted.

Percentages do not sum to 100 due to rounding

| **Table S41.** Personal COVID-19 experience, health risk attitude, and political ideology of participants in the CANDOUR II study (Uganda) by early life COVID-19 vaccine mandate attitude | | | | | |
| --- | --- | --- | --- | --- | --- |
| **Early life COVID-19 vaccine mandate attitude** | **Disagree**  **n=690** | **Neutral**  **n=201** | **Agree**  **n=358** | **Total**  **N=1,249** | **P-value** |
| **Number of participants** | Unweighted frequency **(weighted %)** | Unweighted frequency **(weighted %)** | Unweighted frequency **(weighted %)** | Unweighted frequency **(unweighted %)** |  |
| **COVID-19 vaccination status** |  |  |  |  | <0.001 |
| Vaccinated | 549 (97.9) | 172 (99.6) | 319 (99.7) | 1,040 (90.7) |  |
| Waiting for vaccination | 20 (1.0) | 6 (0.1) | 6 (0.1) | 32 (2.8) |  |
| Declined | 50 (0.8) | 3 (0.2) | 6 (0.1) | 59 (5.1) |  |
| Prefer not to say | 10 (0.3) | 2 (0.001) | 4 (0.001) | 16 (1.4) |  |
| *Missing* | *61* | *18* | *23* | *102* |  |
| **Side Effects** |  |  |  |  | 0.86 |
| No side effects | 443 (91.5) | 131 (94.2) | 247 (90.5) | 821 (78.9) |  |
| Side effects | 106 (8.5) | 41 (5.8) | 72 (9.5) | 219 (21.1) |  |
| Not vaccinated | 80 | 11 | 16 | 107 |  |
| *Missing* | *61* | *18* | *23* | *102* |  |
| **Reasons for Vaccinating** |  |  |  |  |  |
| To protect myself | 385 (73.2) | 128 (53.8) | 256 (61.4) | 769 (73.9) | 0.66 |
| To protect my family | 307 (43.2) | 99 (53.4) | 180 (19.0) | 586 (56.3) | 0.28 |
| To protect the public | 219 (28.6) | 71 (11.5) | 129 (30.6) | 419 (40.3) | 0.50 |
| To travel and visit people/places | 204 (34.2) | 52 (18.9) | 112 (12.4) | 368 (35.4) | 0.13 |
| Because everyone else will | 16 (3.7) | 10 (5.4) | 16 (0.4) | 42 (4.0) | 0.03 |
| Recommended by friends/family | 42 (1.0) | 16 (2.4) | 37 (5.5) | 95 (9.1) | 0.04 |
| Recommended by healthcare officials/professionals | 142 (14.7) | 45 (37.6) | 85 (11.5) | 272 (26.2) | 0.29 |
| Recommended by politicians | 26 (6.2) | 11 (3.5) | 23 (2.2) | 60 (5.8) | 0.49 |
| Contact with/symptoms of COVID-19 | 52 (7.6) | 16 (2.8) | 30 (5.5) | 98 (9.4) | 0.63 |
| Work/school requirement | 172 (27.0) | 50 (8.2) | 82 (23.4) | 304 (29.2) | 0.45 |
| Other reason(s) | 6 (0.6) | 4 (0.1) | 2 (0.5) | 12 (1.2) | 0.60 |
| **Personal health risk attitude** |  |  |  |  | 0.08 |
| Unwilling | 454 (54.9) | 105 (38.5) | 202 (83.3) | 761 (61.6) |  |
| Neutral | 135 (37.7) | 62 (28.0) | 67 (8.7) | 264 (21.4) |  |
| Willing | 94 (7.4) | 32 (33.5) | 85 (8.1) | 211 (17.1) |  |
| *Missing* | *7* | *2* | *4* | *13* |  |
| **Does participant know anyone who died of COVID-19?** |  |  |  |  | 0.02 |
| Yes | 442 (66.7) | 125 (18.8) | 238 (66.5) | 805 (66.3) |  |
| No | 228 (33.3) | 70 (81.2) | 111 (33.5) | 409 (33.7) |  |
| *Missing* | *20* | *6* | *9* | *35* |  |
| **Ideology** |  |  |  |  | 0.03 |
| Left | 165 (29.5) | 48 (5.6) | 73 (6.1) | 286 (22.9) |  |
| Centre | 356 (40.5) | 105 (82.4) | 168 (48.3) | 629 (50.4) |  |
| Right | 169 (30.1) | 48 (12.0) | 117 (45.6) | 334 (26.7) |  |
| *Missing* | *0* | *0* | *0* | *0* |  |

Note: Frequencies are unweighted and percentages are weighted, except for total column, in which both are unweighted. Percentages do not sum to 100 due to rounding

**Table S42.** Other COVID-19 vaccine mandate attitudes of participants in the CANDOUR II study (Uganda) by early life COVID-19 vaccine mandate attitude

| **Early life COVID-19 vaccine mandate attitude** | **Disagree**  **n=690** | **Neutral**  **n=201** | **Agree**  **n=358** | **Total**  **N=1,249** | **P-value** |
| --- | --- | --- | --- | --- | --- |
|  | Unweighted frequency **(weighted %)** | Unweighted frequency **(weighted %)** | Unweighted frequency **(weighted %)** | Unweighted frequency **(unweighted %)** |  |
| **Schoolchild COVID-19 vaccine mandate attitude** |  |  |  |  |  |
| Disagree | 436 (68.0) | 40 (24.2) | 55 (2.1) | 531 (42.7) |  |
| Neutral | 138 (8.7) | 80 (62.3) | 66 (6.1) | 284 (22.8) | <0.001 |
| Agree | 113 (23.3) | 80 (13.5) | 235 (91.8) | 428 (34.4) |  |
| *Missing* | *3* | *1* | *2* | *6* |  |
| **Governmental COVID-19 vaccine mandate attitude** |  |  |  |  |  |
| Disagree |  |  |  |  |  |
| Neutral | 485 (63.1) | 33 (2.6) | 60 (2.8) | 578 (46.5) |  |
| Agree | 117 (18.6) | 97 (88.3) | 51 (2.3) | 265 (21.3) | <0.001 |
| *Missing* | 85 (18.2) | 71 (9.1) | 245 (94.8) | 401 (32.2) |  |
| **COVID-19 vaccination should be a personal choice** | *3* | *0* | *2* | *5* |  |
| Disagree | 157 (14.2) | 49 (7.6) | 127 (62.5) | 333 (26.8) |  |
| Neutral | 125 (30.6) | 96 (88.3) | 79 (6.6) | 300 (24.2) | <0.001 |
| Agree | 408 (55.2) | 55 (4.0) | 146 (30.9) | 609 (49.0) |  |
| *Missing* | *0* | *1* | *6* | *7* |  |

Notes: Frequencies are unweighted and percentages are weighted, except for total column, in which both are unweighted.

Percentages do not sum to 100 due to rounding

**UNITED KINGDOM**

**Table S43.** Sociodemographic characteristics of participants in the CANDOUR II study (U.K.) by early life COVID-19 vaccine mandate attitude

| **Early life COVID-19 vaccine mandate attitude** | **Disagree**  **n=543** | **Neutral**  **n=261** | **Agree**  **n=345** | **Total**  **N=1,149** | **P-value** |
| --- | --- | --- | --- | --- | --- |
|  | Unweighted frequency **(weighted %)** | Unweighted frequency **(weighted %)** | Unweighted frequency **(weighted %)** | Unweighted frequency **(unweighted %)** |  |
| **Age (years)** |  |  |  |  | 0.51 |
| 18-24 | 26 (12.2) | 16 (3.6) | 16 (13.3) | 58 (5.0) |  |
| 25-34 | 92 (11.3) | 55 (9.0) | 44 (15.9) | 191 (16.6) |  |
| 35-44 | 86 (29.6) | 41 (21.2) | 65 (17.1) | 192 (16.7) |  |
| 45-54 | 99 (25.2) | 55 (38.4) | 73 (28.7) | 227 (19.8) |  |
| 55-64 | 123 (5.9) | 58 (23.0) | 85 (19.1) | 266 (23.2) |  |
| 65 and over | 117 (15.9) | 36 (4.8) | 62 (6.0) | 215 (18.7) |  |
| **Gender** |  |  |  |  | 0.92 |
| Woman | 290 (51.6) | 118 (33.3) | 156 (48.9) | 564 (49.1) |  |
| Man | 251 (38.2) | 142 (51.5) | 188 (40.4) | 581 (50.6) |  |
| Other/Prefer not to say | 2 (10.2) | 1 (15.3) | 1 (10.7) | 4 (0.3) |  |
| **Marital status** |  |  |  |  | 0.38 |
| Single | 207 (63.8) | 95 (44.7) | 115 (45.0) | 417 (36.6) |  |
| Not single | 328 (36.2) | 166 (55.3) | 229 (55.0) | 723 (63.4) |  |
| *Missing* | *8* | *0* | *1* | *9* |  |
| **Number of children** |  |  |  |  | 0.05 |
| 0 | 372 (67.9) | 180 (57.6) | 249 (69.0) | 801 (70.2) |  |
| 1 | 87 (5.1) | 32 (35.1) | 43 (15.3) | 162 (14.2) |  |
| 2 | 62 (20.5) | 32 (4.7) | 38 (3.9) | 132 (11.6) |  |
| 3 and over | 16 (6.5) | 17 (2.6) | 13 (11.8) | 46 (4.0) |  |
| *Missing* | *6* | *0* | *2* | *8* |  |
| **Education level** |  |  |  |  | 0.07 |
| Less than primary completed | 16 (0.8) | 7 (0.9) | 11 (1.1) | 34 (3.0) |  |
| Primary completed | 49 (21.4) | 25 (3.1) | 35 (3.6) | 109 (9.6) |  |
| Secondary completed | 298 (44.2) | 110 (71.7) | 166 (54.7) | 574 (50.6) |  |
| University completed | 171 (33.6) | 117 (24.3) | 129 (40.6) | 417 (36.8) |  |
| *Missing* | *9* | *2* | *4* | *15* |  |
| **Employment status** |  |  |  |  | 0.27 |
| Employed | 318 (43.0) | 169 (71.7) | 194 (53.2) | 681 (59.3) |  |
| Not employed | 225 (57.0) | 92 (28.3) | 151 (46.8) | 468 (40.7) |  |
| **Quintiles for PPP-adjusted equivalised gross annual income** |  |  |  |  | 0.21 |
| Quintile 1 (low) | 107 (56.3) | 58 (39.4) | 59 (58.0) | 224 (21.5) |  |
| Quintile 2 | 104 (13.2) | 37 (21.3) | 56 (8.9) | 197 (18.9) |  |
| Quintile 3 | 93 (6.1) | 55 (24.6) | 74 (11.9) | 222 (21.3) |  |
| Quintile 4 | 92 (18,6) | 43 (6.6) | 52 (9.4) | 187 (18.0) |  |
| Quintile 5 (high) | 85 (5.9) | 54 (8.1) | 71 (11.7) | 210 (20.2) |  |
| *Missing* | *62* | *14* | *33* | *109* |  |

Notes: Frequencies are unweighted and percentages are weighted, except for total column, in which both are unweighted.

Percentages do not sum to 100 due to rounding

| **Table S44.** Personal COVID-19 experience, health risk attitude, and political ideology of participants in the CANDOUR II study (U.K.) by early life COVID-19 vaccine mandate attitude | | | | | |
| --- | --- | --- | --- | --- | --- |
| **Early life COVID-19 vaccine mandate attitude** | **Disagree**  **n=543** | **Neutral**  **n=261** | **Agree**  **n=345** | **Total**  **N=1,149** | **P-value** |
| **Number of participants** | Unweighted frequency **(weighted %)** | Unweighted frequency **(weighted %)** | Unweighted frequency **(weighted %)** | Unweighted frequency **(unweighted %)** |  |
| **COVID-19 vaccination status** |  |  |  |  | 0.02 |
| Vaccinated | 447 (73.0) | 233 (99.3) | 330 (99.2) | 1,010 (92.8) |  |
| Waiting for vaccination | 1 (0.001) | 1 (0.2) | 2 (0.2) | 4 (0.4) |  |
| Declined | 63 (21.1) | 3 (0.5) | 4 (0.6) | 70 (6.4) |  |
| Prefer not to say | 3 (5.9) | 1 (0.1) | 0 (0.0) | 4 (0.4) |  |
| *Missing* | *29* | *23* | *9* | *61* |  |
| **Side Effects** |  |  |  |  | 0.31 |
| No side effects | 324 (82.5) | 167 (58.3) | 218 (77.5) | 709 (70.2) |  |
| Side effects | 123 (17.5) | 66 (41.7) | 112 (22.5) | 301 (29.8) |  |
| Not vaccinated | 67 | 5 | 6 | 78 |  |
| *Missing* | *29* | *23* | *9* | *61* |  |
| **Reasons for Vaccinating** |  |  |  |  |  |
| To protect myself | 352 (83.5) | 191 (92.8) | 291 (74.8) | 834 (82.6) | 0.31 |
| To protect my family | 330 (66.1) | 187 (92.0) | 270 (72.7) | 787 (77.9) | 0.18 |
| To protect the public | 233 (50.1) | 136 (52.4) | 192 (42.8) | 561 (55.5) | 0.87 |
| To travel and visit people/places | 213 (41.8) | 116 (50.3) | 173 (41.1) | 502 (49.7) | 0.88 |
| Because everyone else will | 25 (1.0) | 20 (0.7) | 35 (1.2) | 80 (7.9) | 0.42 |
| Recommended by friends/family | 15 (9.1) | 24 (3.9) | 28 (3.1) | 67 (6.6) | 0.28 |
| Recommended by healthcare officials/professionals | 212 (41.1) | 109 (48.6) | 165 (39.8) | 486 (48.1) | 0.90 |
| Recommended by politicians | 33 (2.7) | 30 (4.5) | 53 (5.7) | 116 (11.5) | 0.19 |
| Contact with/symptoms of COVID-19 | 52 (12.1) | 37 (5.8) | 70 (7.6) | 159 (15.7) | 0.46 |
| Work/school requirement | 49 (4.4) | 16 (2.8) | 30 (3.7) | 95 (9.4) | 0.66 |
| Other reason(s) | 11 (9.0) | 2 (0.4) | 2 (0.2) | 15 (1.5) | <0.001 |
| **Personal health risk attitude** |  |  |  |  | 0.30 |
| Unwilling | 316 (42.2) | 112 (62.8) | 208 (32.8) | 636 (56.4) |  |
| Neutral | 166 (29.6) | 112 (32.5) | 75 (29.2) | 353 (31.3) |  |
| Willing | 55 (28.2) | 28 (4.8) | 56 (38.0) | 139 (12.3) |  |
| *Missing* | *6* | *9* | *6* | *21* |  |
| **Does participant know anyone who died of COVID-19?** |  |  |  |  | 0.36 |
| Yes | 147 (21.2) | 82 (15.1) | 118 (33.6) | 347 (31.1) |  |
| No | 380 (78.8) | 170 (84.9) | 220 (66.4) | 770 (68.9) |  |
| *Missing* | *16* | *9* | *7* | *32* |  |
| **Ideology** |  |  |  |  | 0.02 |
| Left | 118 (48.5) | 47 (9.0) | 39 (7.0) | 204 (20.6) |  |
| Centre | 236 (32.1) | 135 (64.1) | 180 (62.7) | 551 (55.5) |  |
| Right | 104 (19.4) | 47 (26.9) | 86 (30.4) | 237 (23.9) |  |
| *Missing* | *85* | *32* | *40* | *157* |  |

Note: Frequencies are unweighted and percentages are weighted, except for total column, in which both are unweighted. Percentages do not sum to 100 due to rounding

**Table S45.** Other COVID-19 vaccine mandate attitudes of participants in the CANDOUR II study (U.K.) by early life COVID-19 vaccine mandate attitude

| **Early life COVID-19 vaccine mandate attitude** | **Disagree**  **n=543** | **Neutral**  **n=261** | **Agree**  **n=345** | **Total**  **N=1,149** | **P-value** |
| --- | --- | --- | --- | --- | --- |
|  | Unweighted frequency **(weighted %)** | Unweighted frequency **(weighted %)** | Unweighted frequency **(weighted %)** | Unweighted frequency **(unweighted %)** |  |
| **Schoolchild COVID-19 vaccine mandate attitude** |  |  |  |  |  |
| Disagree | 344 (67.2) | 22 (18.6) | 17 (2.0) | 383 (34.9) |  |
| Neutral | 92 (22.6) | 143 (37.4) | 47 (17.9) | 282 (25.7) | <0.001 |
| Agree | 75 (10.3) | 86 (44.0) | 271 (80.1) | 432 (39.4) |  |
| *Missing* | *32* | *10* | *10* | *52* |  |
| **Governmental COVID-19 vaccine mandate attitude** |  |  |  |  |  |
| Disagree | 403 (73.0) | 35 (5.9) | 28 (24.5) | 466 (41.4) |  |
| Neutral | 69 (18.9) | 133 (51.4) | 35 (14.3) | 237 (21.0) | <0.001 |
| Agree | 66 (8.1) | 83 (42.7) | 274 (61.2) | 423 (37.6) |  |
| *Missing* | *5* | *10* | *8* | *23* |  |
| **COVID-19 vaccination should be a personal choice** |  |  |  |  |  |
| Disagree | 134 (27.2) | 62 (39.9) | 145 (36.9) | 341 (30.2) |  |
| Neutral | 105 (15.5) | 134 (21.2) | 70 (18.5) | 309 (27.4) | 0.82 |
| Agree | 301 (57.3) | 58 (38.9) | 120 (44.6) | 479 (42.4) |  |
| *Missing* | *3* | *7* | *10* | *20* |  |

Notes: Frequencies are unweighted and percentages are weighted, except for total column, in which both are unweighted.

Percentages do not sum to 100 due to rounding

**UNITED STATES**

**Table S46.** Sociodemographic characteristics of participants in the CANDOUR II study (United States) by early life COVID-19 vaccine mandate attitude

| **Early life COVID-19 vaccine mandate attitude** | **Disagree**  **n=486** | **Neutral**  **n=255** | **Agree**  **n=447** | **Total**  **N=1,188** | **P-value** |
| --- | --- | --- | --- | --- | --- |
|  | Unweighted frequency **(weighted %)** | Unweighted frequency **(weighted %)** | Unweighted frequency **(weighted %)** | Unweighted frequency **(unweighted %)** |  |
| **Age (years)** |  |  |  |  | 0.13 |
| 18-24 | 34 (18.7) | 21 (10.6) | 57 (30.7) | 112 (9.4) |  |
| 25-34 | 102 (13.2) | 63 (25.0) | 100 (18.1) | 265 (22.3) |  |
| 35-44 | 101 (10.7) | 87 (27.5) | 137 (21.9) | 325 (27.4) |  |
| 45-54 | 85 (24.6) | 42 (19.9) | 60 (12.5) | 187 (15.7) |  |
| 55-64 | 89 (11.7) | 21 (8.6) | 52 (10.1) | 162 (13.6) |  |
| 65 and over | 75 (21.2) | 21 (8.4) | 41 (6.8) | 137 (11.5) |  |
| **Gender** |  |  |  |  | 0.32 |
| Woman | 315 (49.1) | 132 (48.5) | 206 (35.3) | 653 (55.0) |  |
| Man | 169 (25.6) | 123 (51.5) | 240 (46.1) | 532 (44.8) |  |
| Other/Prefer not to say | 2 (25.3) | 0 (0.0) | 1 (18.6) | 3 (0.3) |  |
| **Marital status** |  |  |  |  | 0.22 |
| Single | 188 (51.2) | 89 (38.3) | 157 (36.6) | 434 (36.8) |  |
| Not single | 292 (48.8) | 165 (61.7) | 287 (63.4) | 744 (63.2) |  |
| *Missing* | *6* | *1* | *31* | *0* |  |
| **Number of children** |  |  |  |  | 0.52 |
| 0 | 289 (63.4) | 142 (60.5) | 233 (61.3) | 664 (56.5) |  |
| 1 | 96 (24.7) | 62 (21.1) | 109 (19.3) | 267 (22.7) |  |
| 2 | 56 (7.1) | 30 (13.3) | 79 (15.1) | 165 (14.0) |  |
| 3 and over | 41 (4.9) | 15 (5.1) | 24 (4.3) | 80 (6.8) |  |
| *Missing* | *4* | *6* | *2* | *12* |  |
| **Education level** |  |  |  |  | 0.05 |
| Less than primary completed | 4 (1.6) | 0 (0.0) | 0 (0.0) | 4 (0.3) |  |
| Primary completed | 16 (20.7) | 8 (8.4) | 8 (4.1) | 32 (2.7) |  |
| Secondary completed | 313 (49.0) | 117 (38.1) | 223 (51.3) | 653 (55.0) |  |
| University completed | 152 (28.7) | 130 (53.5) | 216 (44.6) | 498 (42.0) |  |
| *Missing* | *1* | *0* | *0* | *1* |  |
| **Employment status** |  |  |  |  | 0.03 |
| Employed | 284 (51.2) | 161 (62.2) | 318 (78.4) | 763 (64.2) |  |
| Not employed | 202 (48.8) | 94 (37.8) | 129 (21.6) | 425 (35.8) |  |
| **Quintiles for PPP-adjusted equivalised gross annual income** |  |  |  |  | 0.48 |
| Quintile 1 (low) | 83 (24.1) | 42 (17.1) | 92 (18.1) | 217 (19.1) |  |
| Quintile 2 | 105 (14.1) | 41 (15.5) | 92 (15.9) | 238 (20.9) |  |
| Quintile 3 | 96 (24.1) | 42 (21.2) | 88 (16.3) | 226 (19.9) |  |
| Quintile 4 | 120 (29.5) | 48 (20.3) | 79 (33.1) | 247 (21.7) |  |
| Quintile 5 (high) | 58 (8.3) | 69 (25.8) | 83 (16.6) | 210 (18.5) |  |
| *Missing* | *24* | *13* | *13* | *50* |  |

Notes: Frequencies are unweighted and percentages are weighted, except for total column, in which both are unweighted.

Percentages do not sum to 100 due to rounding

| **Table S47.** Personal COVID-19 experience, health risk attitude, and political ideology of participants in the CANDOUR II study (United States) by early life COVID-19 vaccine mandate attitude | | | | | |
| --- | --- | --- | --- | --- | --- |
| **Early life COVID-19 vaccine mandate attitude** | **Disagree**  **n=486** | **Neutral**  **n=255** | **Agree**  **n=447** | **Total**  **N=1,188** | **P-value** |
| **Number of participants** | Unweighted frequency **(weighted %)** | Unweighted frequency **(weighted %)** | Unweighted frequency **(weighted %)** | Unweighted frequency **(unweighted %)** |  |
| **COVID-19 vaccination status** |  |  |  |  | <0.001 |
| Vaccinated | 262 (71.2) | 178 (89.5) | 373 (96.4) | 813 (80.8) |  |
| Waiting for vaccination | 7 (1.4) | 6 (3.4) | 7 (1.4) | 20 (2.0) |  |
| Declined | 135 (25.8) | 15 (6.5) | 12 (2.0) | 162 (16.1) |  |
| Prefer not to say | 8 (1.6) | 1 (0.6) | 2 (0.2) | 11 (1.1) |  |
| *Missing* | *74* | *55* | *53* | *182* |  |
| **Side Effects** |  |  |  |  | 0.37 |
| No side effects | 192 (53.3) | 111 (64.7) | 226 (69.6) | 529 (65.1) |  |
| Side effects | 70 (46.7) | 67 (35.3) | 147 (30.4) | 284 (34.9) |  |
| Not vaccinated | 150 | 22 | 21 | 193 |  |
| *Missing* | *74* | *55* | *53* | *182* |  |
| **Reasons for Vaccinating** |  |  |  |  |  |
| To protect myself | 183 (78.0) | 145 (77.4) | 290 (82.8) | 618 (76.0) | 0.63 |
| To protect my family | 180 (48.8) | 132 (74.6) | 281 (80.1) | 593 (72.9) | 0.02 |
| To protect the public | 110 (30.0) | 70 (42.8) | 198 (63.4) | 378 (46.5) | 0.01 |
| To travel and visit people/places | 116 (59.2) | 68 (40.5) | 217 (67.4) | 401 (49.3) | 0.15 |
| Because everyone else will | 13 (1.3) | 7 (0.7) | 38 (3.9) | 58 (7.1) | 0.09 |
| Recommended by friends/family | 32 (7.8) | 23 (11.8) | 53 (12.3) | 108 (13.3) | 0.40 |
| Recommended by healthcare officials/professionals | 111 (29.5) | 87 (47.9) | 181 (59.7) | 379 (46.6) | 0.02 |
| Recommended by politicians | 7 (1.8) | 12 (6.9) | 32 (7.2) | 51 (6.3) | 0.02 |
| Contact with/symptoms of COVID-19 | 37 (9.3) | 17 (10.9) | 93 (19.3) | 147 (18.1) | 0.07 |
| Work/school requirement | 45 (14.1) | 12 (7.9) | 50 (33.7) | 107 (13.2) | 0.05 |
| Other reason(s) | 12 (2.9) | 3 (2.8) | 3 (0.6) | 18 (2.2) | 0.14 |
| **Personal health risk attitude** |  |  |  |  | 0.01 |
| Unwilling | 285 (62.2) | 95 (40.6) | 186 (53.8) | 566 (49.0) |  |
| Neutral | 126 (30.2) | 100 (39.9) | 87 (16.1) | 313 (27.1) |  |
| Willing | 61 (7.7) | 52 (19.5) | 163 (30.0) | 276 (23.9) |  |
| *Missing* | *14* | *8* | *11* | *33* |  |
| **Does participant know anyone who died of COVID-19?** |  |  |  |  | 0.11 |
| Yes | 178 (27.2) | 93 (38.0) | 236 (45.1) | 507 (44.0) |  |
| No | 291 (72.8) | 151 (62.0) | 204 (54.9) | 646 (56.0) |  |
| *Missing* | *17* | *11* | *7* | *35* |  |
| **Ideology** |  |  |  |  | 0.27 |
| Left | 102 (27.0) | 32 (15.1) | 90 (34.8) | 224 (18.9) |  |
| Centre | 191 (36.1) | 149 (57.3) | 134 (24.5) | 474 (39.9) |  |
| Right | 193 (36.9) | 74 (27.6) | 223 (40.7) | 490 (41.2) |  |
| *Missing* | *0* | *0* | *0* | *0* |  |

Note: Frequencies are unweighted and percentages are weighted, except for total column, in which both are unweighted. Percentages do not sum to 100 due to rounding

**Table S48.** Other COVID-19 vaccine mandate attitudes of participants in the CANDOUR II study (United States) by early life COVID-19 vaccine mandate attitude

| **Early life COVID-19 vaccine mandate attitude** | **Disagree**  **n=486** | **Neutral**  **n=255** | **Agree**  **n=447** | **Total**  **N=1,188** | **P-value** |
| --- | --- | --- | --- | --- | --- |
|  | Unweighted frequency **(weighted %)** | Unweighted frequency **(weighted %)** | Unweighted frequency **(weighted %)** | Unweighted frequency **(unweighted %)** |  |
| **Schoolchild COVID-19 vaccine mandate attitude** |  |  |  |  |  |
| Disagree | 342 (82.9) | 20 (8.4) | 25 (4.3) | 387 (33.3) |  |
| Neutral | 72 (10.3) | 141 (55.8) | 47 (8.3) | 260 (22.4) | <0.001 |
| Agree | 54 (6.8) | 89 (35.9) | 372 (87.4) | 515 (44.3) |  |
| *Missing* | *18* | *5* | *3* | *26* |  |
| **Governmental COVID-19 vaccine mandate attitude** |  |  |  |  |  |
| Disagree | 415 (91.3) | 43 (19.9) | 37 (7.0) | 495 (41.9) |  |
| Neutral | 43 (5.7) | 151 (57.0) | 49 (9.1) | 243 (20.6) | <0.001 |
| Agree | 25 (2.9) | 58 (23.1) | 359 (84.0) | 442 (37.5) |  |
| *Missing* | *3* | *3* | *2* | *8* |  |
| **COVID-19 vaccination should be a personal choice** |  |  |  |  |  |
| Disagree | 95 (12.8) | 35 (14.6) | 112 (39.0) | 242 (20.6) |  |
| Neutral | 52 (7.2) | 136 (53.0) | 52 (9.5) | 240 (20.4) | <0.001 |
| Agree | 336 (80.0) | 82 (32.4) | 276 (51.5) | 694 (59.0) |  |
| *Missing* | *3* | *2* | *7* | *12* |  |

Notes: Frequencies are unweighted and percentages are weighted, except for total column, in which both are unweighted.

Percentages do not sum to 100 due to rounding
